# Photocatalytic Nitrate Reduction to Ammonia via Zr‐Mediated Proton‐Coupled Electron Transfer

**DOI:** 10.1002/cssc.202402630

**Published:** 2025-04-04

**Authors:** Pedro J. Jabalera‐Ortiz, Alvaro M. Rodriguez‐Jimenez, Pablo Garrido‐Barros

**Affiliations:** ^1^ Departamento de Química Inorgánica Facultad de Ciencias Universidad de Granada and Unidad de Excelencia en Química (UEQ) Avda. Fuente Nueva s/n, 18071 Granada Spain

**Keywords:** nitrate reduction, photoredox catalysis, homogeneous catalysis, reaction mechanisms, proton-coupled electron transfer

## Abstract

The reduction of nitrate (NO_3_^−^) is a fundamentally exciting reaction with important environmental implications. From a mechanistic perspective, it involves the transfer of 8 e^−^ and 9 H^+^, with the initial activation of NO_3_^−^ representing a significant challenge. Here we propose a distinct and competitive mechanism for the redox activation of this inert anion based on photocatalytic proton‐coupled electron transfer (PCET). The use of a PCET mediator based on a Zr coordination cage enabled formation of NH_3_ under visible light irradiation (440 nm). Importantly, the incorporation of Li^+^ as a Lewis acid within the cage structure further generated highly reactive sites that pre‐associate and activate NO_3_^−^, enhancing the catalytic activity. We also show how the back oxidation of the intermediate NO_2_^−^ has a dramatic impact in the efficiency and highlight the role of the sacrificial electron donor in outcompeting this side reaction. These aspects were finally combined with the use of silver as a d‐block metal catalyst to facilitate the NO_3_^−^ to NO_2_^−^ reduction step, the identified bottleneck of the overall process.

## Graphical abstract

Integration of a photochemical proton‐coupled electron transfer mediator with Li^+^ as a Lewis acid provides a powerful platform for the redox activation of nitrate and the generation of ammonia. Considerations of the sacrificial electron donor and incorporation of a d‐block metal co‐catalyst enables the enhancement of the kinetics and efficiency of the catalytic reaction.

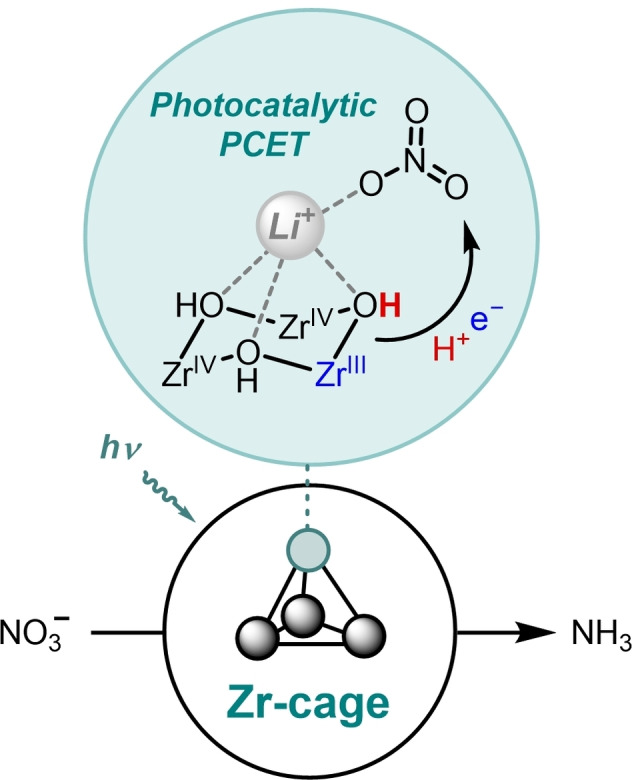

## Introduction

Nitrogen compounds are central to many chemical reactions in both biological systems and industrial processes, with applications that span the synthesis of drugs, fertilizers, functional materials, and potential green fuels. Understanding the conversion between oxidized and reduced nitrogen is thus key to developing sustainable routes and establishing a circular economy for the manufacture of nitrogen containing molecules.^[1]^ In this context, the reduction of oxidized nitrogen, and particularly nitrate (NO_3_^−^), has drawn special attention due to the associated environmental implications.^[2]^ NO_3_^−^ is a primary source of nitrogen for plants and thus, is widely used in the preparation of fertilizers. However, the intensive agricultural activity, amongst other anthropogenic sources, has triggered its excessive accumulation in aqueous ecosystems, causing eutrophication and compromising the life of many aquatic species.[^3^, ^4^, ^5^] These issues have motivated the development of new remediation technologies based on either separation or transformation of the NO_3_^−^ into other innocuous nitrogenous products (e. g., N_2_).[^6^, ^7^, ^8^] An exciting alternative involves the revalorization of NO_3_^−^ by its selective electrochemical reduction to NH_3_,[^9^, ^10^, ^11^] a pivotal nitrogen compound in the chemical industry and a promising energy vector and H_2_ carrier.[^12^, ^13^, ^14^, ^15^]

From a fundamental viewpoint, the reduction of NO_3_^−^ to NH_3_ stands out as a challenging process due to the multiple bond breaking and forming steps, involving the overall transfer of 8 e^−^/9 H^+^. While the thermodynamic potential of this reaction is highly positive (1.20 V vs NHE), the initial one‐electron activation of NO_3_^−^ requires a harsh reduction potential of −0.90 V vs NHE, highlighting the energetic demand of this transformation.[^16^, ^17^] In fact, the reduction of NO_3_^−^ to NO_2_^−^ is identified as the bottleneck of the process.^[7]^ Under these reducing conditions, selectivity against competing H_2_ evolution is an additional challenge.[^2^, ^16^]

**Figure 1:**
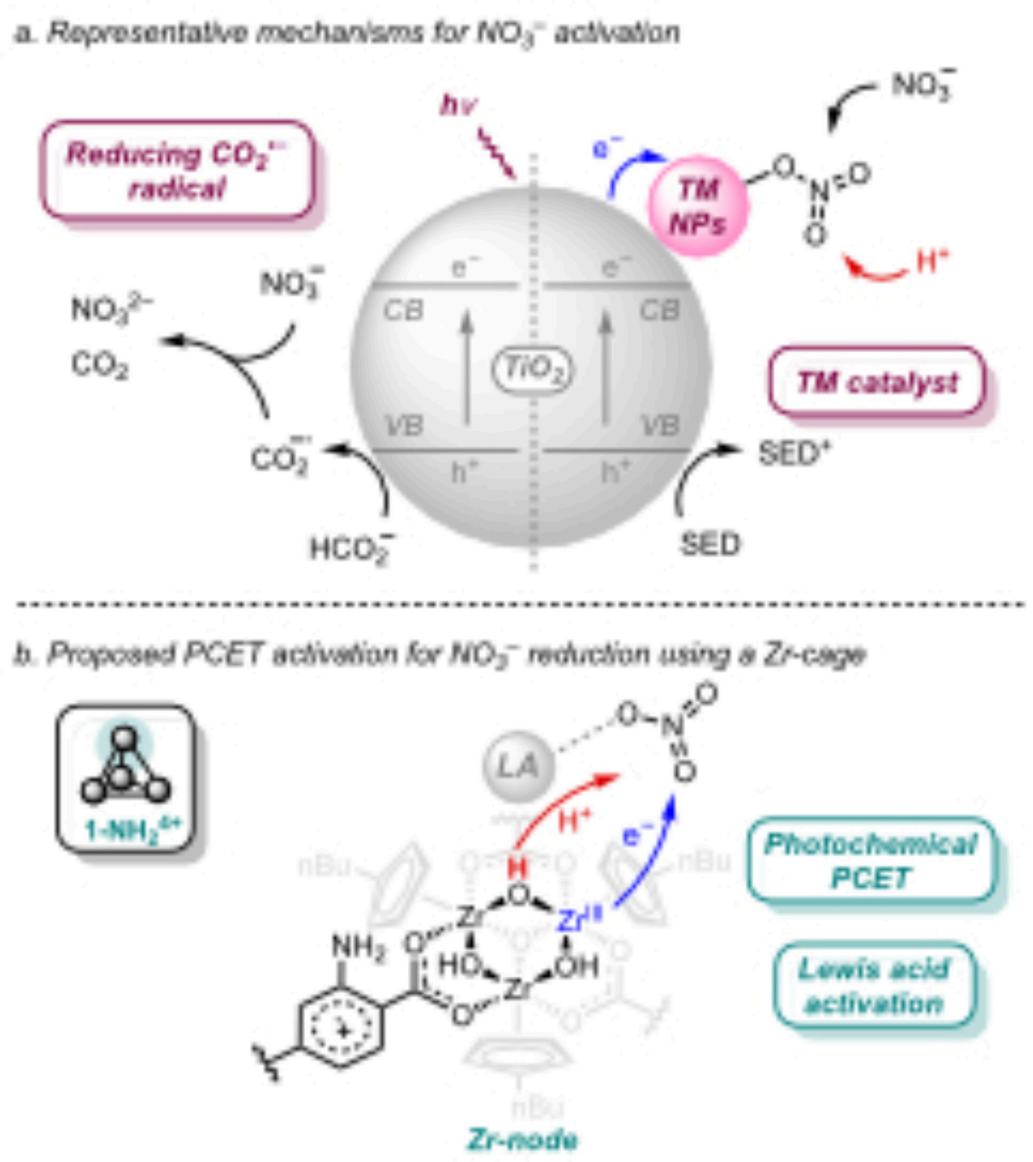
(a) Representative approaches for photocatalytic NO_3_^−^ reduction based on the activity of TiO_2_ semiconductor materials and either the generation of highly reducing radicals from the sacrificial electron donor or the use of a transition metal catalyst. (b) Photocatalytic system studied in this work based on a Zr‐cage (**1‐NH_2_** ^**4+**^, [((^nBu^CpZr)_3_(OH)_3_O)_4_(2‐aminoterephtalate)_6_]Cl_4_; ^nBu^Cp=n‐butylcyclopentadienyl) that promotes PCET to activate NO_3_^−^. LA is Lewis acid.

Using light to power the reduction of NO_3_^−^ is an attractive approach towards exploiting renewable energy and has motivated the growing development of photocatalyst systems, largely focused on semiconductors such as TiO_2_ (Figure 1a).[^16^, ^18^, ^19^] However, their limited reduction potentials hampers the one‐electron activation of NO_3_^−^. Strategies to facilitate the electron transfer and bond breaking steps include using strongly reducing radicals, generated from sacrificial electron donors,[^20^, ^21^] or d‐block metals (e. g., Pt, Pd, Ag, Cu; Figure 1a).[^22^, ^23^, ^24^, ^25^, ^26^, ^27^] Despite important progress, understanding the mechanism and controlling the selectivity of these systems are still ongoing tasks.

Nature has developed a diverse toolbox to activate inert molecules including low energy, proton‐coupled electron transfer (PCET) mechanisms and/or the interplay of redox‐inactive Lewis acids to modulate the redox reactivity. These factors have in fact been proposed in natural enzymes for nitrate and nitrite reduction, although they remain underexplored in synthetic systems.[^28^, ^29^, ^30^] Motivated by these ideas, here we demonstrate that a photoactive Zr‐cage (**1‐NH_2_** ^**4+**^, Figure 1b)^[31]^ can mediate the reduction of NO_3_^−^ to NH_3_ through a distinct activation mechanism that integrates the effect of PCET and a Lewis acid. In addition, we show the impact of competing NO_2_^−^ intermediate oxidation in the NH_3_ yield and combine these findings with the intrinsic activity of Ag as a d‐block metal co‐catalyst to favor the limiting NO_3_^−^ to NO_2_^−^ reduction step.

## Results and Discussion

### Photochemical Reduction of NO_3_^−^ via PCET

**Scheme 1:**
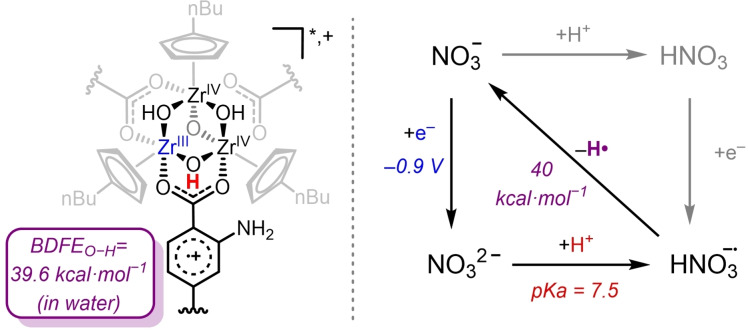
Representation of a node from (**1‐NH_2_** ^**4+**^ )* and the square scheme for the reduction of NO_3_^−^. Potentials (vs NHE) and p*K*_a_ in aqueous solution.

We recently reported on the photocatalytic PCET reactivity of a Zr cage (**1‐NH_2_** ^**4+**^, [((^nBu^CpZr)_3_(OH)_3_O)_4_(2‐aminoterephtalate)_6_]Cl_4_; ^nBu^Cp=n‐butylcyclopentadienyl; see Figure 1b) for the reduction of organic compounds. This cage is formed by four (^nBu^CpZr)_3_(OH)_3_O nodes linked by a total of six 2‐aminoterephtalate ligands. The coordinatively saturated metal nodes prevent coordination of the substrates and thus, **1‐NH_2_** ^**4+**^ acts as an outer sphere H^+^/e^−^ transfer reagent. Upon absorbing blue light (λ < 460 nm), **1‐NH_2_** ^**4+**^ generates an excited state with a reduction potential of −1.8 V vs Fc^+/0^ in MeOH (−0.75 V vs NHE in 1 : 1 H_2_O/MeOH, see SI) associated with the (**1‐NH_2_** ^**4+**^)^*^/**1‐NH_2_** ^**5+**^ couple.^[31]^ An electron transfer from (**1‐NH_2_** ^**4+**^)^*^ to reduce NO_3_^−^ to NO_3_^2−^ (*E°* = −0.90 V vs NHE) is thus thermodynamically uphill.^[17]^ However, analysis of the bond dissociation free energy (BDFE) reveals an alternative reductive pathway. The BDFE_O−H_ in H_2_O is calculated to be ~39.6 kcal⋅mol^−1^ for (**1‐NH_2_** ^**4+**^)^*^ (section S6) and 40.0 kcal⋅mol^−1^ for the HNO_3_^−^⋅ radical anion intermediate (Scheme 1). In both cases, the Bordwell equation (Eq. 1) was used, with a C_G_ in H_2_O of 52.8 kcal⋅mol^−1^ and a p*K*_a_ of 7.5 and 3.4 for HNO_3_^−^⋅ and **1‐NH_2_** ^**4+**^.[^17^, ^32^] This comparison suggests the potential viability of a photochemical PCET from (**1‐NH_2_** ^**4+**^)^*^ to activate NO_3_^−^.

(1) $$BDFE=1.37\cdot pK_{a}+23.06\cdot E^{o}+C_{G}$$

**Figure 2:**
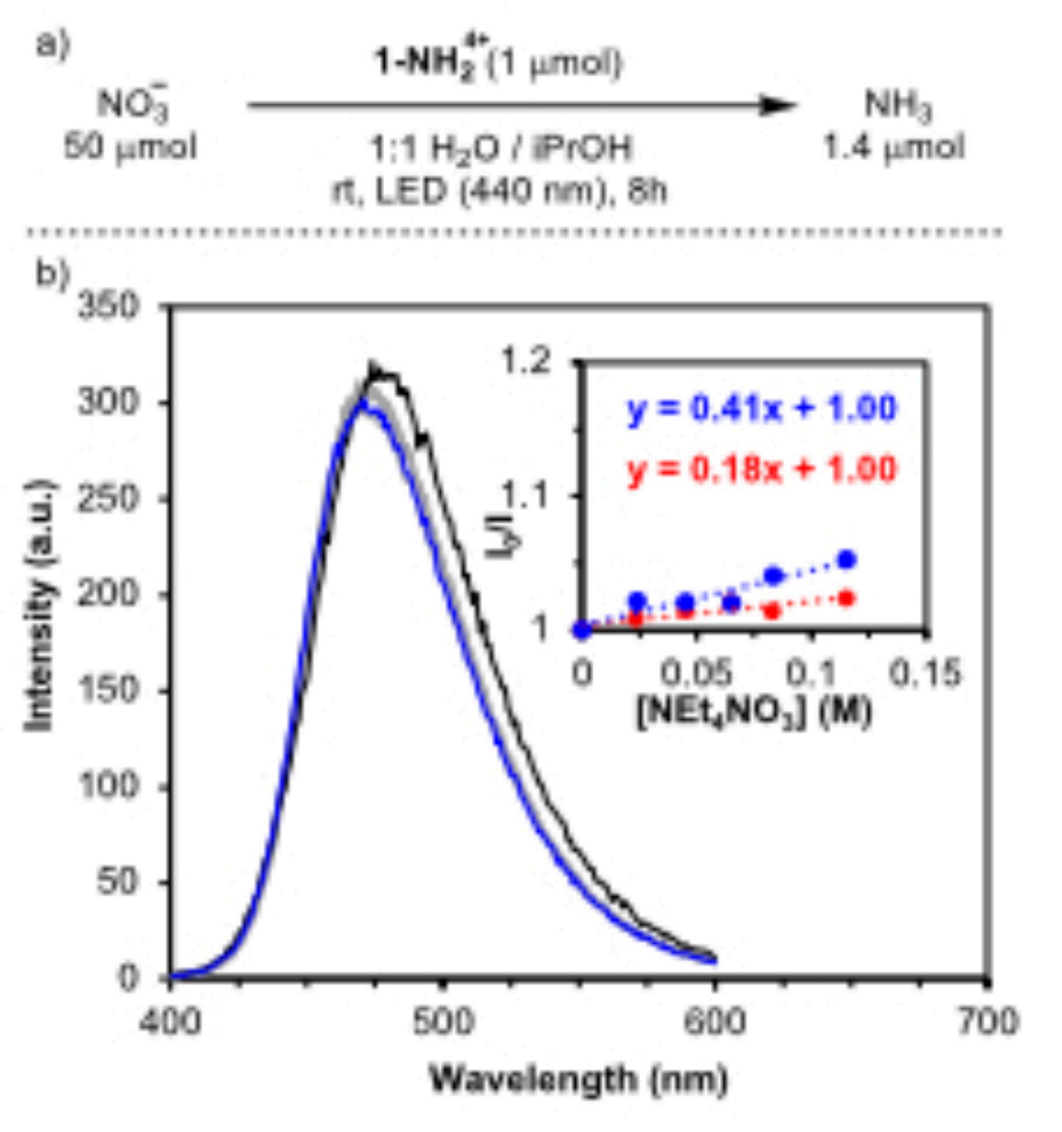
(a) Photochemical reduction of NO_3_^−^ to NH_3_ using **1‐NH_2_** ^**4+**^ under 440 nm irradiation in 1 : 1 H_2_O/iPrOH. (b) Fluorescence of **1‐NH_2_** ^**4+**^ upon addition of [NEt_4_][NO_3_]. The inset shows the Stern–Volmer plots for the quenching in MeOH with **1‐NH_2_** ^**4+**^ (blue) or in MeOD with the deuterated cage (red).

In fact, irradiation with a 440 nm LED lamp of a 1 : 1 H_2_O/iPrOH solution containing **1‐NH_2_** ^**4+**^ (1 μmol) and [NEt_4_][NO_3_] (50 μmol) resulted in the formation of 1.4±0.2 μmol of NH_3_ after 8 h as determined by NMR quantification including a ^15^N‐labelled experiment (Figure 2a, S44 and S56). iPrOH was employed as a sacrificial electron donor (SED) to enable recycling of **1‐NH_2_** ^**4+**^ as previously demonstrated.^[31]^ No NH_3_ was detected in the absence of **1‐NH_2_** ^**4+**^ (Figure S39). In addition, no gaseous nitrogen‐based compounds such as N_2_ or NO_x_ were detected via gas chromatography coupled to mass spectrometry. These results demonstrate the photochemical formation of NH_3_ from NO_3_^−^ using **1‐NH_2_** ^**4+**^ under blue light irradiation.

We turned to fluorescence quenching and Stern‐Volmer analysis to obtain further insights into the mechanism (Figure 1b). Upon adding increasing concentrations of [NEt_4_][NO_3_] to a MeOH solution of **1‐NH_2_** ^**4+**^, a slight decrease of the emission intensity was observed in agreement with the previous photochemical reactivity. However, as expected from the limited NH_3_ yield, the calculated Stern–Volmer constant (K_SV_) remains low (0.41 M^−1^). Performing the fluorescence quenching with the deuterated Zr cage in MeOD supports a photochemical PCET mechanism by observation of a kinetic isotope effect (KIE) of 2.3. In fact, using excess base (triethylamine, TEA) turned off the photochemical quenching due to deprotonation of the Zr‐nodes as previously reported (Figure S21), disfavoring an initial electron transfer.

The lack of changes in the UV‐vis spectrum of **1‐NH_2_** ^**4+**^ upon addition of NO_3_^−^ is consistent with an outer sphere H^+^/e^−^ transfer (Figure S3). Although the efficiency remains low, the fact that **1‐NH_2_** ^**4+**^ can mediate this 8 e^−^/9 H^+^ reaction without a late transition metal catalyst such as Pt, Pd or Ag, is remarkable. In fact, this involves a turnover number (TON) ~11 for this PCET mediator, assuming that the 8 e^−^ required are provided by **1‐NH_2_** ^**4+**^. In addition, the photochemical PCET step provides a distinct NO_3_^−^ activation mechanism that contrasts with previous photocatalytic approaches based on redox‐active transition metals or strongly reducing radical intermediates.^[19]^

### Integrating Lewis Acid for Enhanced PCET

**Figure 3:**
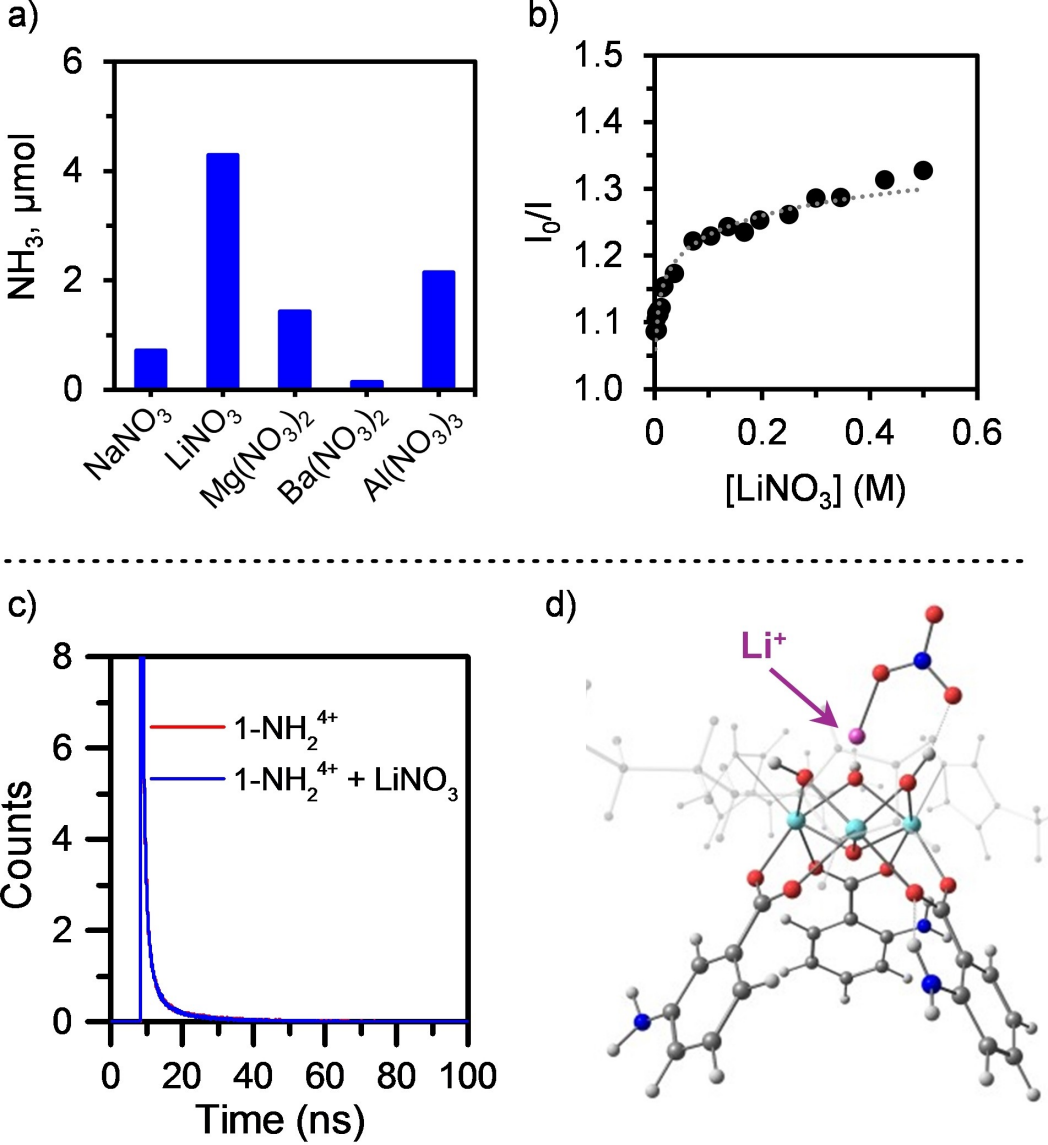
(a) Photochemical production of NH_3_ using **1‐NH_2_** ^**4+**^ under LED irradiation (440 nm) in 1 : 1 H_2_O/iPrOH solution containing different NO_3_^−^ sources. (b) Stern–Volmer plot of a fluorescence quenching experiment in MeOH with **1‐NH_2_** ^**4+**^ and increasing concentrations of LiNO_3_. (c) Lifetime measurements of **1‐NH_2_** ^**4+**^ with and without LiNO_3_ under excitation at λ_exc_=375 nm. (d) DFT optimized structures of a **1‐NH_2_** ^**4+**^ model showing the interactions with a Li^+^ and NO_3_^−^ ions. Color code: Zr in cyan, O in red, C in gray, N in blue, H in white, Li in pink. ^nBu^Cp^−^ ligands are transparent for clarity.

Lewis acids have previously shown to activate nitrite (NO_2_^−^) anions and to facilitate the electron transfer.^[33]^ In addition, they can also coordinate and activate NO_3_^−^ towards oxygen atom transfer to a Mo catalyst.^[34]^ We thought that addition of a Lewis acid to the reaction media could similarly enhance the rate of photochemical PCET. To interrogate this idea, we explored other NO_3_^−^ sources containing different cations such as Na^+^, Li^+^, Mg^2+^, Ba^2+^, and Al^3+^ (Figure 3a). While most of these salts led to NH_3_ yields between 0.2 and 2.1 μmol, similar to previous results with [NEt_4_][NO_3_], the presence of Li^+^ resulted in a significant enhancement generating 4.3±0.4 μmol. This value involves a TON of 34 based on PCET events. In addition, no other gaseous products were detected after a catalytic run (Figure S52).

To explore the unique influence of Li^+^, we turned again to Stern–Volmer analysis that showed the expected quenching of the cage′s fluorescence by LiNO_3_ at higher rates than with [NEt_4_][NO_3_] (Figure 3b). In addition, the I_0_/I vs [LiNO_3_] plot reveals a striking downward deviation that is typically associated with a heterogeneous population of fluorophores with different Stern–Volmer constants.^[35]^ The heterogeneity promoted by Li^+^ could be explained by an interaction with the cage that results in the distinction of emitting sites. We thus assessed the data using the Lehrer equation (Eq. 2) where I and I_0_ are the emission intensity in the presence and absence of quencher respectively, [Q] is the quencher concentration, K_SV_ is the Stern–Volmer constant, and f is the fraction of accessible fluorophores.^[36]^ From the linear fit between I_0_/(I_0_‐I) and 1/[LiNO_3_], we calculate a K_SV_ of 0.54 M^−1^ and a f value of 0.21 (Figure S26), the latter suggesting that a 21 % of the emitting sites in the cage are responsible for the faster quenching rate. As an approximation, the K_SV_ obtained using the traditional Stern–Volmer equation in the plateau region of Figure 3b is 0.29 M^−1^, similar to the value obtained with [NEt_4_][NO_3_], while the K_SV_ of the initial linear region is 6 M^−1^ consistent with a faster PCET promoted by Li^+^‐incorporated sites (Figure S26.

(2) $$\frac{I_{0}}{\left(I_{0}-I\right)}=\;\;\frac{1}{f}+\frac{1}{fK_{SV}\left[Q\right]}$$

To prove the previous point, we performed Li^+^ absorption experiments in an aqueous suspension of **1‐NH_2_** ^**4+**^ that revealed its capability to capture around 1.1 equiv. of Li^+^ (see SI). We speculate that higher Li^+^ loadings are disfavored due to the resulting high charge of the cage. DFT calculations suggest a preferential interaction of the Li^+^ with the OH‐bridges of the node (Figure 3d). Cyclic voltammetry (CV) and differential pulse voltammetry (DPV) of **1‐NH_2_** ^**4+**^ in the presence of LiNTf_2_ (NTf_2_=trifluoromethanesulfonimide) further showcased this interaction as they revealed an appreciable cathodic shift in the waves, associated with the reduction of the nodes (Figure S15). Additional NO_3_^−^ adsorption experiments showed that **1‐NH_2_** ^**4+**^ can capture ~2 more equiv. of NO_3_^−^ in the presence of Li^+^ as compared to NEt_4_^+^, evidencing its role in the pre‐association of NO_3_^−^ with the cage. Accordingly, the lifetime of **(1‐NH_2_** ^**4+**^ )^*^ after addition of LiNO_3_ remains essentially unaltered as measured by time correlated single photon counting (TCSPC) due to the expected static nature of the rapid quenching (Figure 3c).

These results collectively support the generation of two distinct sites within the Zr‐cage upon interaction with Li^+^ featuring different reactivity and resulting in the negative deviation of the Stern–Volmer plot. We reasoned that these interactions are disfavored in the case of di‐ or tri‐ cationic Lewis acids due to the higher coulombic repulsion, thus preventing the pre‐association and activation of the NO_3_^−^ towards PCET.

### Competitive Photo‐oxidation Of NO_2_^−^

**Figure 4:**
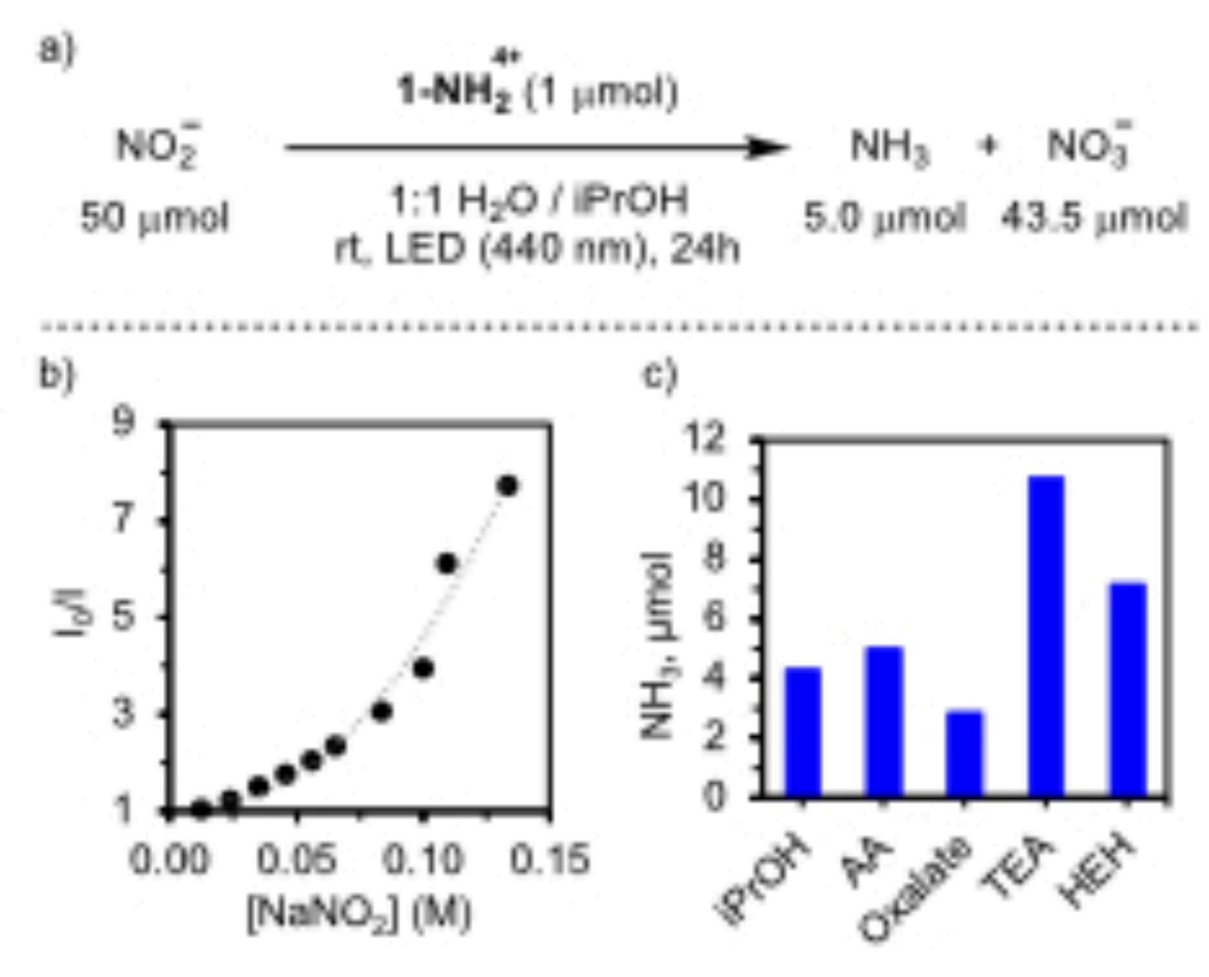
(a) Photochemical dismutation of NO_2_^−^ to NH_3_ and NO_3_^−^ using **1‐NH_2_** ^**4+**^ under 440 nm irradiation in 1 : 1 H_2_O/iPrOH. (b) Stern–Volmer plot from the fluorescence quenching in MeOH under increasing concentrations of NaNO_2_. (c) Photochemical LiNO_3_ reduction using different SEDs in 1 : 1 H_2_O/iPrOH.

After the initial PCET, NO_2_^−^ is a likely intermediate upon subsequent H^+^/e^−^ transfer and H_2_O release. We thus proved that **1‐NH_2_** ^**4+**^ can catalyze the reduction of NO_2_^−^. A photocatalytic run in the presence of NaNO_2_ under similar conditions (1 : 1 H_2_O/iPrOH) resulted in the formation of 5.0 μmol of NH_3_ (Figure 4a). The higher yields even in the absence of Li^+^ reflect the larger thermodynamic driving force compared to the reduction of NO_3_^−^. A fluorescence quenching experiment shows a significant decrease in the emission intensity with increasing [NO_2_^−^], consistent with the more favorable reactivity (Figure S35). In addition, the Stern–Volmer plot follows a distinct trend marked by an upward deviation (Figure 4b), which is usually associated with the presence of both dynamic and static quenching. UV‐vis experiments reveal significant changes in the spectrum of **1‐NH_2_** ^**4+**^ upon addition of NO_2_^−^ that support an interaction responsible for the static contribution of the quenching (Figure S4). Based on the similarities between the p*K*_a_ of NO_2_^−^ (3.1)^[37]^ and **1‐NH_2_** ^**4+**^ (3.4) in water, this could be tentatively assigned to H‐bonding.

However, besides NH_3_, we also detected 43.6 μmol of NO_3_^−^ after the photocatalytic experiment, proving the impact of NO_2_^−^ oxidation in the overall efficiency. The redox potential for the NO_2_⋅/NO_2_^−^ oxidation is around 1.0 V vs NHE,^[38]^ accessible by the oxidized **1‐NH_2_** ^**5+**^ generated after PCET; the latter features a reduction potential *E°*(**1‐NH_2_** ^**5+**^/**1‐NH_2_** ^**4+**^) of 1.8 V vs NHE according to the CV (Figure S14). We disfavored an excited state oxidation of NO_2_^−^ due to the demonstrated lack of reactivity between (**1‐NH_2_** ^**4+**^)* and TEA, which features an more favorable oxidation potential.^[31]^

These results prompted us to evaluate the role of the SED to outcompete the oxidation of NO_2_^−^ and maximize the NH_3_ production (Figure 4c). Previously, the study of the SED has been mainly limited to reduce charge recombination or to generate reducing radical intermediates.^[19]^ We explored the use of SEDs with more thermodynamically accessible oxidations than iPrOH (Figure 4c). The use of Hantzsch ester (HEH, *E°* HEH⋅^+^/HEH ~1.2 V vs NHE)^[39]^ and triethylamine (TEA, *E°* TEA⋅^+^/TEA = 0.6 V vs NHE),^[40]^ commonly employed in photocatalysis, resulted in a significant increase of the yield up to 7.1 and 10.7 μmol of NH_3_. The impact of ascorbic acid (AA, *E°* AA⋅^+^/AA = 0.5 V vs NHE)^[40]^ was more limited, providing 5.0 μmol of NH_3_. However, the use of sodium oxalate (*E°* C_2_O_4_⋅^−^/C_2_O_4_^2−^ ~0.3 V vs NHE)^[40]^ resulted in the lowest amounts of NH_3_ even considering the potential activation of NO_3_^−^ by *in situ* generated CO_2_⋅^−^ radicals.

Consistent with our previous findings, we also found that MeCN affected the photocatalytic PCET reactivity, resulting in 9.9±0.7 and 8.0±0.5 μmol of NH_3_ in 1 : 1 H_2_O/MeCN with HEH and TEA respectively (Figure S65‐S71). Assuming HEH is a 2 e^−^ donor, this result involves a yield with respect to the SED of ~50 % in contrast with the 15 % using iPrOH under similar conditions (Figure S64). In addition, NH_3_ is selectively formed as the reduced product, with 37.5 μmol of NO_3_^−^ accounting for the rest of nitrogen (99 % mass balance). Although some SEDs might not be practical for NO_3_^−^ reduction, these results highlight the importance of outcompeting NO_2_^−^ oxidation.

### Co‐Catalytic System

Previous findings demonstrated a photocatalytic PCET pathway to the activation of NO_3_^−^ as well as the key impact of Lewis acids and the SED to enhance the NH_3_ yields. We then set out to combine these aspects with d‐block metal catalyzed NO_3_^−^ reduction. We were especially interested in incorporating Ag as a co‐catalyst due to its demonstrated role in mediating the NO_3_^−^ to NO_2_^−^ reduction, an important bottleneck of the overall process.^[23]^

**Figure 5:**
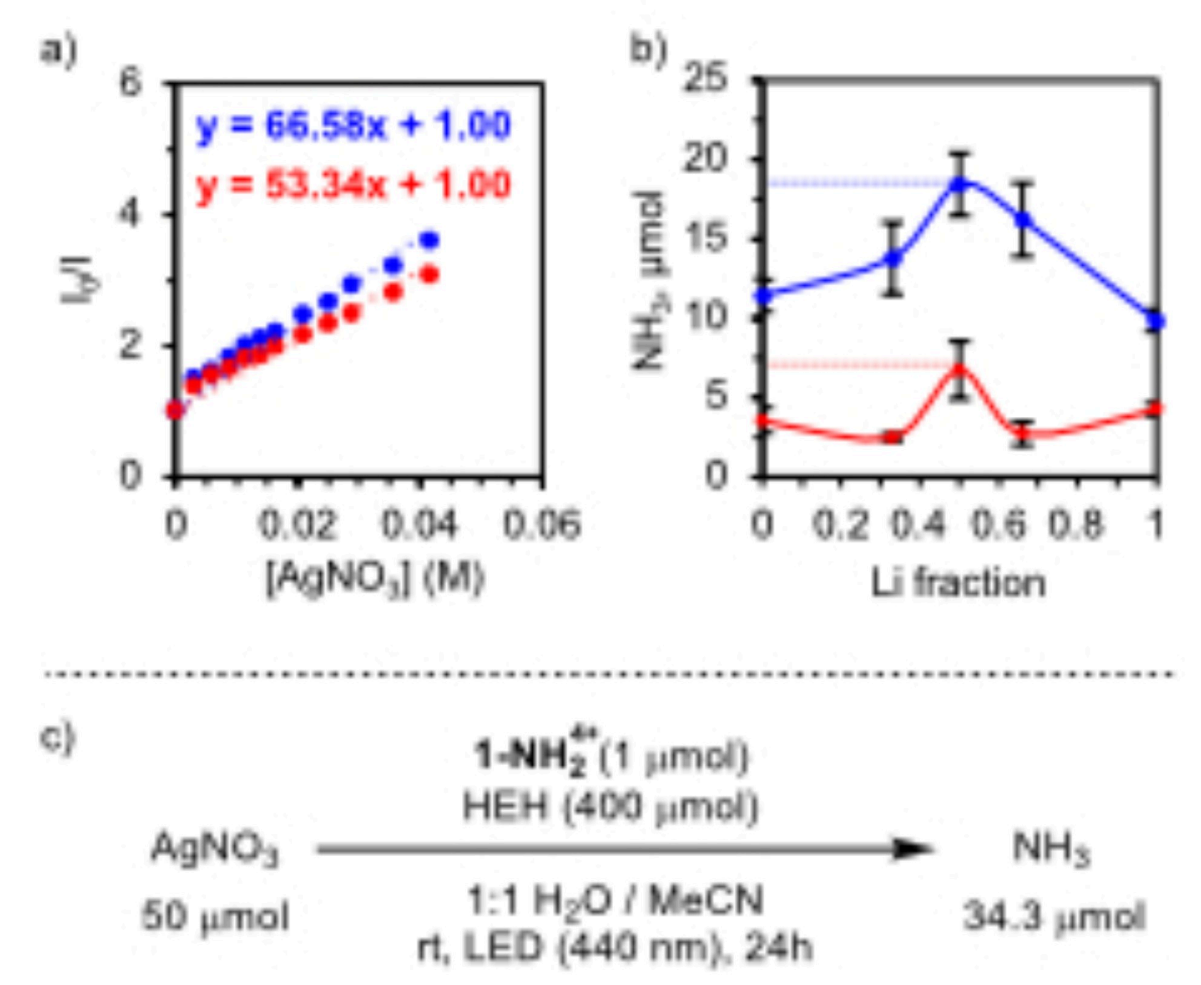
(a) Stern–Volmer plot from the fluorescence quenching in MeOH with **1‐NH_2_** ^**4+**^ (blue) or in MeOD with the deuterated cage (red). (b) Photocatalytic NH_3_ production using different ratios of AgNO_3_/LiNO_3_ under 440 nm irradiation in 1 : 1 H_2_O/iPrOH (red trace) and 1 : 1 H_2_O/MeCN with 100 μmol HEH (blue trace). (c) Photochemical reduction of NO_3_^−^ to NH_3_ using **1‐NH_2_** ^**4+**^ and AgNO_3_ under 440 nm irradiation in 1 : 1 H_2_O/MeCN with 400 μmol HEH.

A photocatalytic run using AgNO_3_ in 1 : 1 H_2_O/iPrOH produces 3.6±0.8 μmol of NH_3_, even in the absence of Li^+^, evidencing the role of the Ag in kinetically facilitating NO_3_^−^ to NO_2_^−^ conversion (Figure S77). Fluorescence experiments exhibit a rapid quenching rate upon addition of AgNO_3_, assigned to a PCET step from (**1‐NH_2_** ^**4+**^)* to the Ag‐NO_3_ complex formed in aqueous solution.^[41]^ The Stern–Volmer plot follows a linear trend in contrast with previous results obtained using LiNO_3_, consistent with the lack of interaction between **1‐NH_2_** ^**4+**^ and Ag^+^ as opposed to Li^+^ (Figure 5a). A KIE of 1.3 is obtained for these reaction conditions, supporting the photochemical PCET nature of the quenching that, based on the calculated K_SV_ (74 M^−1^), takes places at faster rates than with Li^+^. This PCET step gates the NO_3_^−^ reduction catalyzed by Ag^+^ to produce NO_2_^−^ at higher efficiency than with **1‐NH_2_** ^**4+**^ alone. Subsequently, the resulting NO_2_^−^ is further reduced to generate NH_3_ by **1‐NH_2_** ^**4+**^ as previously demonstrated. While we cannot rule out the role of Ag^+^ as a redox‐inactive Lewis acid, (**1‐NH_2_** ^**4+**^)* is capable to reduce Ag^+^ in the absence of NO_3_^−^ as inferred from fluorescence quenching experiments (Figure S31), supporting its role as the electron acceptor site in the PCET step.

We further interrogated the potential synergistic effect between Ag^+^ and Li^+^, so we tested different ratios of the corresponding nitrate salts (Figure 5b). The results show that a 1 : 1 combination of AgNO_3_ and LiNO_3_ provides the highest yield with 6.7±1.8 μmol of NH_3_. This optimized ratio suggests a potential combined effect where Li^+^ might serve to activate the Ag‐NO_3_ complex towards PCET. In addition, using different SEDs and solvent parallels the results previously obtained, where HEH maximizes the production of NH_3_ yielding 11.4±1.0 μmol in 1 : 1 H_2_O/MeCN. This involves a yield with respect to the SED of ~47 %, with 33.5 μmol of NO_3_^−^ recovered. While slightly more attenuated, the combined effect of Li^+^ and Ag^+^ is also evidenced under these conditions, with a maximum conversion of 18.4±2.0 μmol of NH_3_ at a 1 : 1 LiNO_3_/AgNO_3_ ratio and a NH_3_/SED yield of 73 %. Increasing the concentration of the SED (400 μmol) with AgNO_3_ also resulted in higher production of NH_3_ with 34.3 μmol of NH_3_ but at the cost of the electron efficiency that decreases to 35.7 % (Figure 5c).

## Conclusions

The photocatalytic reduction of NO_3_^−^ to NH_3_ is a highly desired reaction for a circular economy model in the synthesis of nitrogen compounds. However, the redox activation of this highly inert molecule is a major challenge for a viable synthetic route. Here we show photochemical PCET as a distinct and competent mechanism to activate NO_3_^−^ using a molecular Zr coordination cage, demonstrating the catalytic formation of NH_3_. The incorporation of Li^+^ as a Lewis acid has a unique impact generating highly active sites in the Zr cage where the NO_3_^−^ is coordinated and activated. We also show that the oxidation of NO_2_^−^, an intermediate of the catalytic process, is an important threat to the efficiency and that can be partially outcompeted by the choice of SED. Finally, we merge these concepts with d‐block metal catalyzed NO_3_^−^ reduction using Ag^+^ as co‐catalyst providing selectively NH_3_ at a yield with respect to the SED of up to 73 %. Our results highlight the potential of combining PCET mechanisms and Lewis acid activation, tools that are widely employed by nature, for the selective reduction of NO_3_^−^ to NH_3_.

## Supporting Information

The authors have cited additional references within the Supporting Information.[^31^, ^42^, ^43^, ^44^, ^45^, ^46^, ^47^, ^48^, ^49^]

## Conflict of Interests

The authors declare no conflict of interest.

1

## Supporting information

As a service to our authors and readers, this journal provides supporting information supplied by the authors. Such materials are peer reviewed and may be re‐organized for online delivery, but are not copy‐edited or typeset. Technical support issues arising from supporting information (other than missing files) should be addressed to the authors.

Supporting Information

## Data Availability

The data that support the findings of this study are available in the supplementary material of this article.
