# Supplementary material for: Photocatalytic Nitrate Reduction to Ammonia via Zr‐Mediated Proton‐Coupled Electron Transfer
Source: ChemSusChem. 2025 Apr 4;18(11):e202402630. doi: 10.1002/cssc.202402630 (PMC12131677; doi:10.1002/cssc.202402630)
Supplement: Supplementary file 1 — Supporting Information [file CSSC-18-e202402630-s001.pdf]

ChemSusChem

Supporting Information

Photocatalytic Nitrate Reduction to Ammonia via Zr-Mediated Proton-Coupled Electron Transfer

Pedro J. Jabalera-Ortiz, Alvaro M. Rodriguez-Jimenez, and Pablo Garrido-Barros*

Supporting Information for

Photocatalytic Nitrate Reduction to Ammonia via Zr-mediated Proton-Coupled Electron Transfer

Pedro J. Jabalera-Ortiz, Alvaro M. Rodriguez-Jimenez, Pablo Garrido-Barros*

Departamento de Química Inorgánica, Facultad de Ciencias, Universidad de Granada and Unidad de Excelencia en Química (UEQ), Avda. Fuente Nueva s/n, 18071, Granada, Spain. E-mail: pgarridobarros@ugr.es

Table of contents

S.1. General considerations 4

S.1.1. Materials and Reagents 4

S.1.2. Physical and chemical methods of characterization. 4

S.1.3. Computational details 5

S.2. Synthesis and characterization of $1\text{-}NH_2^{4+}$ 6

S.3. Titration ($1\text{-}NH_2^{4+}$) 7

S.4. UV-vis spectroscopy 8

S.4.1. Colorimetric determination of NO_3^- 9

S.4.1.1. NO_3^- absorption experiments 12

S.5. Electrochemistry 13

S.6. Calculation of the BDFE 15

S.7. Lifetime 16

S.8. Stern-Volmer 17

S.8.1. NEt_4NO_3 addition 18

S.8.2. $LiNO_3$ addition 20

S.8.3. $AgNO_3$ addition 23

S.8.4. AgOTf addition 25

S.8.5. H_2O addition 26

S.8.6. $NaNO_2$ addition 27

S.9. Relevant photocatalytic reactions 28

S.9.1. Control reactions 29

S.9.2. Lewis acids 31

S.9.3. ^{15}N 38

S.9.4. Solvents 39

S.9.5. Sacrificial electron donors 40

S.9.6. Reduction of potential intermediates 47

S.9.7. Ag^+ addition 49

S.9.8. Combined Li^+ and Ag^+ catalysis 57

S.10. DFT calculations 72

S.11. References 74

S.1. General considerations

S.1.1. Materials and Reagents

Chemical reagents and solvents were purchased at commercial sources and used without additional purification. Dried solvents were purchased from Sigma Aldrich and sparged with N_2 before their use. Deuterated DMSO-d_6, and MeOD solvents (D, 99.9% with a purity of 99.5%) were purchased from Cambridge Isotope Laboratories, Inc., and used as received. Teflon-coated magnetic stir bars were soaked in concentrated nitric acid for at least 1 h, washed repeatedly with deionized water and acetone, and oven dried.

S.1.2. Physical and chemical methods of characterization.

^{1}H Nuclear Magnetic Resonance Spectroscopy (RMN) data were recorded on a 400 MHz BRUKER Nanobay Avance III HD High-Definition spectrometer and the spectra were internally referenced to solvent signals

UV-vis spectroscopy was performed in a SHUMADZU UV-1800 UV/VIS Scanning Spectrophotometer.

Cyclic voltammetry was carried out on a WaveNow Wireless Potentiostat/Galvanostat, using a one-compartment three-electrode cell, a glassy carbon (GC) disk as the working electrode, a Pt disk as the counter electrode, and an Ag/AgOTf (5 mM) reference electrode. Details for the CVs are noted as they appear. $E_{1/2}$ values for the reversible waves were obtained from the half potential between the oxidative and reductive peaks. All the reported potentials are referenced to the ferrocenium/ferrocene couple ($Fc^{+/0}$), which has been used as an internal standard. The GC disk electrode for cyclic voltammetry was polished using 1, 0.3 and 0.05 μm alumina powder.

Steady-state luminescence was recorded on a Cary Eclipse Fluorescence Spectrometer. Excitation was provided by a 450 W Xe arc lamp, wavelength-selected with a 0.25 m monochromator. Luminescence was collected at 90° with reflective optics. All spectra were corrected for instrument response.

ICP-OES was performed in an ICP-OES PERKIN-ELMER OPTIMA 8300 (dual Vision) spectrometer with a PERKIN-ELMER S10 automatic sampler. A standard solution of LiCl was prepared containing 123 mg/L and was subjected to Li absorption by **1-NH_2^{4+}**, providing a lower Li quantification at the end of the experiment of 111.8 mg/L. The absorption value was converted into equivalent of Li^+ vs **1-NH_2^{4+}** using the molecular weight of each compound.

Mass Spectrometry in gas phase: Gaseous products such as H_2, H2, N_2, NO, and N_2O were quantified using a mass spectrometer HDR-20 R&D.

Ion chromatography: 940 Professional IC Vario

S.1.3. Computational details

All DFT calculations were performed in the Gaussian 09,[1] using the TPSS (meta-GGA)[2] functional with def2-SVP[3,4] on all atoms and SMD[5] implicit solvation modelling water and acetonitrile was used to calculate the thermochemical parameters (for direct comparison with experimental available data). Geometry optimizations were computed in solution without symmetry restrictions. All calculated structures were stationary points as confirmed by single-point vibrational frequency calculations. Free energy corrections were calculated at 298.15 K and 105 Pa pressure, including zero-point energy corrections (ZPE). Unless otherwise mentioned, all reported energy values are free energies in solution under standard state conditions. The energies of Li^+ and NO_3^- complexes were calculatd in water and acetonitrile. The DFT calculations with the optimized xyz coordinates are available in the ioChem-BD database[6] and can be accessed at: http://dx.doi.org/10.19061/iochem-bd-6-432

S.2. Synthesis and characterization of 1-NH_2^{4+}

The synthesis of $[((^{nBu}CpZr)_3(OH)_3O)_4(2\text{-aminoterephtalate})_6]Cl_4$ ([1-NH_2]Cl_4) was carried out according to the procedure stablished by Delgado *et al.* (2022)[7] and physical, chemical characterization and deuteration of the resulting material was done according to Jabalera-Ortiz *et al.* (2024)[8]. Additionally, once synthetized, 1-NH_2^{4+} was activated in order to eliminate residual solvent.

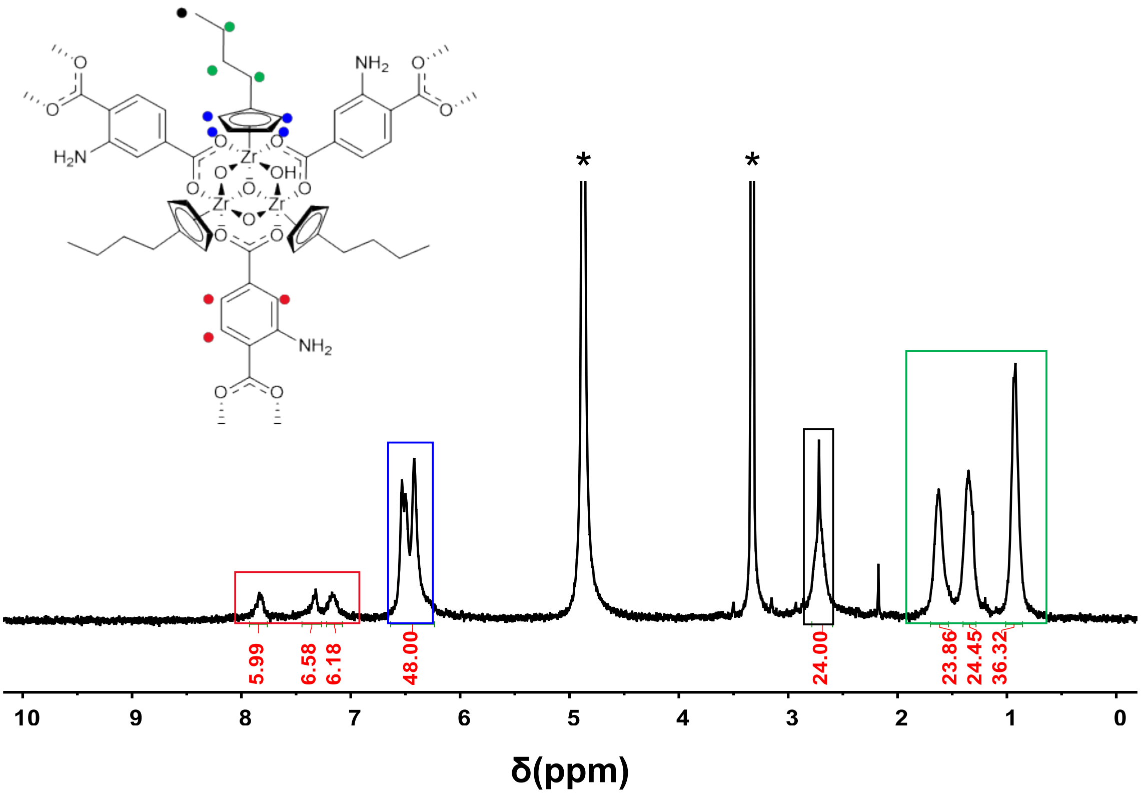

Figure S1. NMR ^1H spectra in methanol-d4 of 1-NH_2^{4+} activated. Peaks marked with black asterisk correspond to methanol-d4 and water.

S.3. Titration (1-NH_2^{4+})

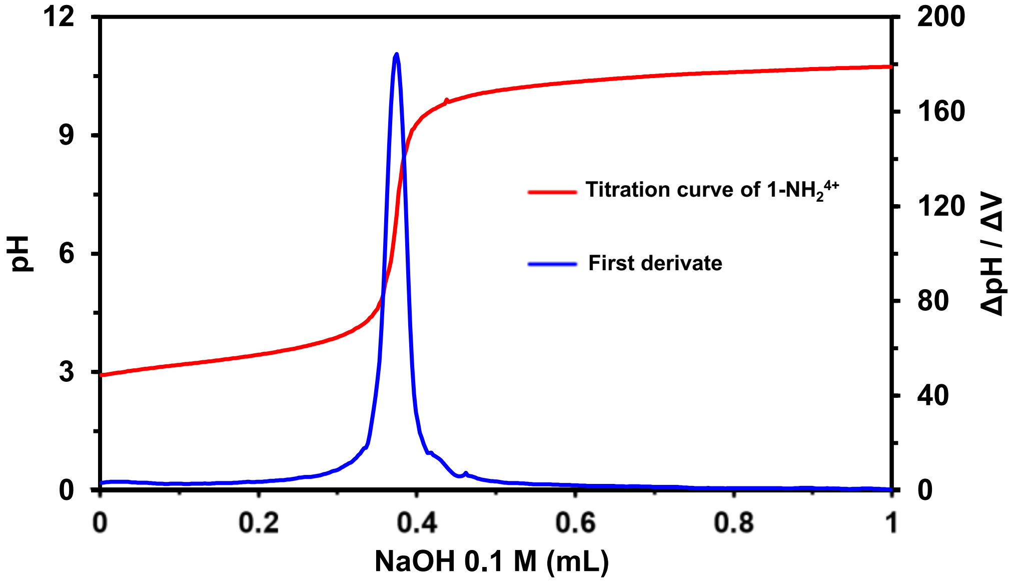

Figure S2. Acid base titration curve of 1-NH_2^{4+} and its first derivate, which was calculated by the relationship between the increment of the pH and the volume of NaOH 0.1 M added. Then, pKa of 3.4 was obtained dividing by half the pH reached in the peak of the first derivate.

S.4. UV-vis spectroscopy

Prior to measure, a stock solution with $5 \cdot 10^{-2}$ mM of 1-NH_2^{4+} was prepared in methanol.

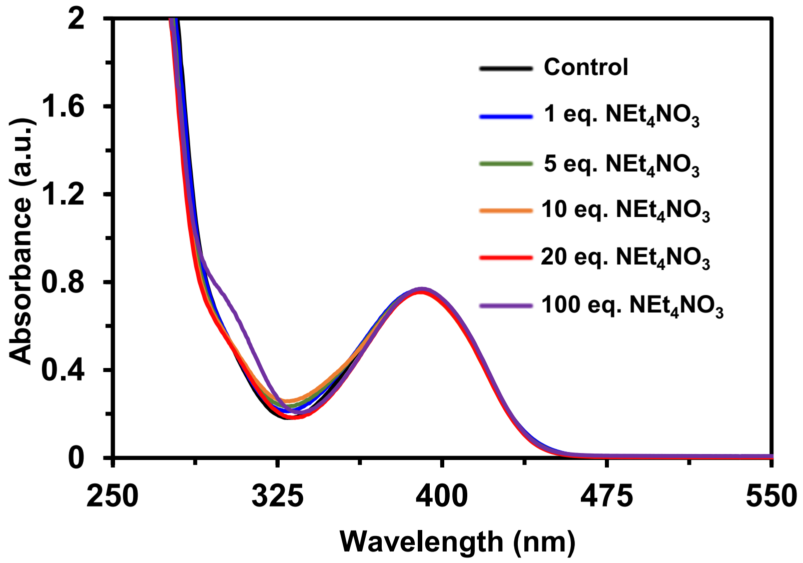

Figure S3. UV-vis absorption spectra of 1-NH_2^{4+} with increasing equivalents of NEt_4NO_3.

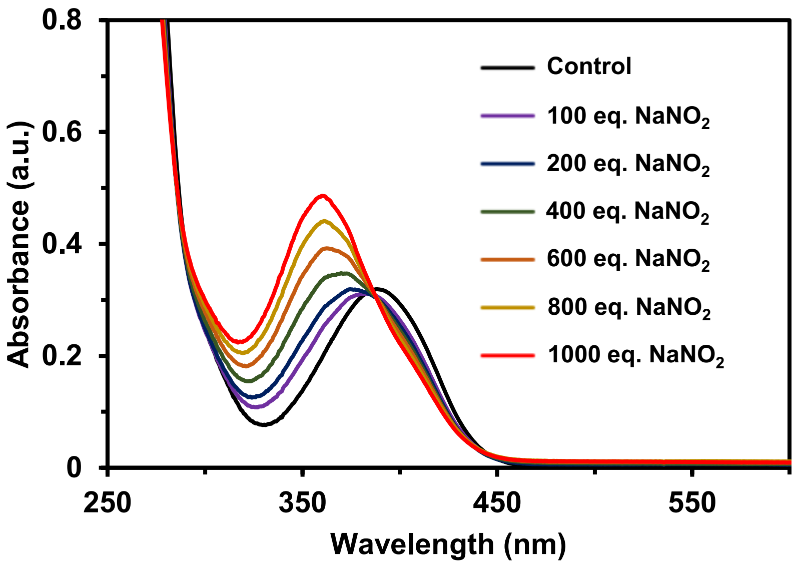

Figure S4. UV-vis absorption spectra of 1-NH_2^{4+} with increasing equivalents of $NaNO_2$.

S.4.1. Colorimetric determination of NO_3^-

Determination of NO_3^- was carried out following the Berthelot and Griess tests as described by Suryanto *et al.* (2021)[9].

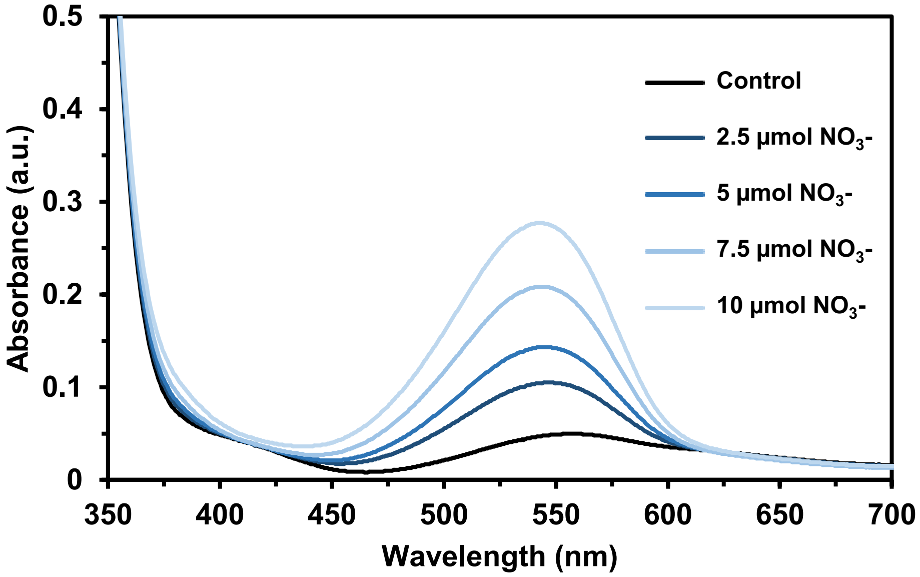

Figure S5. UV-vis of the colorimetric method for quantification of NO_3^-.

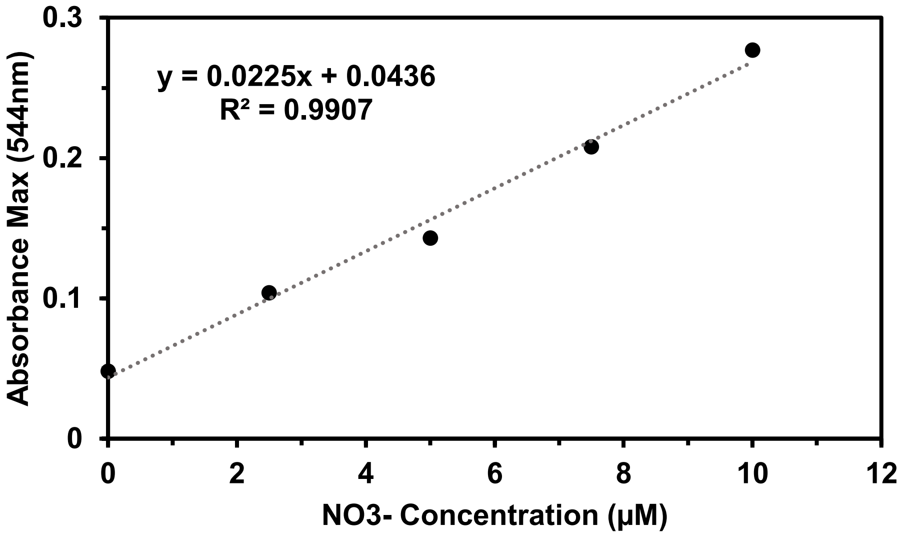

Figure S6. Linear regression for the fitting pattern of NO_3^- calibration

Catalytic run: In a 5 mL vial, substrate (50 µmol) and 1-NH_2^{4+} (1 µmol) were added to 2 mL of solvent, following the general description for the catalytic runs (Section S8). An aliquot of 10 µL was taken after 24 h of reaction.

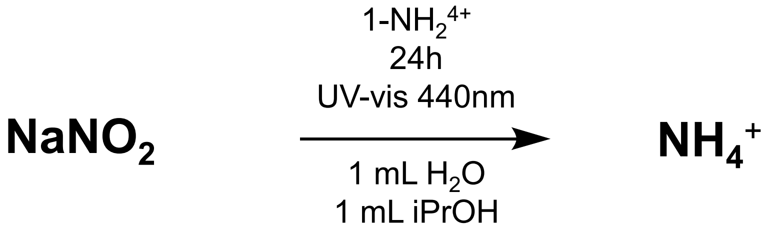

Figure S7. Scheme of the photocatalytic reaction mediated by 1-NH_2^{4+} in H_2O:iPrOH using $NaNO_2$ as the substrate.

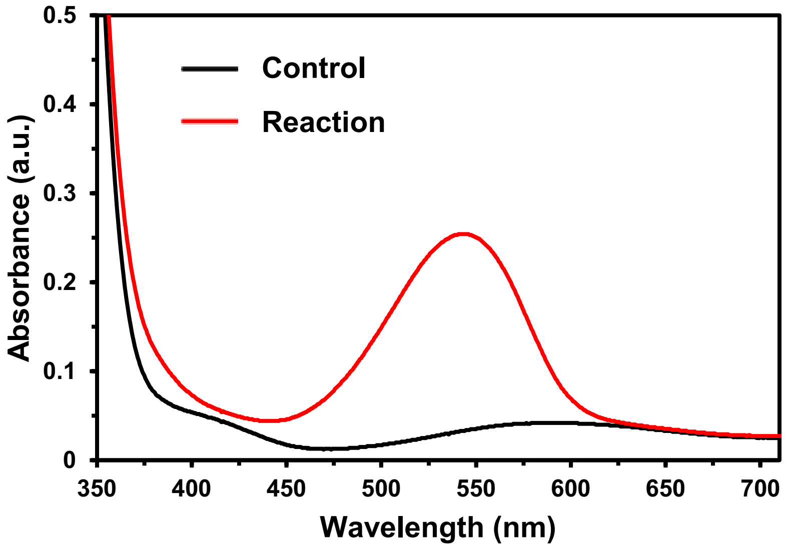

Figure S8. Determination of NO_3^- by UV-vis absorbance of $NaNO_2$ oxidation (red).

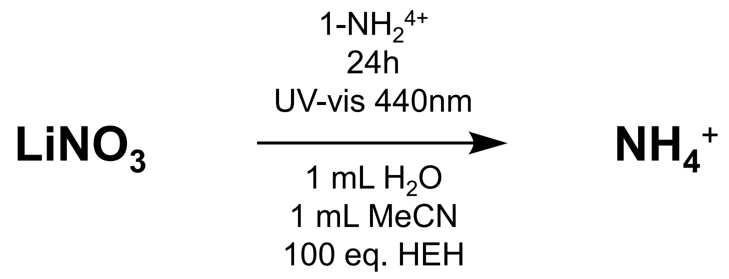

Figure S9. Scheme of the photocatalytic reaction mediated by $1\text{-}NH_2^{4+}$ in H_2O:MeCN using $LiNO_3$ as the substrate and 100 equivalents of HEH as sacrificial electron donor.

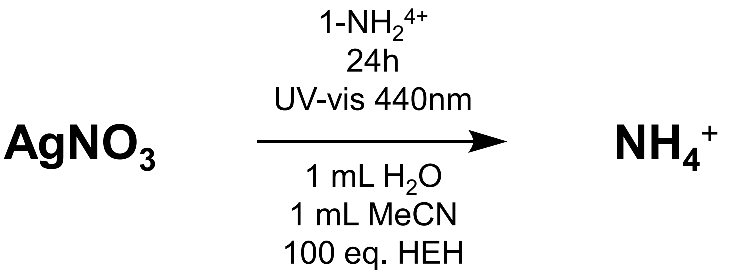

Figure S10. Scheme of the photocatalytic reaction mediated by $1\text{-}NH_2^{4+}$ with 100 equivalents of HEH in H_2O:MeCN using $AgNO_3$ as the substrate.

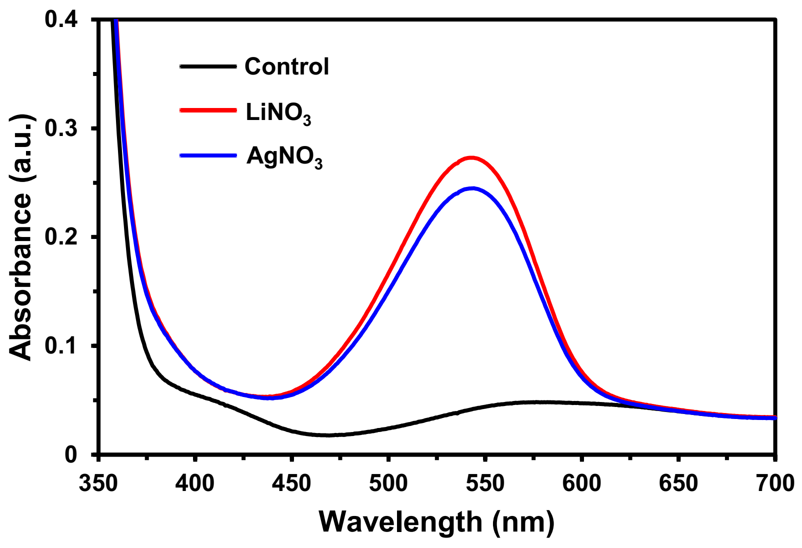

Figure S11. Determination of NO_3^- by UV-vis absorbance of $NaNO_2$ oxidation (red).

S.4.1.1. NO_3^- absorption experiments

In a 5 mL vial, substrate (50 µmol) and 1-NH_2^{4+} (1 µmol) were added to 2 mL of water. A first aliquot of 10 µL was taken right upon addition and the second aliquot after stirring for 24 h.

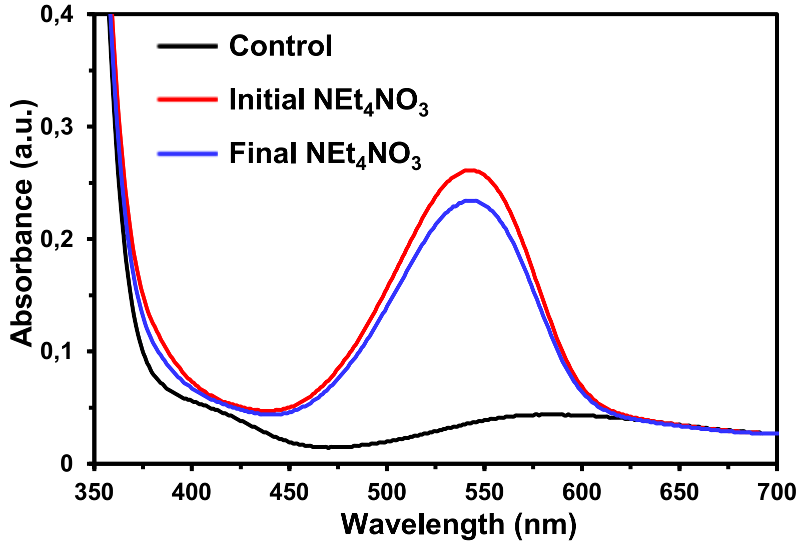

Figure S12. Determination of NO_3^- absorption by UV-vis in the presence of NEt_4NO_3 before (red) and after (blue) absorption with 1-NH_2^{4+} by incubation of 24 h. Absorption in the absence of Li^+ is attributed to anion exchange with Cl^- and/or weak interactions between **1-NH_2^{4+}** and NO_3^- such as H-bonding or coulombic attraction.

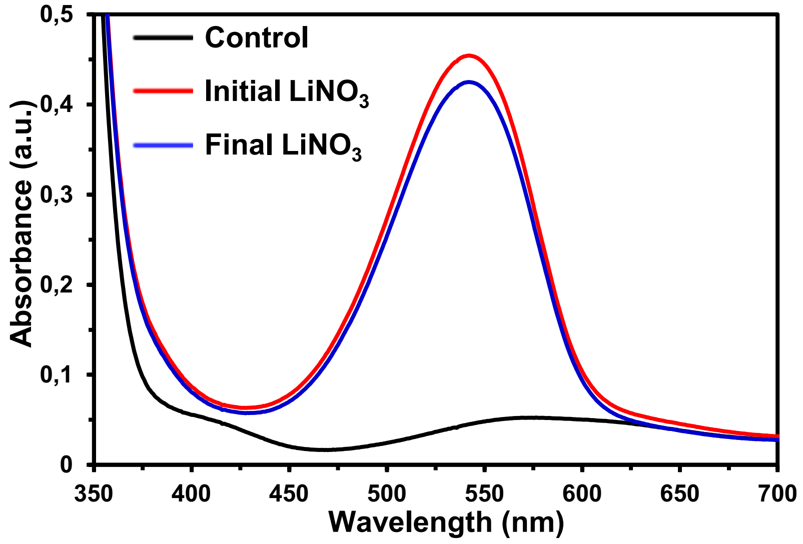

Figure S13. Determination of NO_3^- absorption by UV-vis in the presence of $LiNO_3$ before (red) and after (blue) absorption with 1-NH_2^{4+} by incubation of 24 h.

S.5. Electrochemistry

In a 26 mL glass vial, 12 mg of 1-NH_2^{4+} (0.1M) and 194 mg of tetrabutylammonium hexafluorophosphate ($TBAPF_6$) (0.1 M) or 78 mg $LiNTf_2$ (NTf_2 = trifluoromethanesulfonimide; 0.1 M) were dissolved in 5 mL of anhydrous MeOH or a mixture of H_2O:MeOH. The solution was then put under a constant flow of argon during 15 min before measurement to eliminate the presence of O_2.

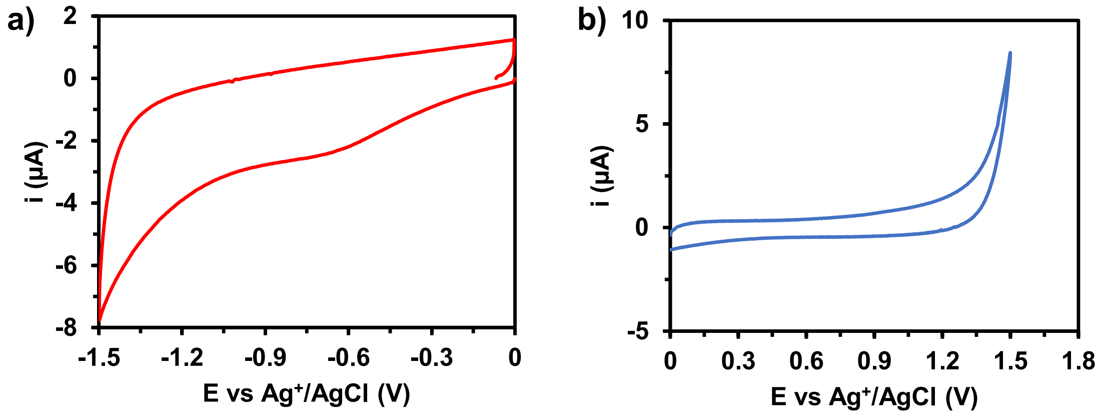

Figure S14. (a) Cathodic and (b) anodic scans of a cyclic voltammetry of 1-NH_2^{4+} in presence of $TBAPF_6$ in a solution of H_2O:MeOH.

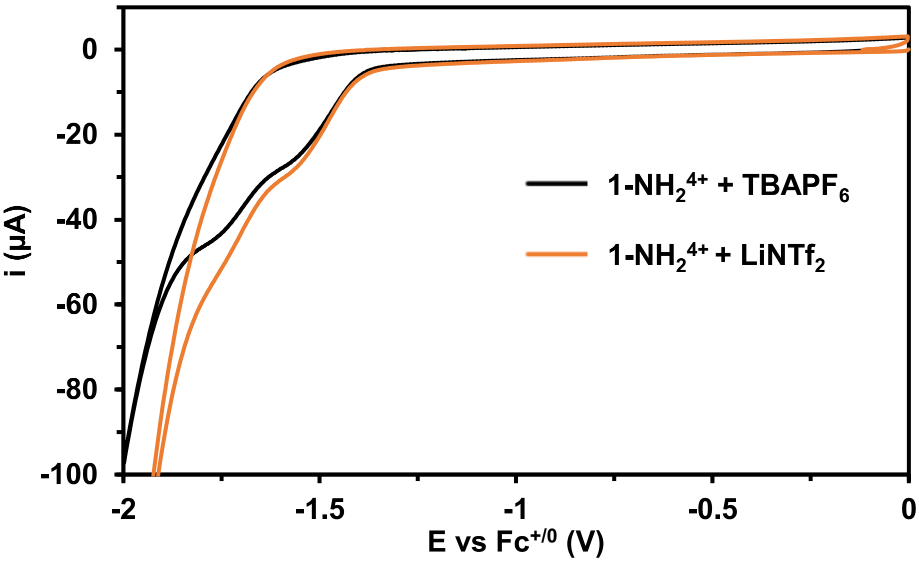

Figure S15. Cyclic voltammetry of 1-NH_2^{4+} in presence of $TBAPF_6$ (black) or $LiNTf_2$ (orange).

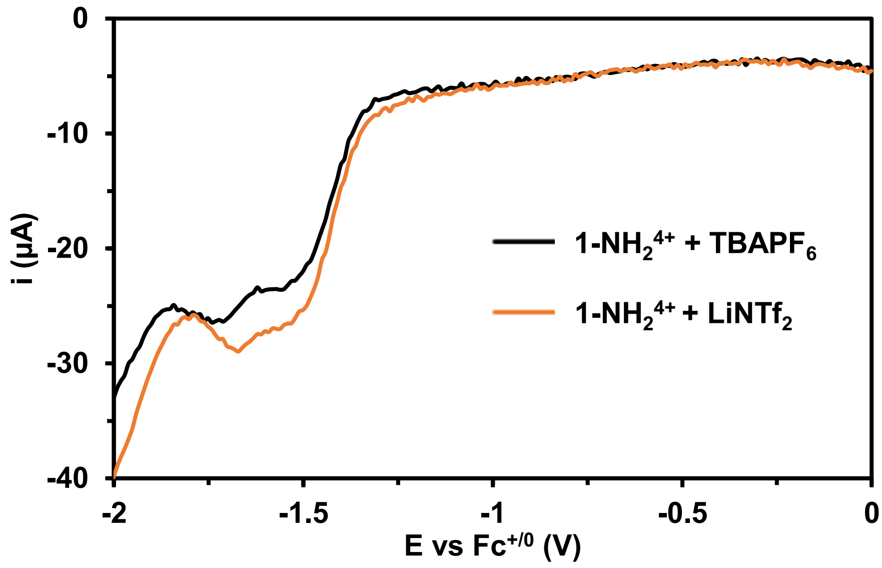

Figure S16. Differential pulse voltammetry of 1-NH_2^{4+} in presence of $TBAPF_6$ (black) or $LiNTf_2$ (orange).

S.6. Calculation of the BDFE

Based on previous characterization of **1-NH$_2$**$^{4+}$ in aqueous solution, the excited state redox potential associated to **1-NH$_2$**$^{4+,*/3+}$ is calculated using Equation S1 and results in a value of around -0.75 V vs NHE consistent with the reduction potential of the Zr nodes. The energy gap between the zeroth vibrational levels of the ground and excited states (E_{00} = 2.8 eV) was estimated to be the same as in MeOH due to the lack of spectral changes upon addition of water.

$$E°(\mathbf{1-NH_2}^{4+,*/3+}) = E°(\mathbf{1-NH_2}^{4+/3+}) + E_{00} \quad (Eq.S1)$$

The effective bond dissociation free energy ($BDFE_{eff}$) of the excited state was calculated the Bordwell equation (Eq. S2) where C_G is the solvent dependent constant (52.8 kcal·mol^{-1} in water), pK_a is 3.4 according to the previous titration experiment and E^0 is the excited redox potential calculated above (-0.75 V vs NHE).

$$BDFE = 1.37 \cdot pK_a + 23.06 \cdot E^0 + C_G \quad (Eq.S2)$$

S.7. Lifetime

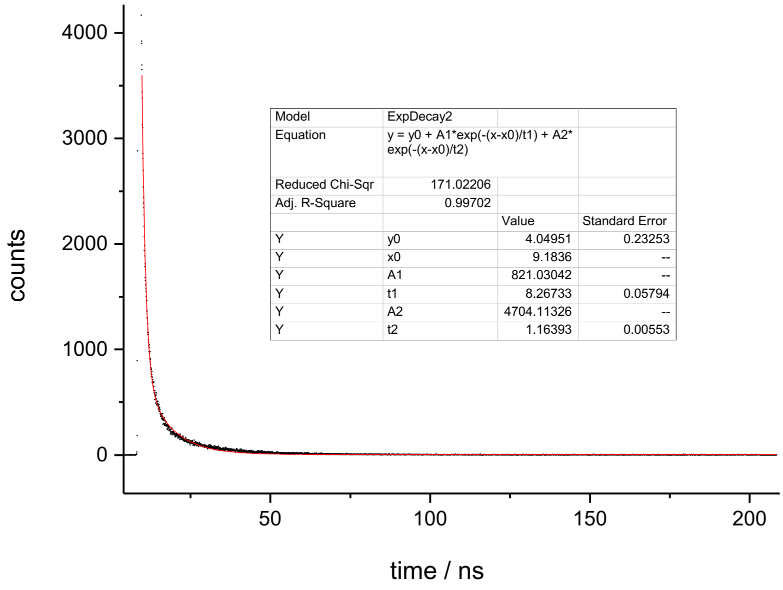

Figure S17. Decay of 1-NH_2^{4+} emission at 480 nm after 375 nm excitation of a 20 µM solution in MeOH at 20 ºC obtained with time correlated single photon counting (TCSPC) technique. Red trace shows the fitting to a biexponential decay.

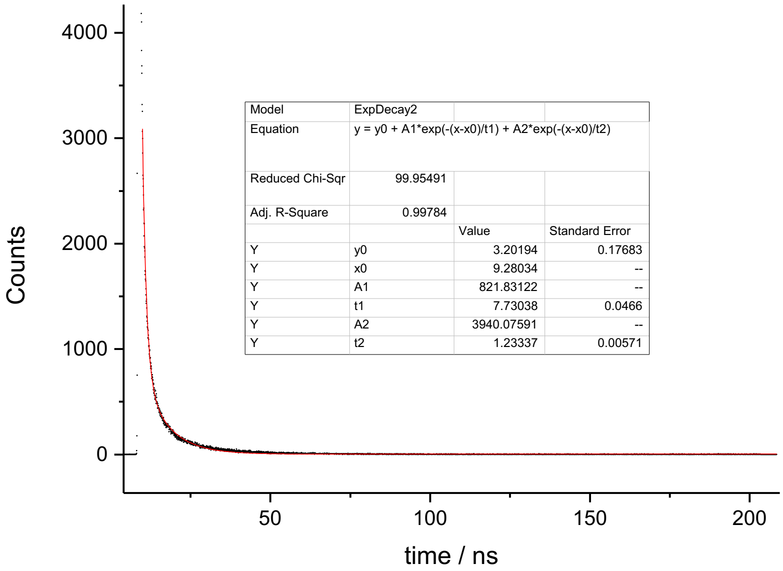

Figure S18. Decay of 1-NH_2^{4+} in the presence of $LiNO_3$ emission at 480 nm after 375 nm excitation of a 20 µM solution in MeOH at 20 ºC obtained with time correlated single photon counting (TCSPC) technique. Red trace shows the fitting to a biexponential decay.

S.8. Stern-Volmer

In an Ar-filled glove box, 2 mg of 1-NH_2^{4+} (0,5 μmol) were dissolved in 4 mL of dry methanol. 2 mL were put in a fluorescence cuvette and the other 2 mL were added equivalents/excess of either NEt_4NO_3, $LiNO_3$, $AgNO_3$, AgOTf (OTf = trifluoromethanesulfonate), H_2O or $NaNO_2$. The cuvette and the solution were covered by a septum and taken out from the glove box in order to measure the fluorescence emission upon addition of the quncher.

Stern-Volmer plots were made by monitoring the relationships between the initial emission intensity without quencher (I_0) and the emission intensity (I) versus the quencher concentration (Q). K_{SV} is the slope of the plot corresponding to the product between the kinetic rate of the quenching process (PCET in our case, k_{PCET}) and the lifetime of the excited state (τ_0). The resulting plot was fitted to a linear regression, from which K_{SV} can be extracted.

$$\frac{I_0}{I} = 1 + K_{SV}[Q] \quad Eq. S1$$

$$K_{SV} = k_{PCET}\tau_0 \quad Eq. S2$$

S.8.1. NEt_4NO_3 addition

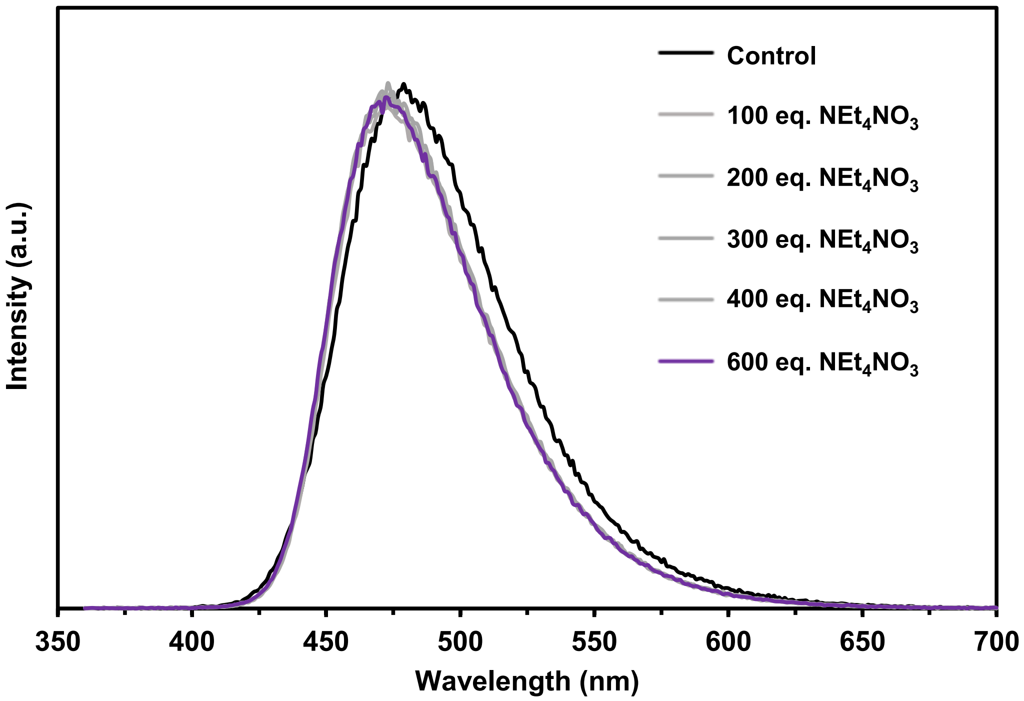

Figure S19. Fluorescence emission spectra of the deuterated 1-NH_2^{4+} (0.2 mM) in **methanol-d4** in the presence of increasing equivalents of NEt_4NO_3. The excitation wavelength was 350 nm.

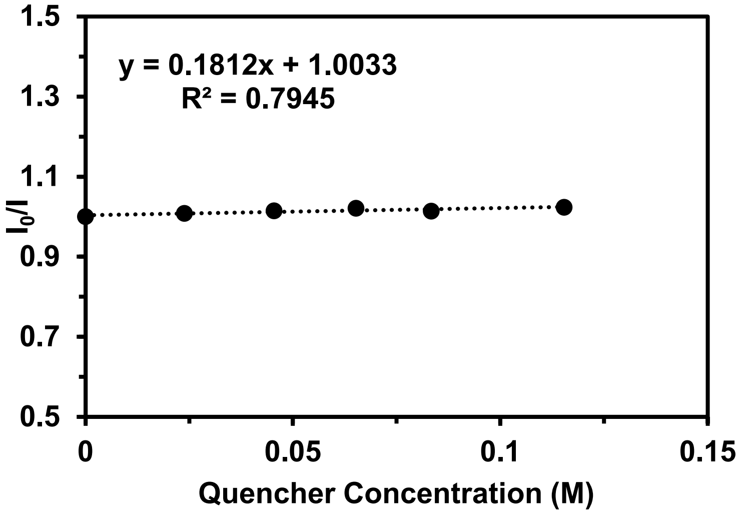

Figure S20. Stern-Volmer plot of I_0/I versus quencher concentration (NEt_4NO_3) under previous conditions.

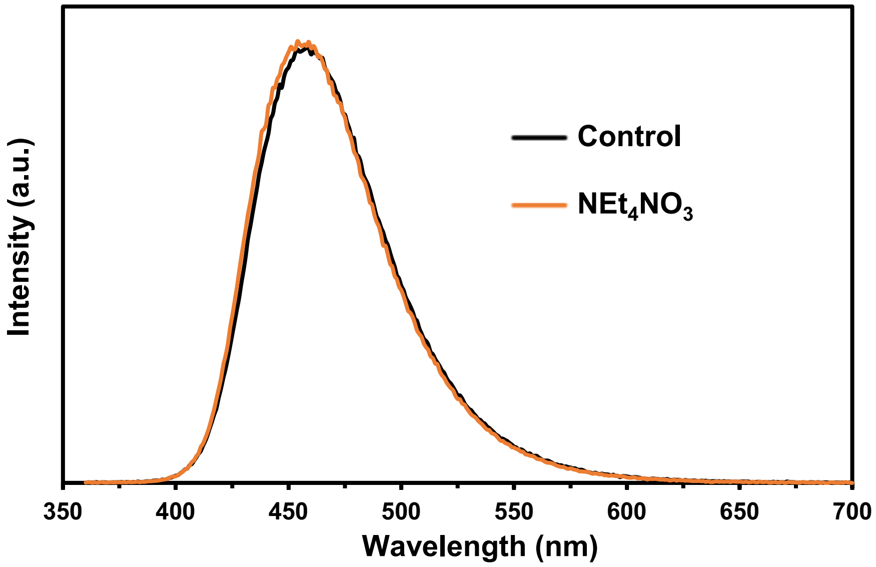

Figure S21. Fluorescence emission spectra of 1-NH_2^{4+} (0.2 mM) with 1000 equivalents of TEA in methanol (black) and adding 1000 equivalents of NEt_4NO_3 (orange). The excitation wavelength was 350 nm.

S.8.2. $LiNO_3$ addition

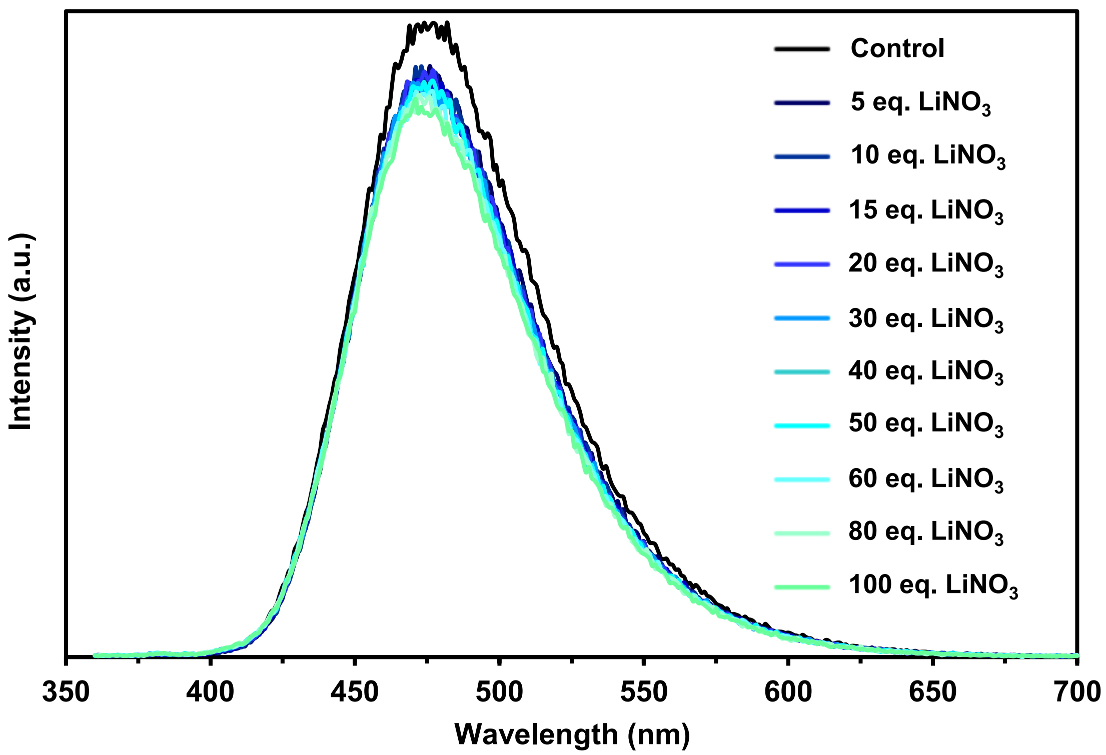

Figure S22. Fluorescence emission spectra of 1-NH_2^{4+} (0.2 mM) in methanol in the presence of increasing equivalents of $LiNO_3$. The excitation wavelength was 350 nm.

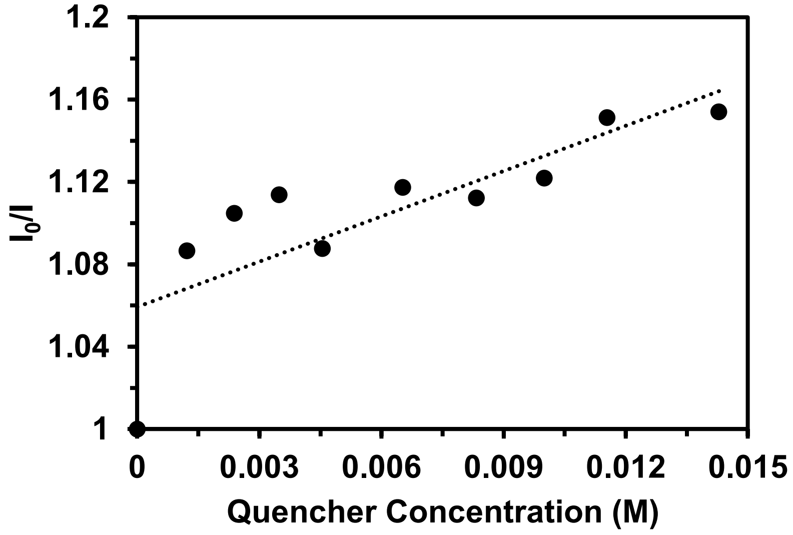

Figure S23. Stern-Volmer plot of I_0/I versus quencher concentration ($LiNO_3$) under previous conditions.

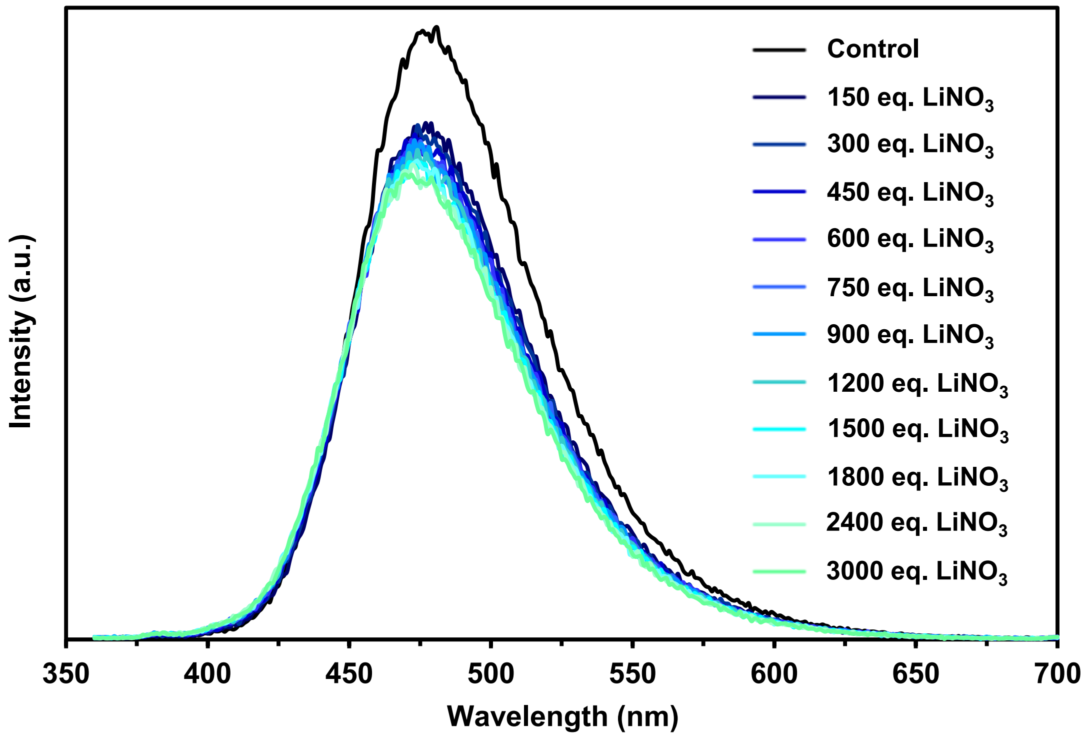

Figure S24. Fluorescence emission spectra of 1-NH_2^{4+} (0.2 mM) in methanol in the presence of increasing equivalents of $LiNO_3$. The excitation wavelength was 350 nm.

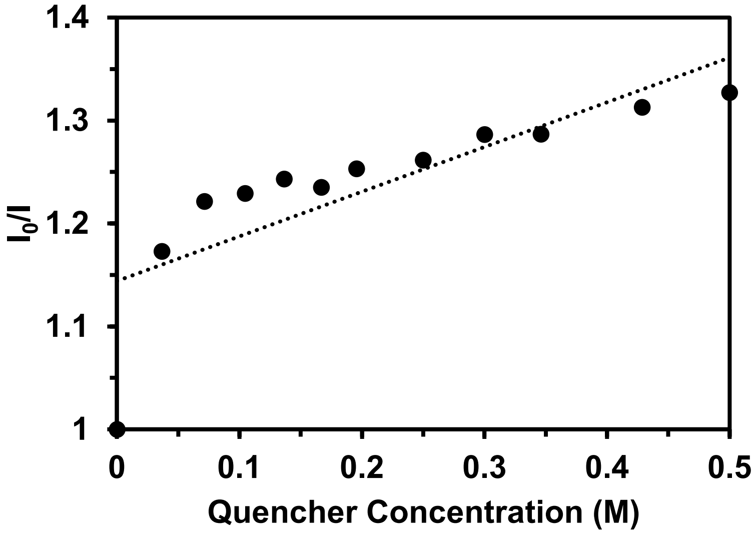

Figure S25. Stern-Volmer plot of I_0/I versus quencher concentration ($LiNO_3$) under previous conditions.

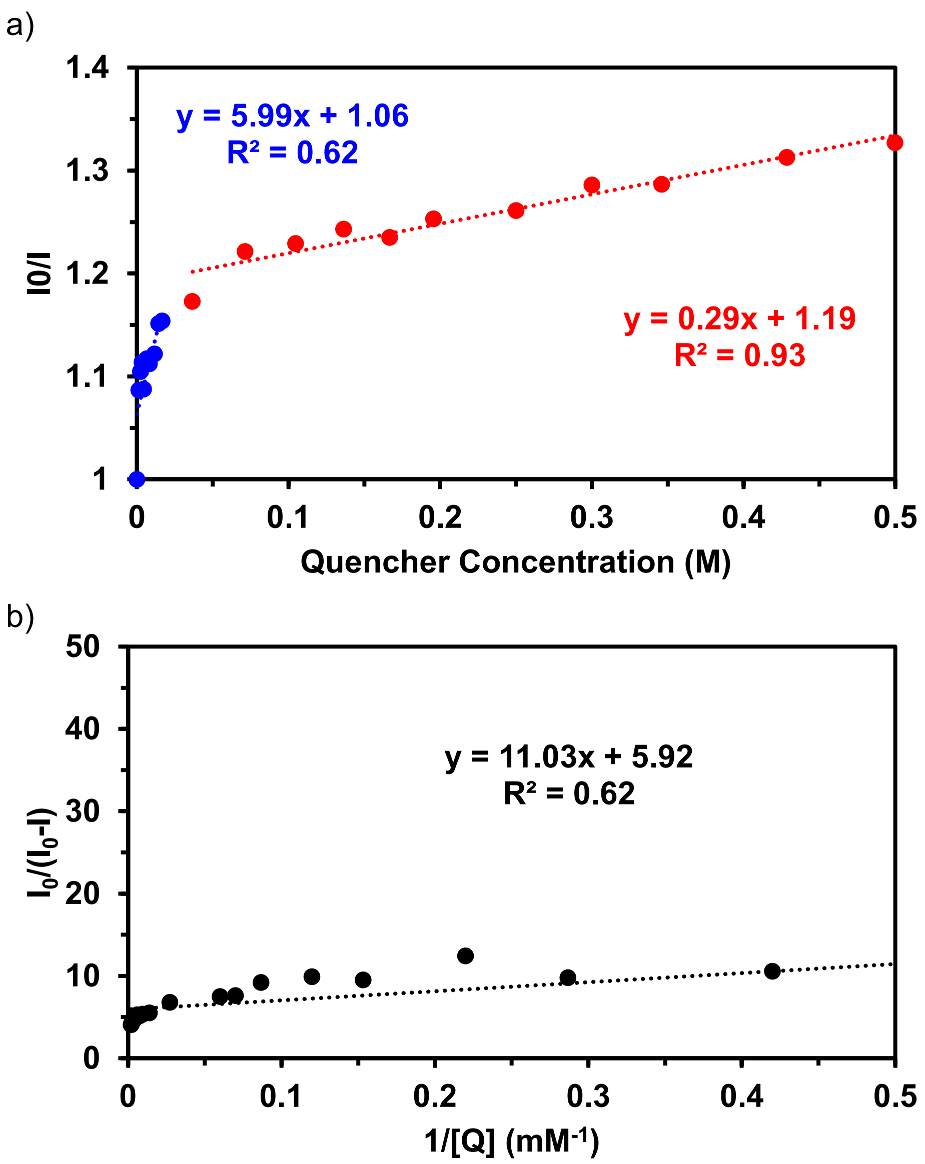

Figure S26. (a) Stern-Volmer plot of I_0/I versus [$LiNO_3$] with the linear fit for the two identified regions. (b) Lehrer plot of $I_0/(I_0-I)$ versus 1/[$LiNO_3$] with the corresponding linear fit.

S.8.3. $AgNO_3$ addition

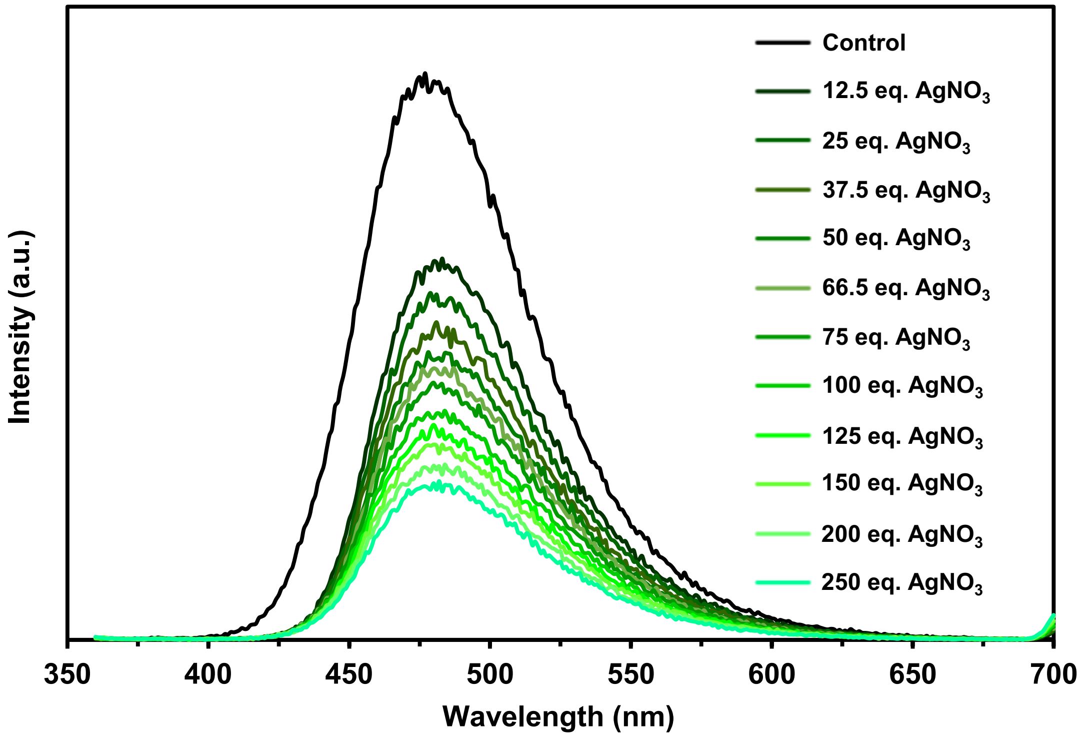

Figure S27. Fluorescence emission spectra of 1-NH_2^{4+} (0.2 mM) in methanol in the presence of increasing equivalents of $AgNO_3$. The excitation wavelength was 350 nm.

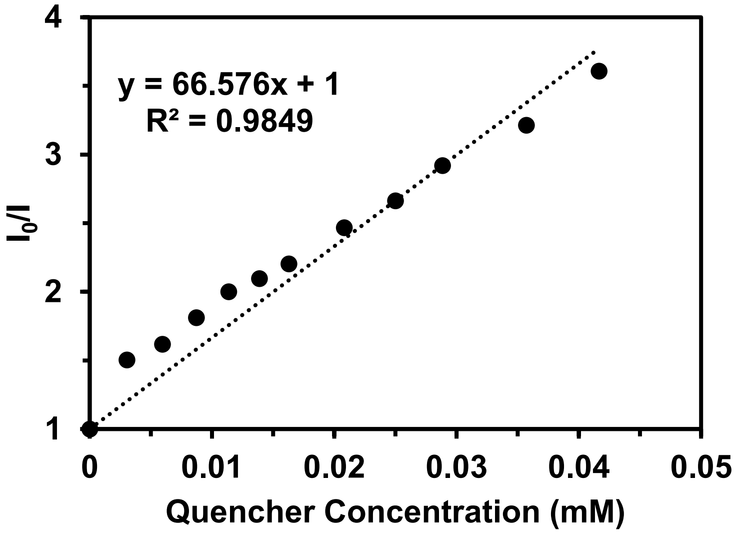

Figure S28. Stern-Volmer plot of I_0/I versus quencher concentration ($AgNO_3$) under previous conditions.

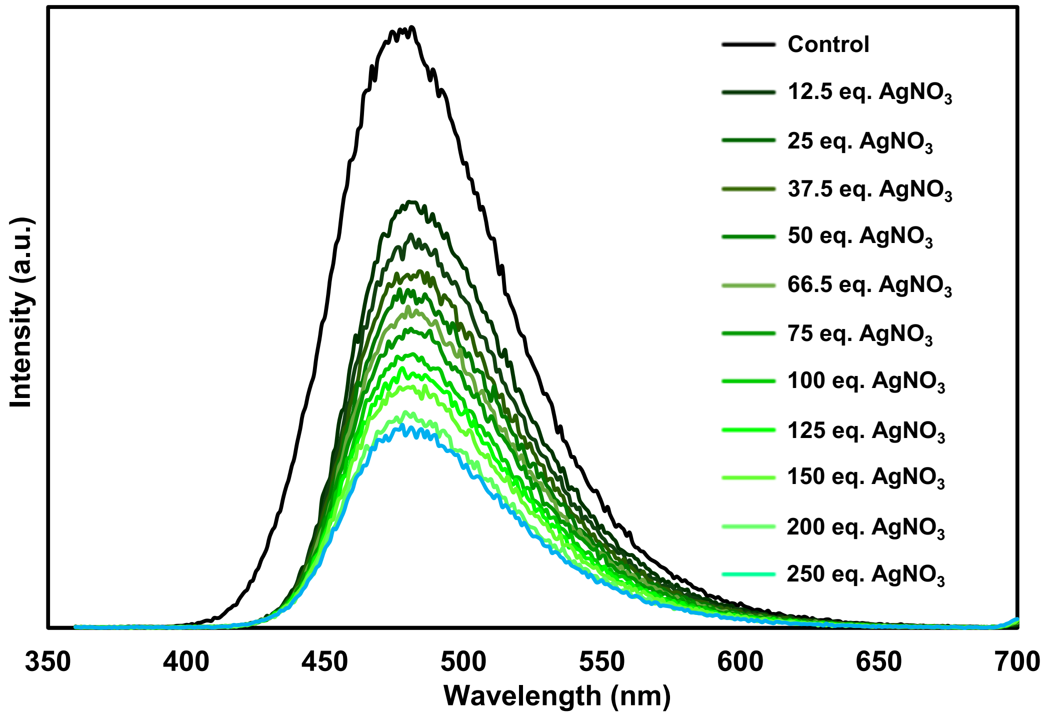

Figure S29. Fluorescence emission spectra of 1-NH_2^{4+} (0.2 mM) in **methanol-d4** in the presence of increasing equivalents of $AgNO_3$. The excitation wavelength was 350 nm.

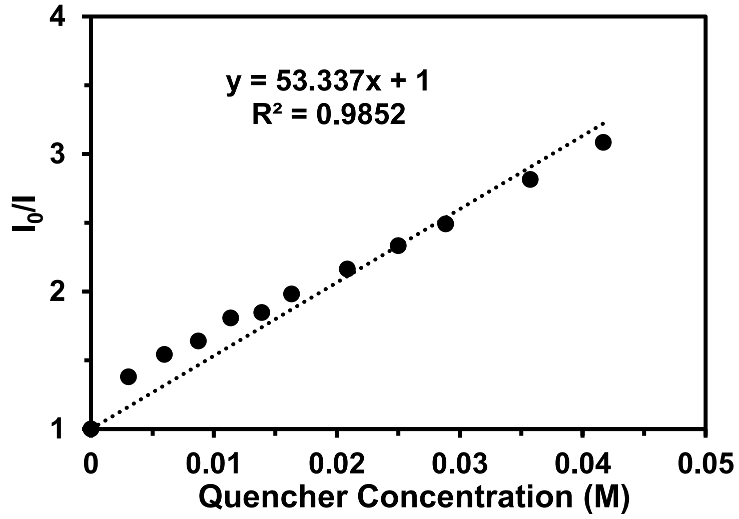

Figure S30. Stern-Volmer plot of I_0/I versus quencher concentration ($AgNO_3$) under previous conditions.

S.8.4. AgOTf addition

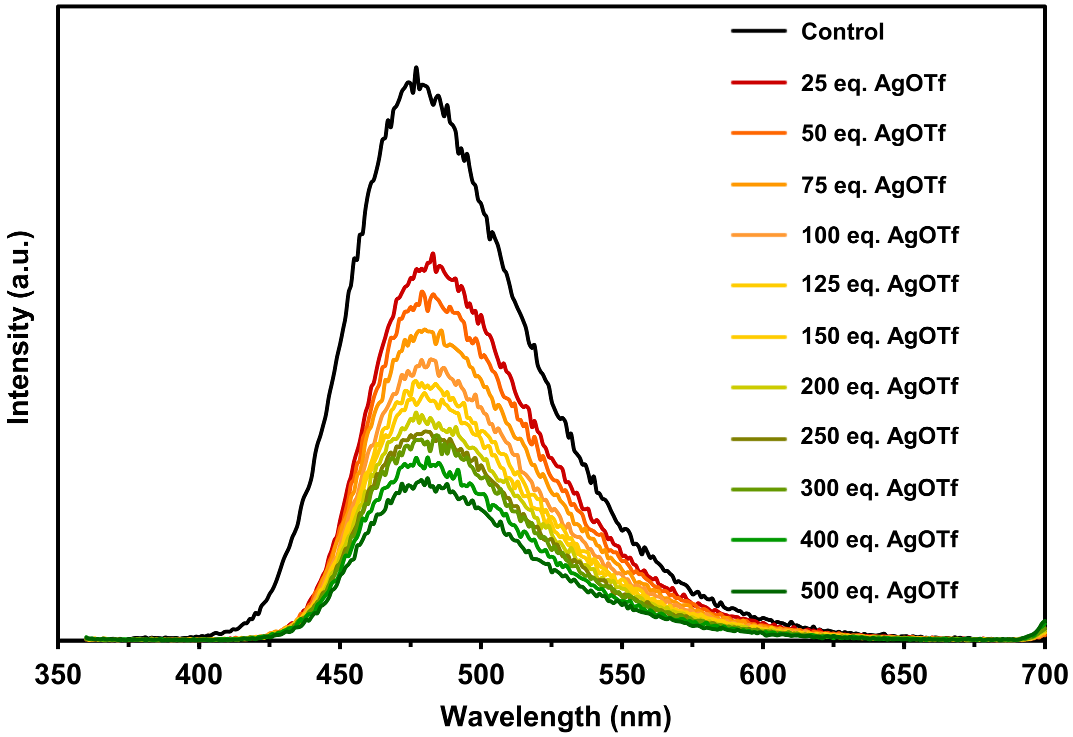

Figure S31. Fluorescence emission spectra of 1-NH_2^{4+} (0.2 mM) in methanol in the presence of increasing equivalents of AgOTf. The excitation wavelength was 350 nm.

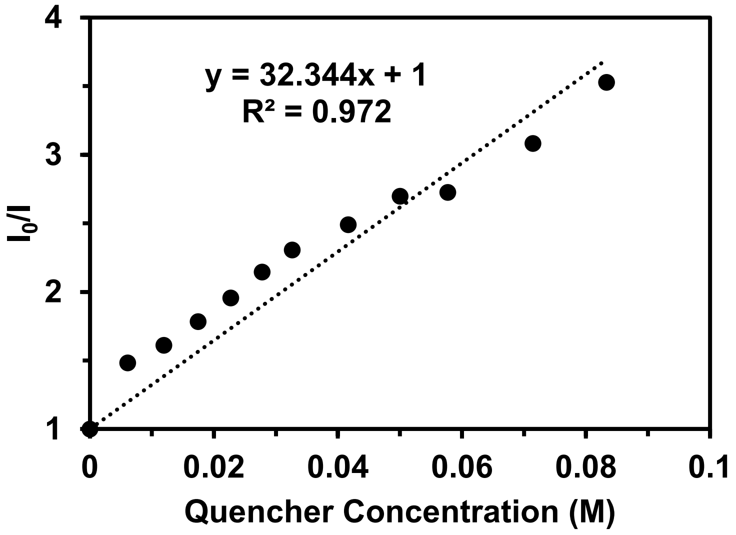

Figure S32. Stern-Volmer plot of I_0/I versus quencher concentration (AgOTf) under previous conditions.

S.8.5. H_2O addition

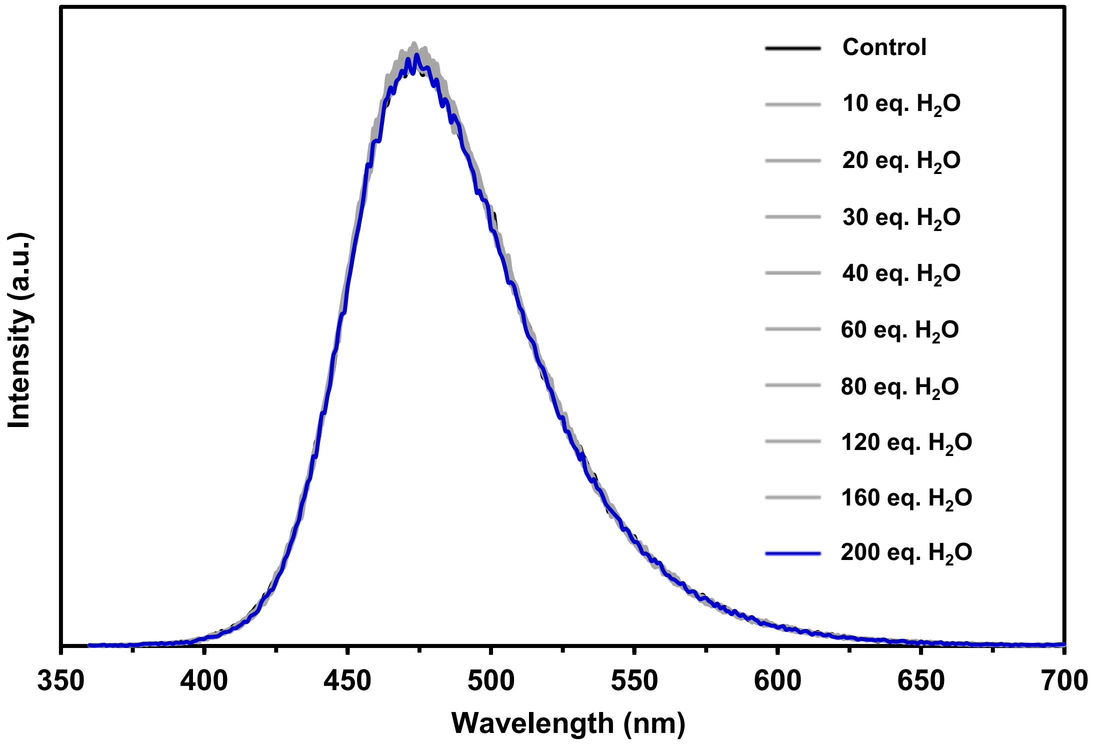

Figure S33. Fluorescence emission spectra of 1-NH_2^{4+} (0.2 mM) in methanol in the presence of increasing equivalents of H_2O. The excitation wavelength was 350 nm.

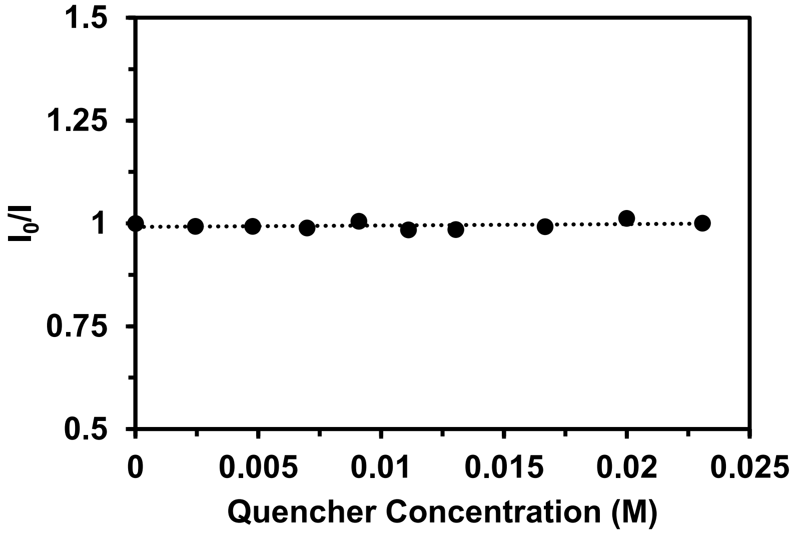

Figure S34. Stern-Volmer plot of I_0/I versus quencher concentration (H_2O) under previous conditions.

S.8.6. $NaNO_2$ addition

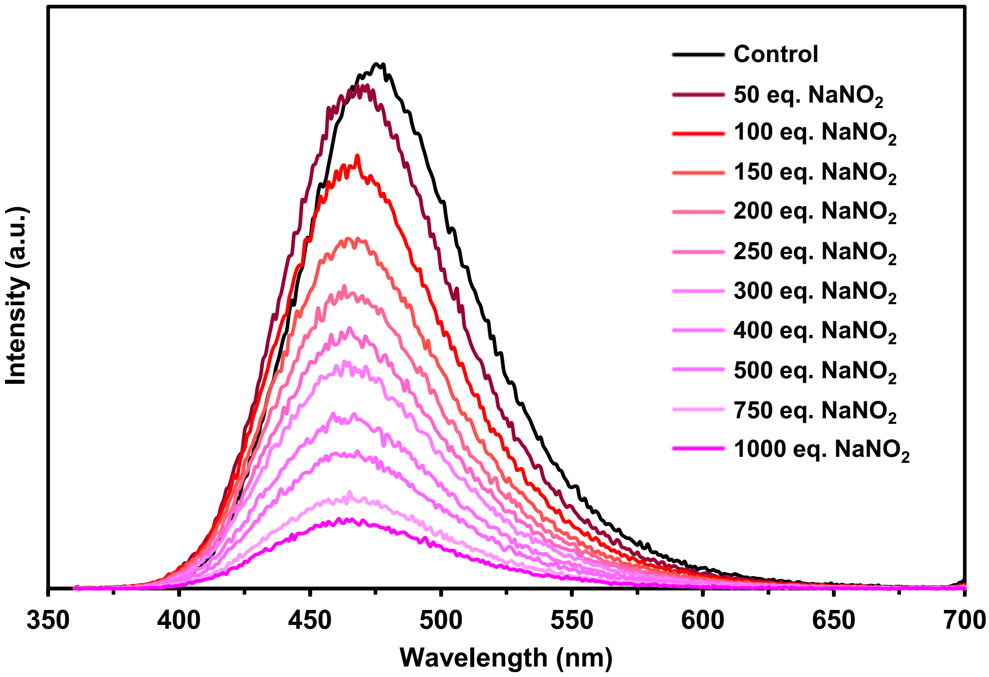

Figure S35. Fluorescence emission spectra of 1-NH_2^{4+} (0.2 mM) in methanol in the presence of increasing equivalents of $NaNO_2$. The excitation wavelength was 350 nm.

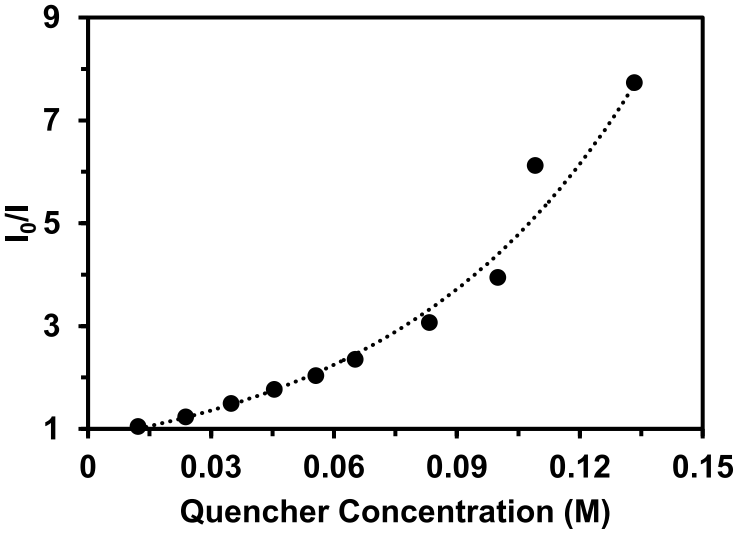

Figure S36. Stern-Volmer plot of I_0/I versus quencher concentration ($NaNO_2$) under previous conditions.

S.9. Relevant photocatalytic reactions

Photocatalytic reactions were carried out under nitrogen atmosphere. In a 5 mL glass vial 1-NH_2^{4+} (1 μmol), substrate (50 μmol), sacrificial electron donor (100 or 400 μmol) and solvent (mixture of 2 mL) were added. The mixture was stirred and irradiated with blue light (440 nm wavelength, 100 W KESSIL LIGHT LEDs) during 24h. Then, 100 ul of HCl was added to the solution and wait for 1h. The solvent was evaporated and the solid powder was dissolved in 800 μL of deuterated dimethylsulfoxide with maleic acid as internal standard (50 μmol), dried over sodium sulphate and analyzed by for ^{1}H NMR. Ammonia was alternatively quantified by Ion Chromatography. To this aim, the resulting solid after work up was dissolved in 1 mL MilliQ water, and used as sample for the Ion Chromatography quantification.

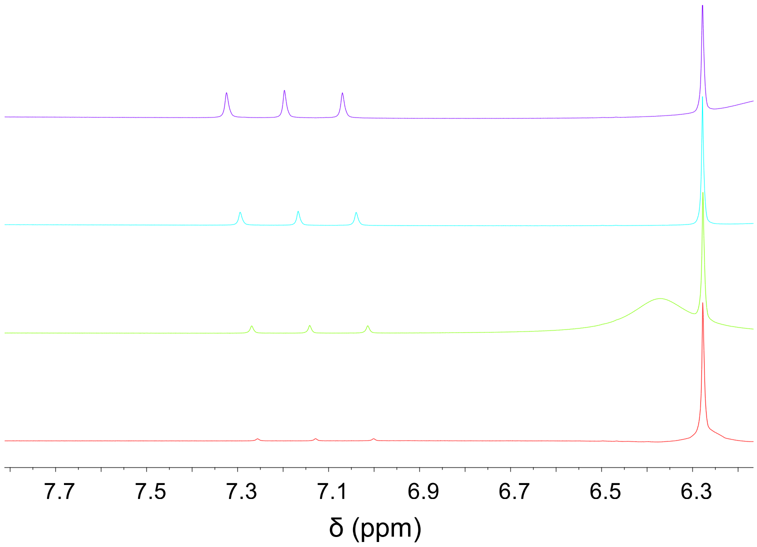

Figure S37. ^{1}H NMR spectra in DMSO-d6 of solutions with increasing concentration of NH_4Cl used for the calibration curve with maleic acid as the internal standard.

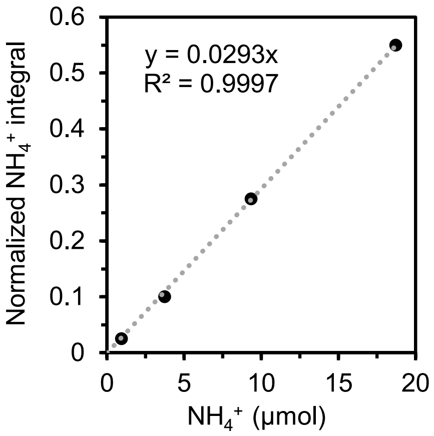

Figure S38. Calibration for the NH_4Cl quantification via ^{1}H NMR.

S.9.1. Control reactions

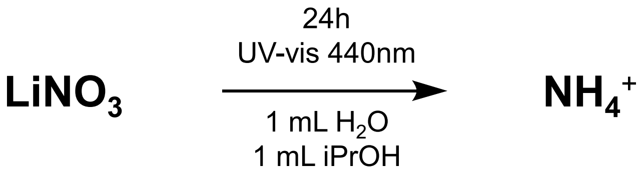

Figure S39. Scheme of the photocatalytic reaction without catalyst using $LiNO_3$ as the substrate.

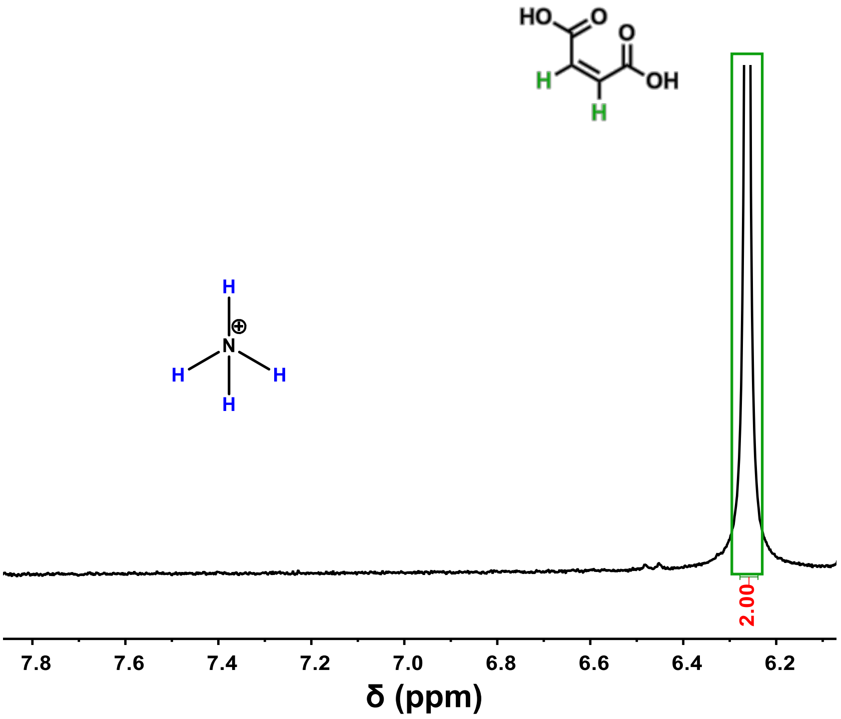

Figure S40. ^{1}H NMR spectra in DMSO-d6 of the photocatalytic reaction without catalyst using $LiNO_3$, showing the internal standard signal (maleic acid, green).

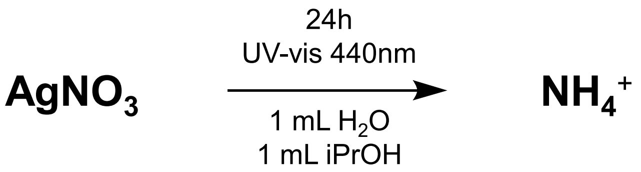

Figure S41. Scheme of the photocatalytic reaction without catalyst using $AgNO_3$ as the substrate.

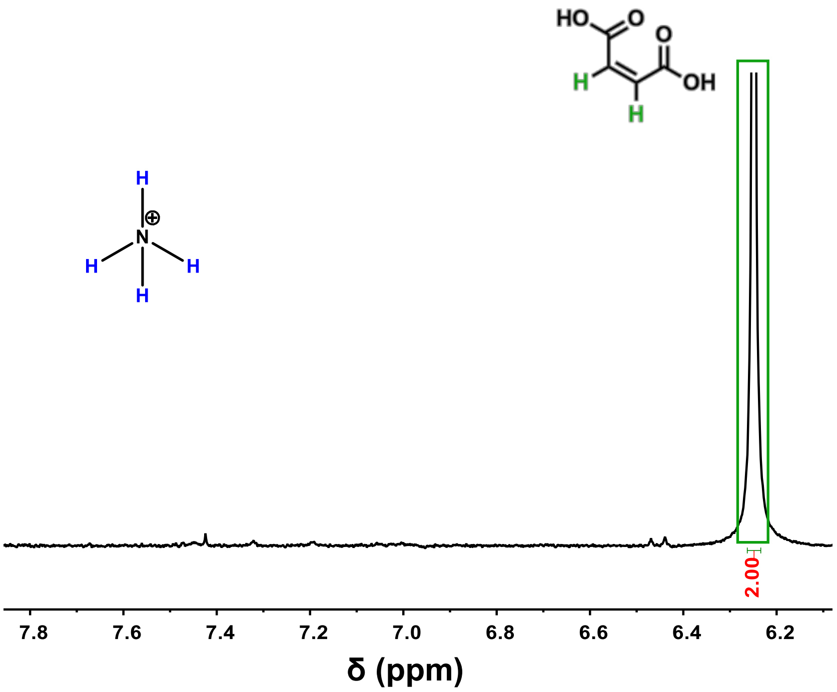

Figure S42. ^{1}H NMR spectra in DMSO-d6 of the photocatalytic reaction without catalyst using $AgNO_3$, showing the internal standard signal (maleic acid, green).

S.9.2. Lewis acids

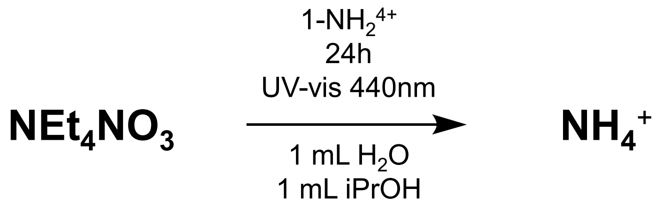

Figure S43. Scheme of the photocatalytic reaction mediated by 1-NH_2^{4+} in H_2O:iPrOH using NEt_4NO_3 as the substrate.

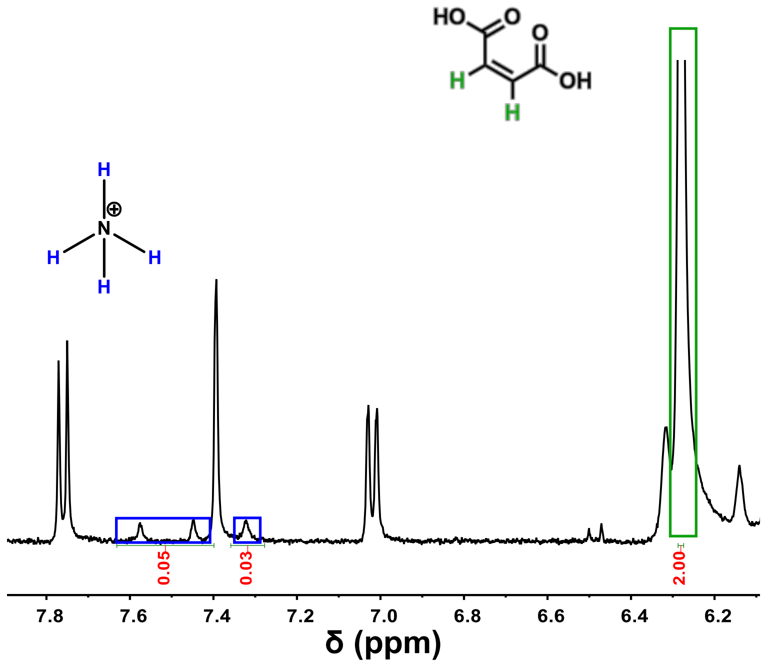

Figure S44. ^{1}H NMR spectra in DMSO-d6 of the photocatalytic reaction trying NEt_4NO_3, showing the ammonia signal (blue) and the internal standard signal (maleic acid, green).

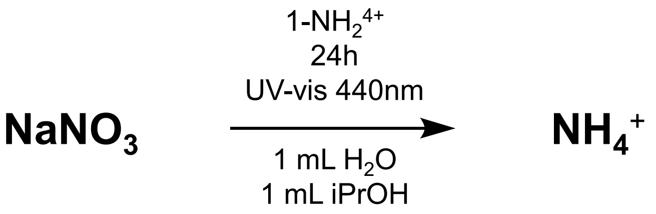

Figure S45. Scheme of the photocatalytic reaction mediated by 1-NH_2^{4+}in H_2O:iPrOH using $NaNO_3$ as the substrate.

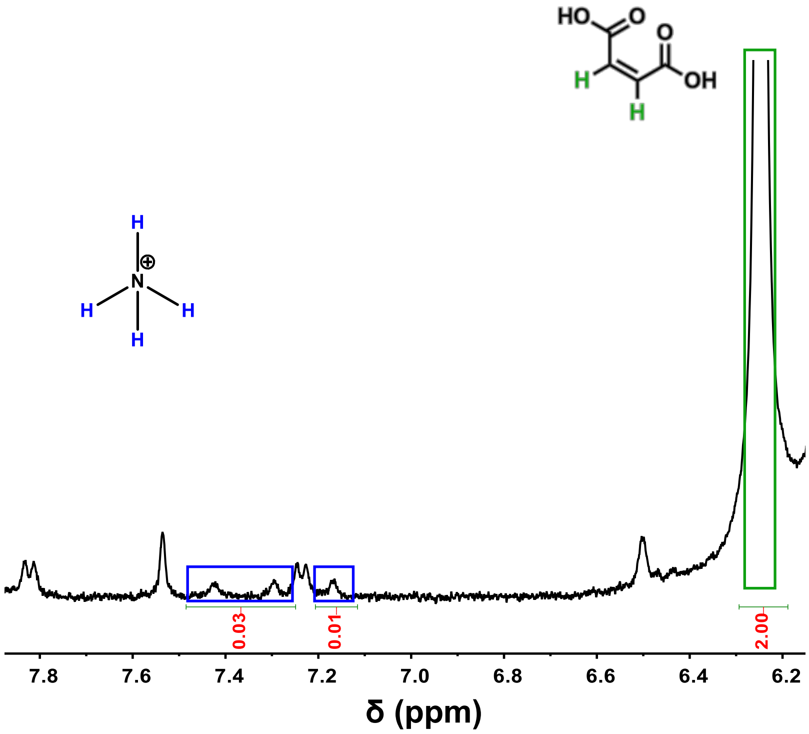

Figure S46. ^{1}H NMR spectra in DMSO-d6 of the photocatalytic reaction trying $NaNO_3$, showing the ammonia signal (blue) and the internal standard signal (maleic acid, green).***Note**: *the signals at 7.9, 7.55 and 7.25 ppm correspond to the terephthalate linker of the Zr-cage resulting from partial degradation after 24 h of photocatalytic run.*

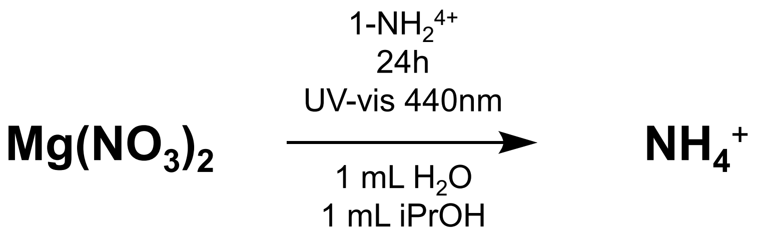

Figure S47. Scheme of the photocatalytic reaction mediated by 1-NH_2^{4+}in H_2O:iPrOH using $Mg(NO_3)_2$ as the substrate.

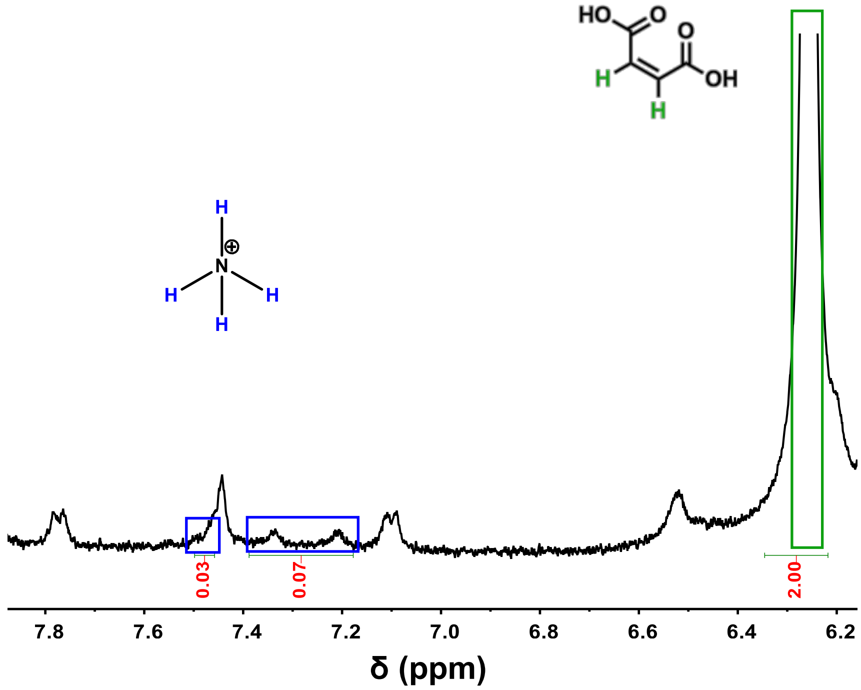

Figure S48. ^{1}H NMR spectra in DMSO-d6 of the photocatalytic reaction trying $Mg(NO_3)_2$, showing the ammonia signal (blue) and the internal standard signal (maleic acid, green).

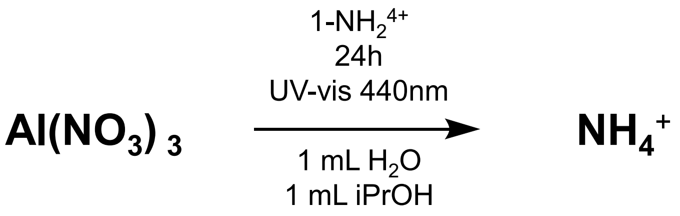

Figure S49. Scheme of the photocatalytic reaction mediated by 1-NH_2^{4+} in H_2O:iPrOH using $Al(NO_3)_3$ as the substrate.

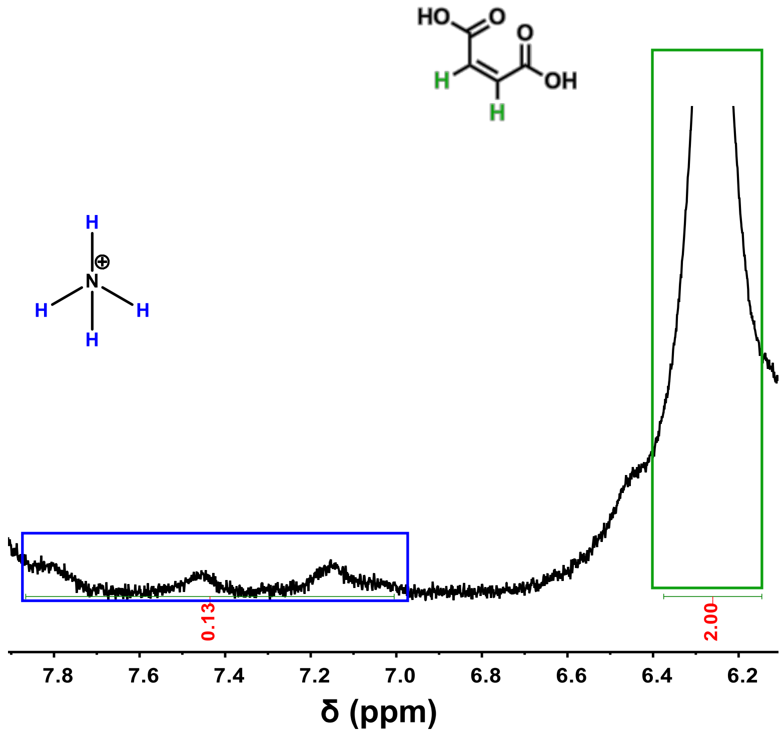

Figure S50. ^{1}H NMR spectra in DMSO-d6 of the photocatalytic reaction trying $Al(NO_3)_3$, showing the ammonia signal (blue) and the internal standard signal (maleic acid, green).

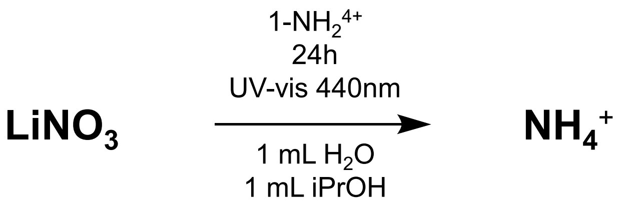

Figure S51. Scheme of the photocatalytic reaction mediated by 1-NH_2^{4+} in H_2O:iPrOH using $LiNO_3$ as the substrate.

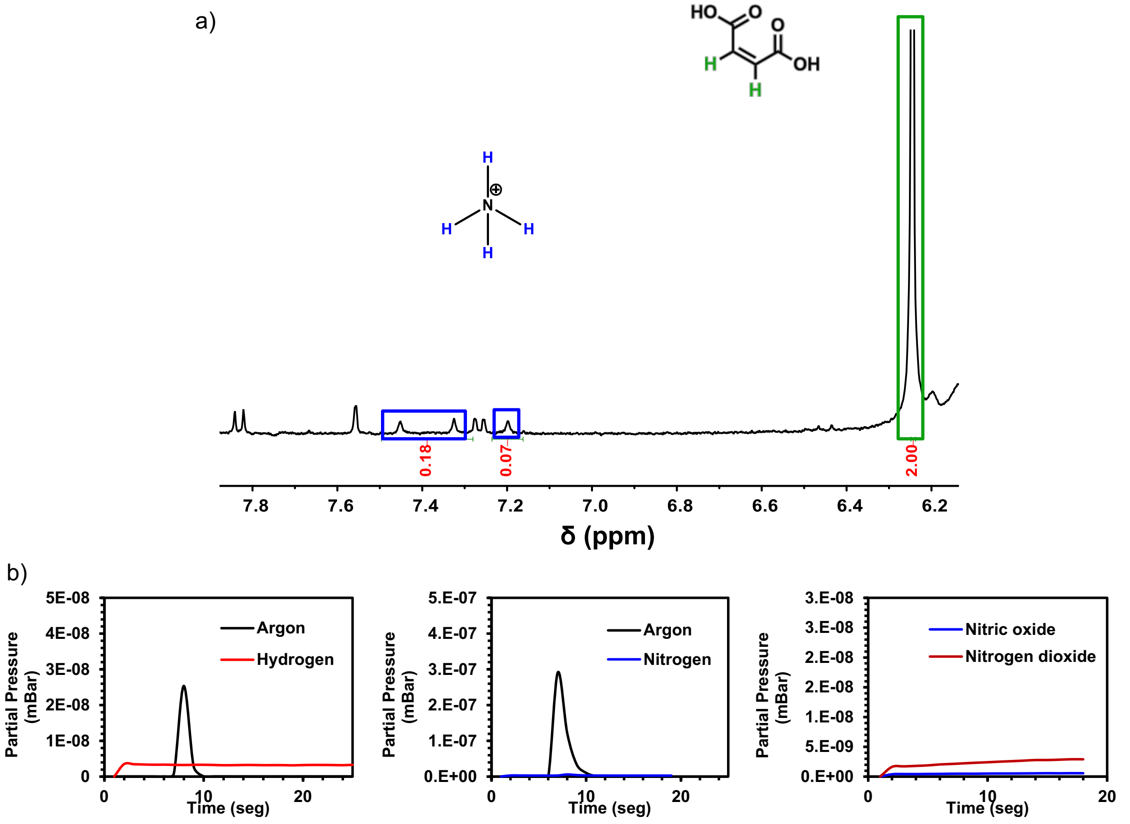

Figure S52. (a) ^{1}H NMR spectra in DMSO-d6 of the photocatalytic reaction trying $LiNO_3$, showing the ammonia signal (blue) and the internal standard signal (maleic acid, green). (b) MS trace for the detection and quantification of the different gases produced during the photocatalytic run under Ar atmosphere. In all cases, the amount of gas produced remained below 0.2 equiv. respect to the amount of 1-NH_2^{4+}. **Note**: quantification of ammonium ion by ion chromatography results in an average concentration of 331.15 ± 25 mg/L that corresponds to an average of 4.8 µmol of NH_3, consistent with the NMR quantification method.

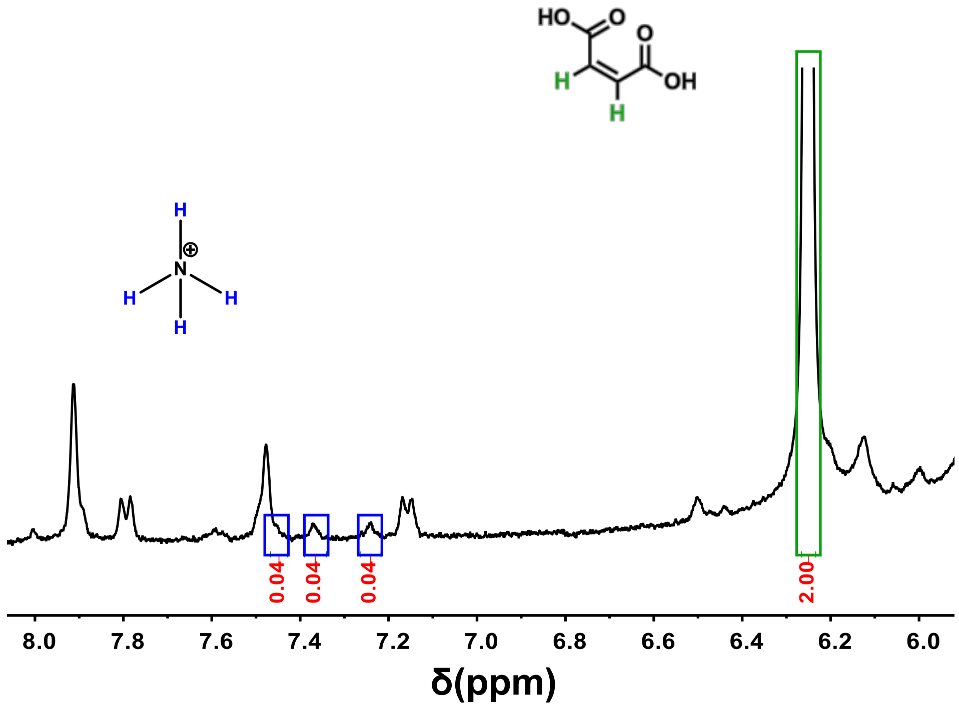

Figure S53. ^{1}H NMR spectra in DMSO-d6 of the photocatalytic reaction trying $LiNO_3$, showing the ammonia signal (blue) and the internal standard signal (maleic acid, green).

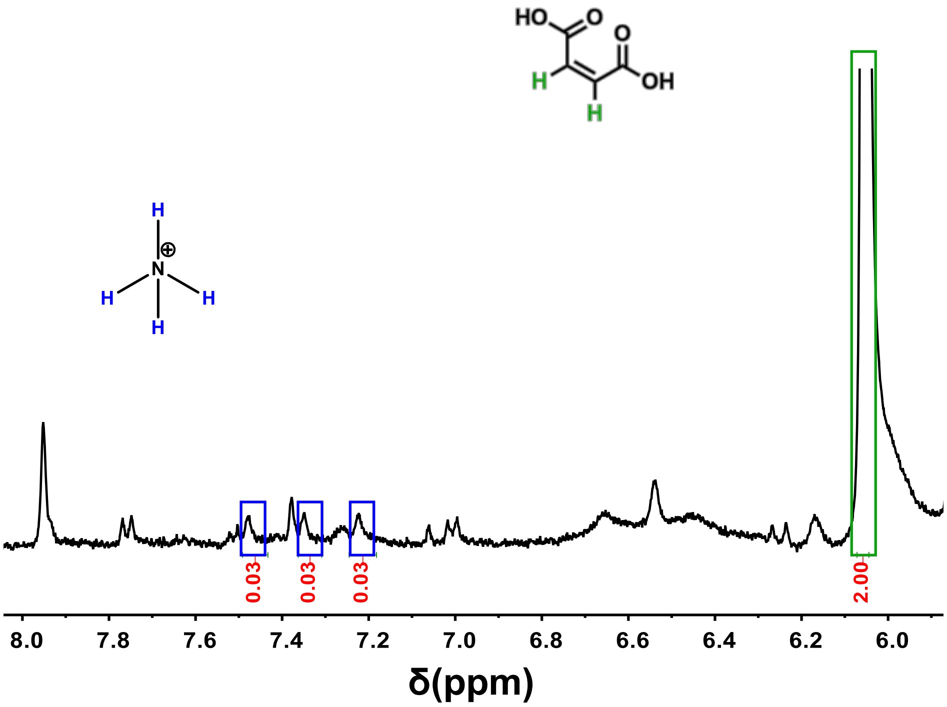

Figure S54. ^{1}H NMR spectra in DMSO-d6 of the photocatalytic reaction trying $LiNO_3$, showing the ammonia signal (blue) and the internal standard signal (maleic acid, green).

S.9.3. ^{15}N-labelled nitrate

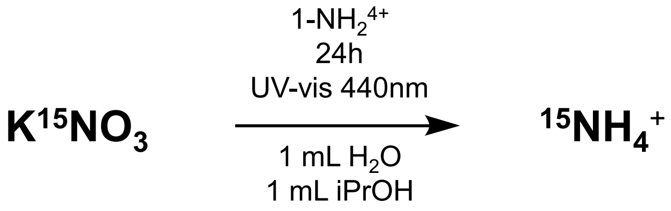

Figure S55. Scheme of the photocatalytic reaction mediated by 1-NH_2^{4+} in H_2O:MeCN using $LiNO_3$ as the substrate.

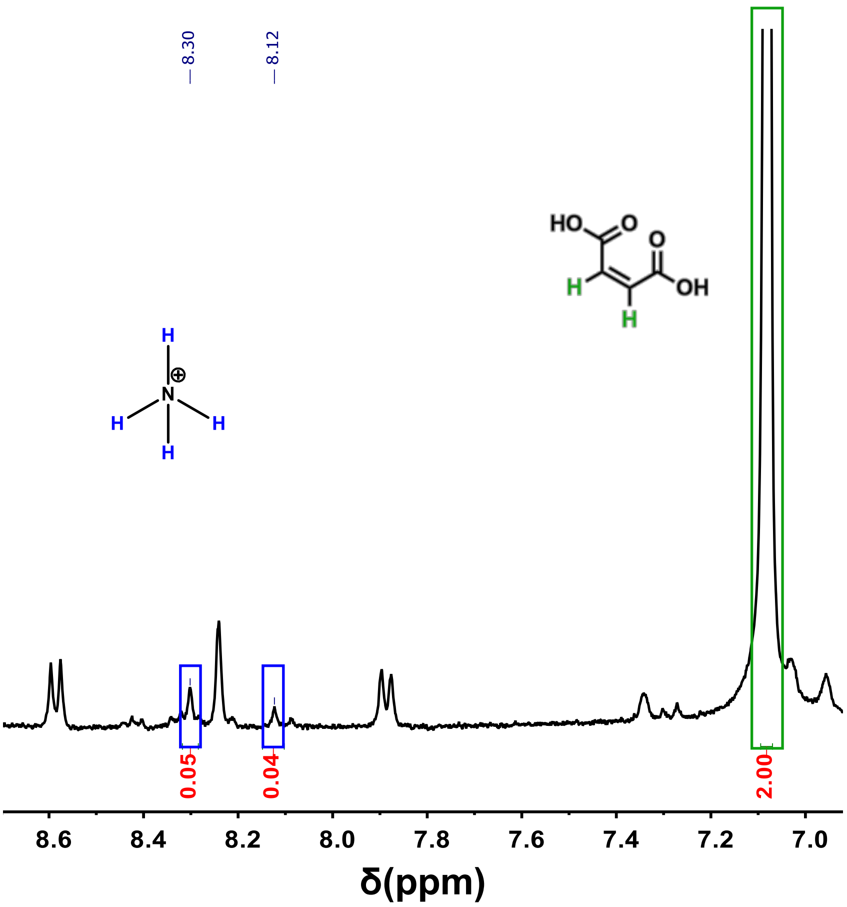

Figure S56. ^{1}H NMR spectra in DMSO-d6 of the photocatalytic reaction trying $K^{15}NO_3$, showing the ammonia signal (blue), the displacement of these peaks and the internal standard signal (maleic acid, green). The calculated J_{15NH} is 72 MHz consistent with the value for $^{15}NH_3$.

S.9.4. Solvents

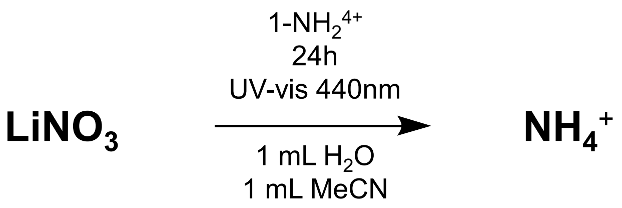

Figure S57. Scheme of the photocatalytic reaction mediated by 1-NH_2^{4+} in H_2O:MeCN using $LiNO_3$ as the substrate.

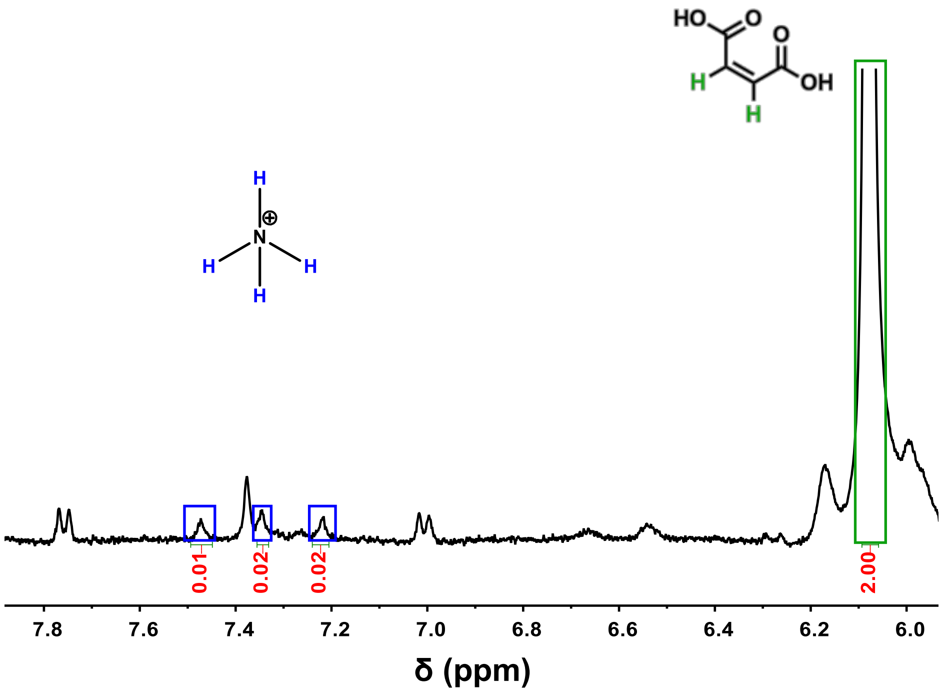

Figure S58. ^1H NMR spectra in DMSO-d6 of the photocatalytic reaction trying $LiNO_3$, showing the ammonia signal (blue) and the internal standard signal (maleic acid, green).

S.9.5. Sacrificial electron donors

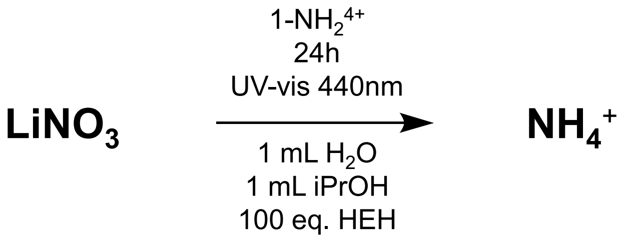

Figure S59. Scheme of the photocatalytic reaction mediated by 1-NH_2^{4+} in H_2O:iPrOH using $LiNO_3$ as the substrate and 100 equivalents of HEH as sacrificial electron donor.

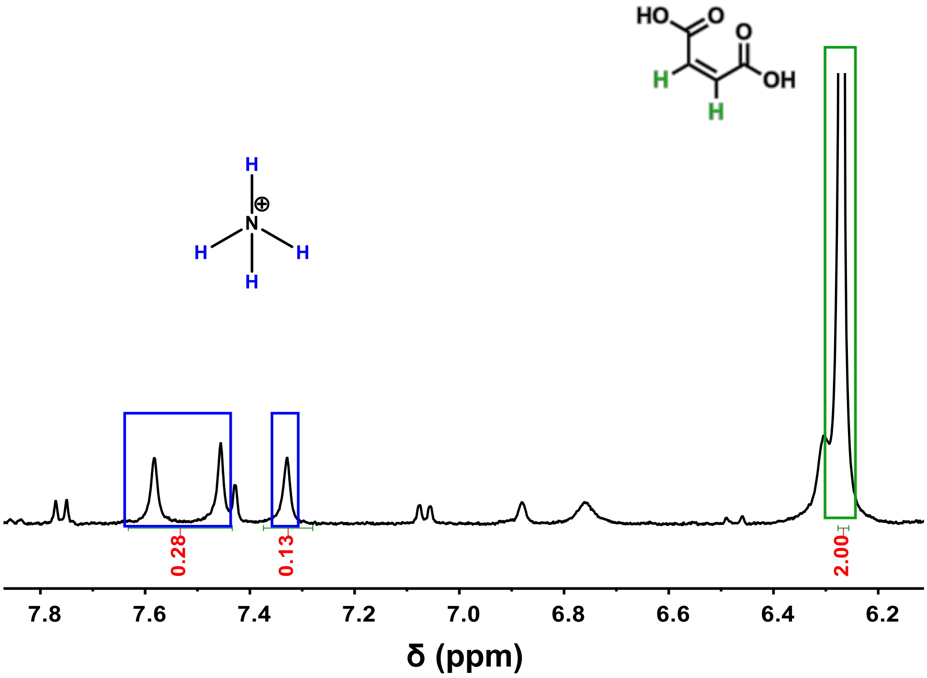

Figure S60. ^{1}H NMR spectra in DMSO-d6 of the photocatalytic reaction trying $LiNO_3$ in presence of 100 equivalents of HEH, showing the ammonia signal (blue) and the internal standard signal (maleic acid, green).

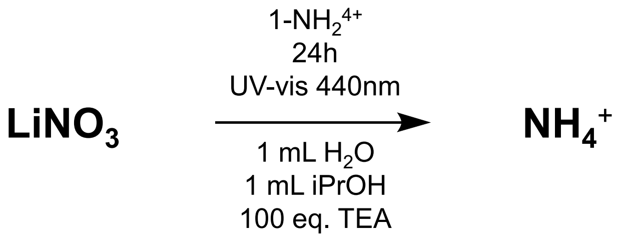

Figure S61. Scheme of the photocatalytic reaction mediated by 1-NH_2^{4+} in H_2O:iPrOH using $LiNO_3$ as the substrate and 100 equivalents of TEA as sacrificial electron donor.

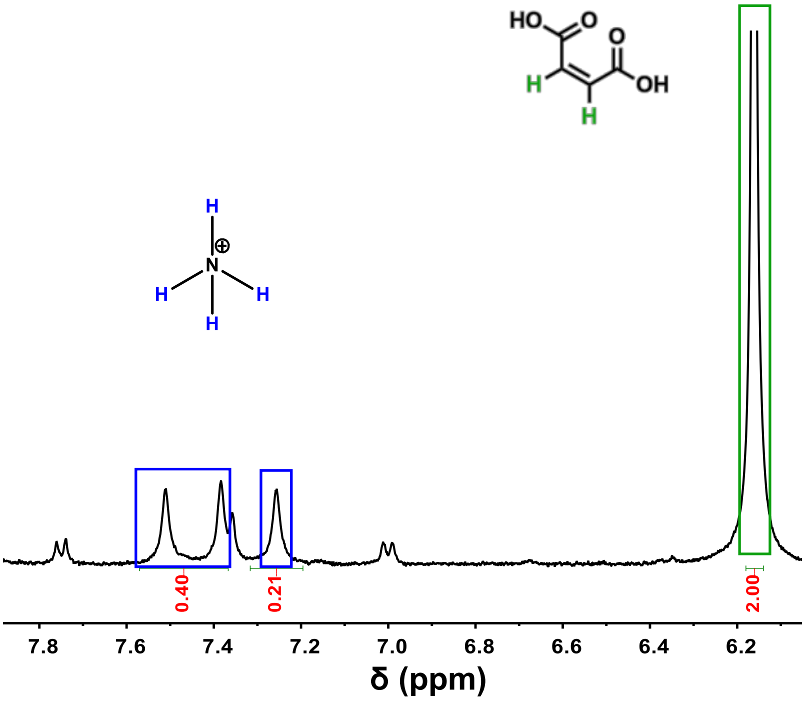

Figure S62. ^{1}H NMR spectra in DMSO-d6 of the photocatalytic reaction trying $LiNO_3$ in presence of 100 equivalents of TEA, showing the ammonia signal (blue) and the internal standard signal (maleic acid, green).

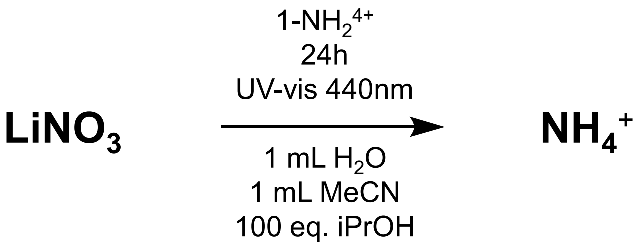

Figure S63. Scheme of the photocatalytic reaction mediated by 1-NH_2^{4+} in H_2O:MeCN using $LiNO_3$ as the substrate and 100 equivalents of iPrOH as sacrificial electron donor.

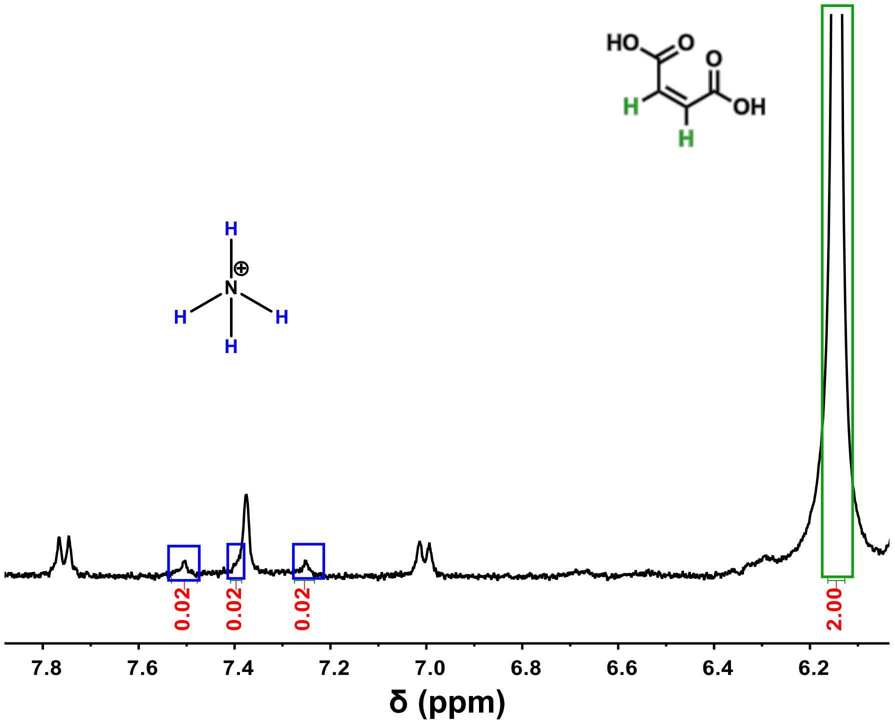

Figure S64. ^{1}H NMR spectra in DMSO-d6 of the photocatalytic reaction trying $LiNO_3$ in MeCN in presence of 100 equivalents of iPrOH, showing the ammonia signal (blue) and the internal standard signal (maleic acid, green).

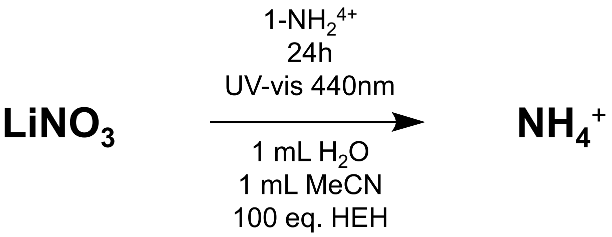

Figure S65. Scheme of the photocatalytic reaction mediated by 1-NH_2^{4+} in H_2O:MeCN using $LiNO_3$ as the substrate and 100 equivalents of HEH as sacrificial electron donor.

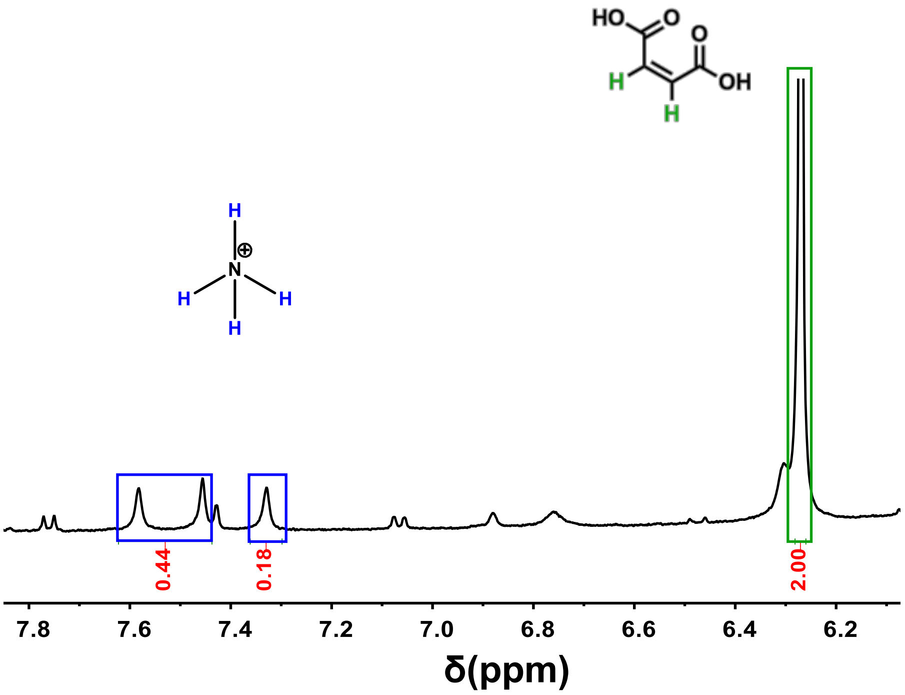

Figure S66. ^{1}H NMR spectra in DMSO-d6 of the photocatalytic reaction trying $LiNO_3$ in MeCN in presence of 100 equivalents of HEH, showing the ammonia signal (blue) and the internal standard signal (maleic acid, green).

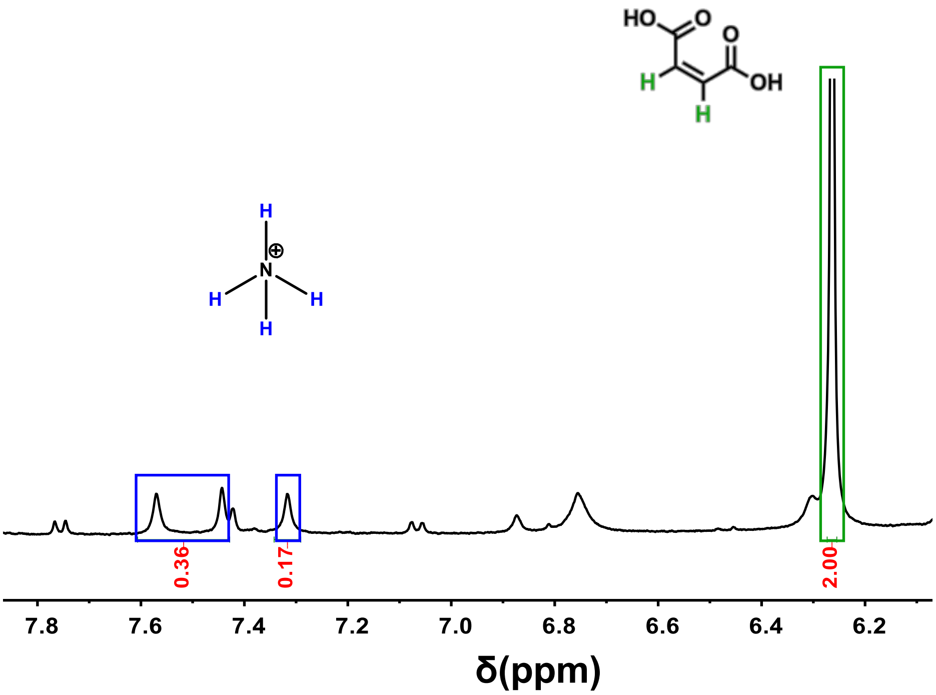

Figure S67. ^{1}H NMR spectra in DMSO-d6 of the photocatalytic reaction trying $LiNO_3$ in MeCN in presence of 100 equivalents of HEH, showing the ammonia signal (blue) and the internal standard signal (maleic acid, green).

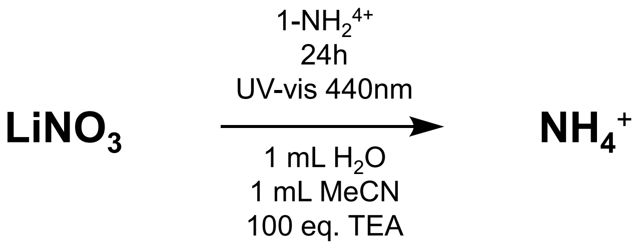

Figure S68. Scheme of the photocatalytic reaction mediated by $1\text{-}NH_2^{4+}$ in H_2O:MeCN using $LiNO_3$ as the substrate and 100 equivalents of TEA as sacrificial electron donor.

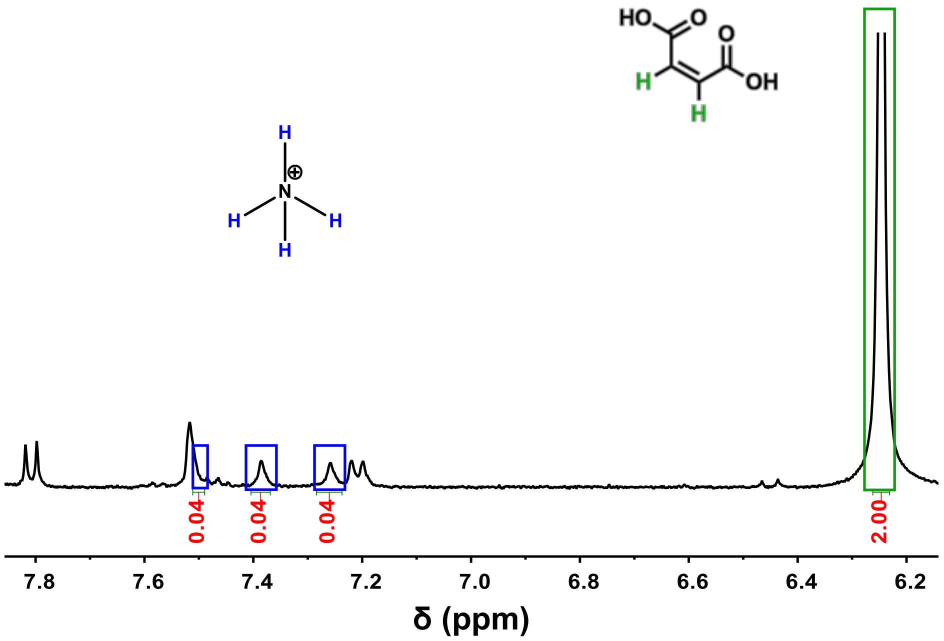

Figure S69. ^{1}H NMR spectra in DMSO-d6 of the photocatalytic reaction trying $LiNO_3$ in MeCN in presence of 100 equivalents of TEA, showing the ammonia signal (blue) and the internal standard signal (maleic acid, green).

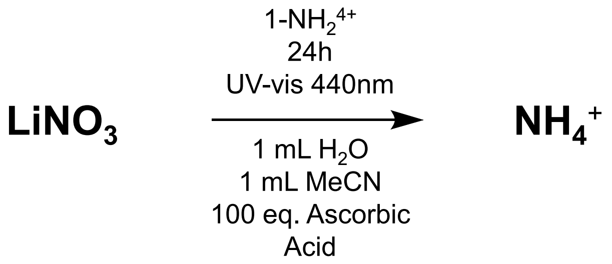

Figure S70. Scheme of the photocatalytic reaction mediated by 1-NH_2^{4+} in H_2O:MeCN using $LiNO_3$ as the substrate and 100 equivalents of ascorbic acid as sacrificial electron donor.

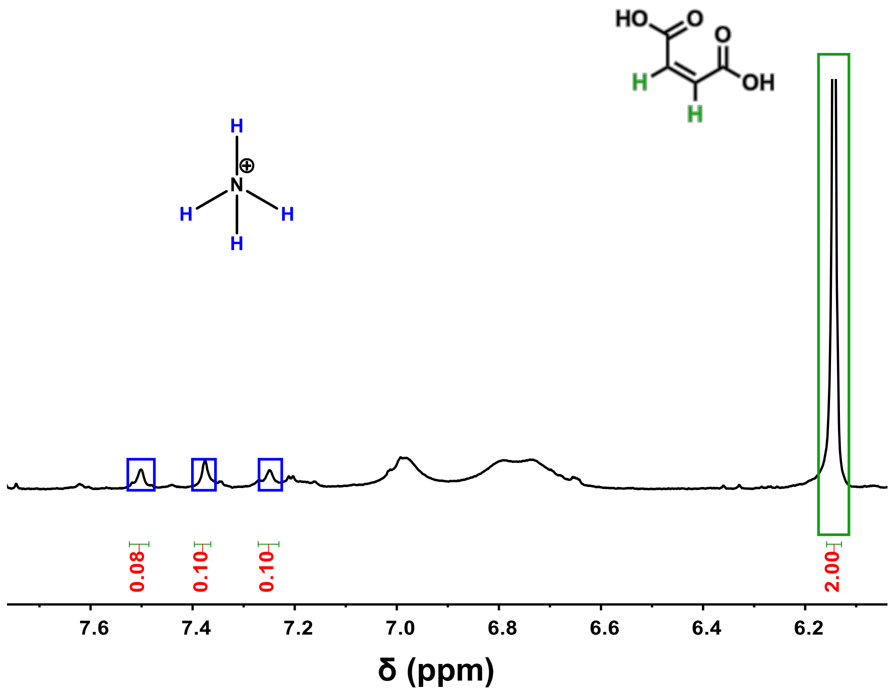

Figure S71. ^1H NMR spectra in DMSO-d6 of the photocatalytic reaction trying $LiNO_3$ in MeCN in presence of 100 equivalents of ascorbic acid, showing the ammonia signal (blue) and the internal standard signal (maleic acid, green).

S.9.6. Reduction of potential intermediates

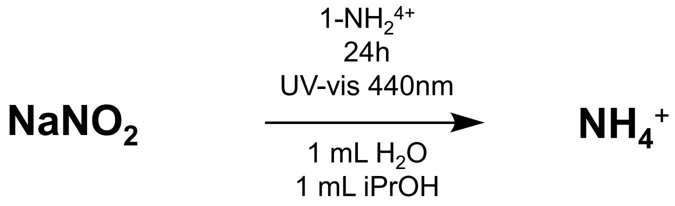

Figure S72. Scheme of the photocatalytic reaction mediated by 1-NH_2^{4+}in H_2O:iPrOH using $NaNO_2$ as the substrate.

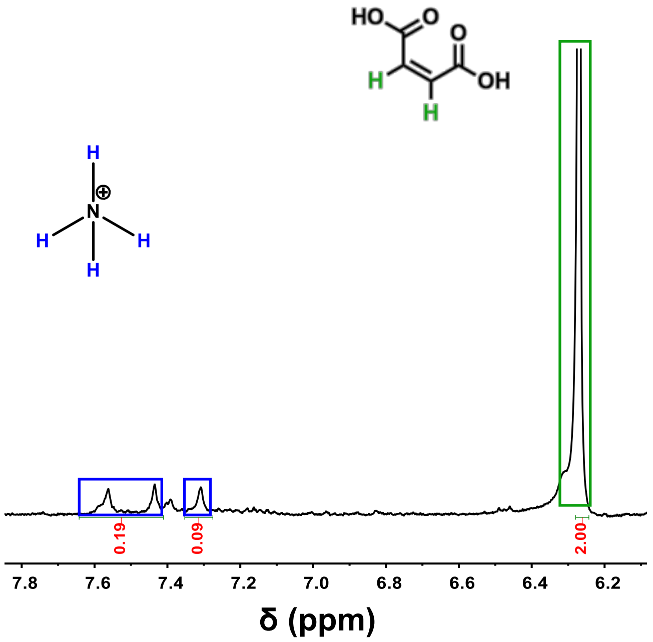

Figure S73. ^{1}H NMR spectra in DMSO-d6 of the photocatalytic reaction trying $NaNO_2$, showing the ammonia signal (blue) and the internal standard signal (maleic acid, green).

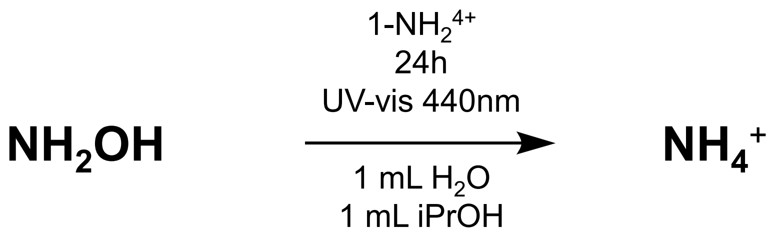

Figure S74. Scheme of the photocatalytic reaction mediated by 1-NH_2^{4+} in H_2O:iPrOH using NH_2OH as the substrate.

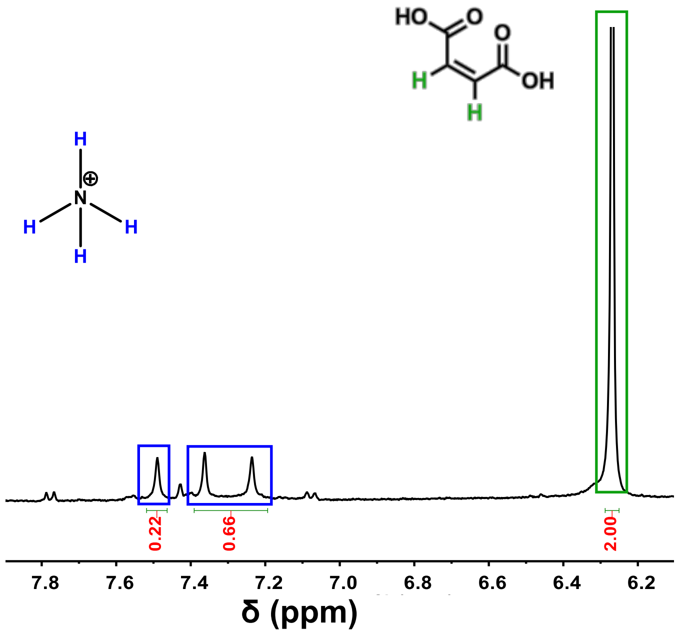

Figure S75. ^{1}H NMR spectra in DMSO-d6 of the photocatalytic reaction trying NH_2OH, showing the ammonia signal (blue) and the internal standard signal (maleic acid, green).

S.9.7. Ag^+ addition

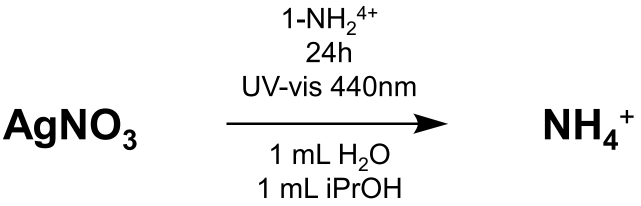

Figure S76. Scheme of the photocatalytic reaction mediated by 1-NH_2^{4+} in H_2O:iPrOH using $AgNO_3$ as the substrate.

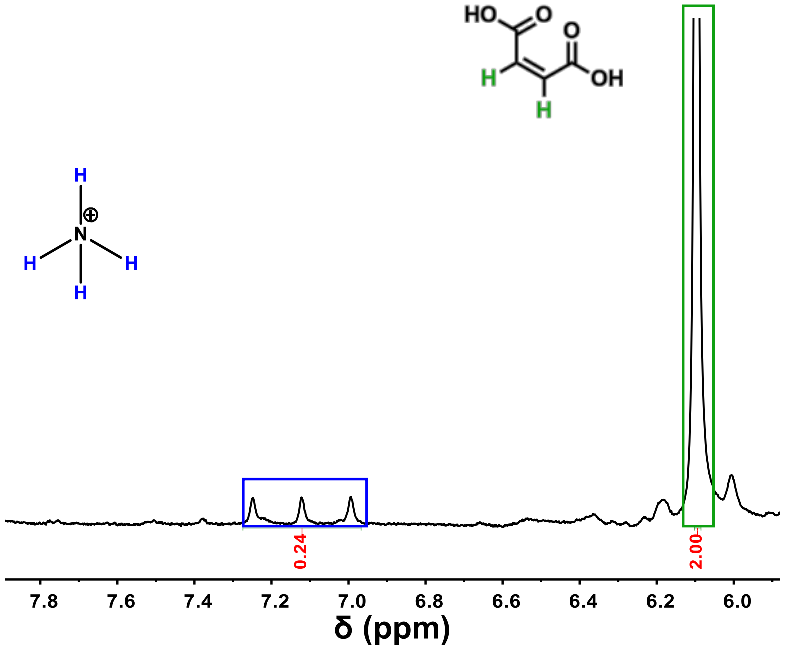

Figure S77. ^{1}H NMR spectra in DMSO-d6 of the photocatalytic reaction trying $AgNO_3$ in a mixture of H_2O:iPrOH, showing the ammonia signal (blue) and the internal standard signal (maleic acid, green).

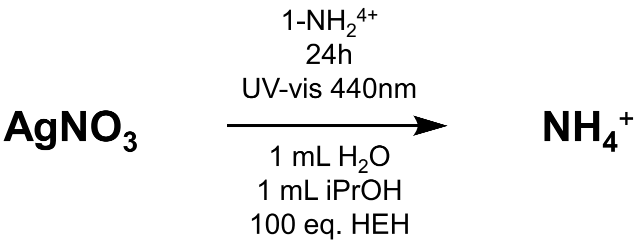

Figure S78. Scheme of the photocatalytic reaction mediated by 1-NH_2^{4+} with 100 equivalents of HEH in H_2O:iPrOH using $AgNO_3$ as the substrate.

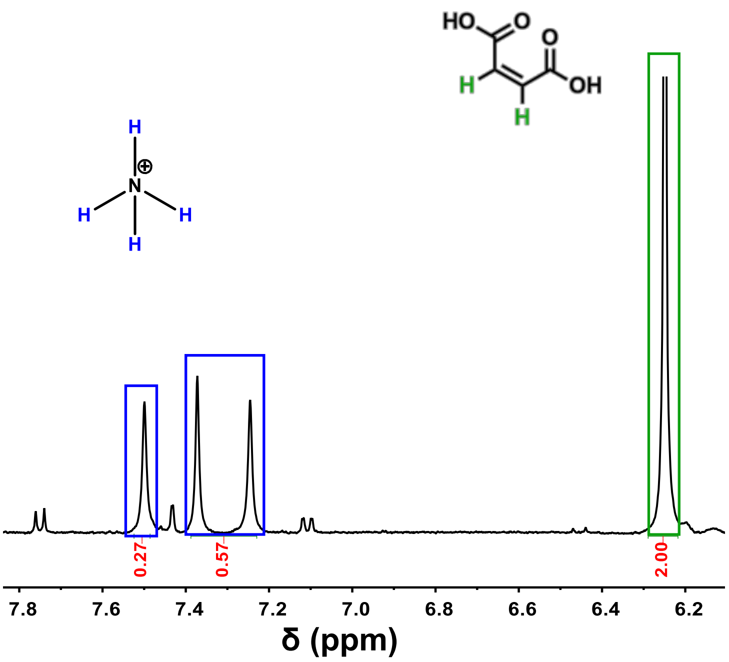

Figure S79. ^{1}H NMR spectra in DMSO-d6 of the photocatalytic reaction with 100 equivalents of HEH trying $AgNO_3$ in a mixture of H_2O:iPrOH, showing the ammonia signal (blue) and the internal standard signal (maleic acid, green).

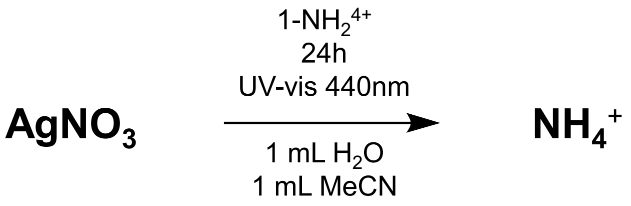

Figure S80. Scheme of the photocatalytic reaction mediated by 1-NH_2^{4+} in H_2O:MeCN using $AgNO_3$ as the substrate.

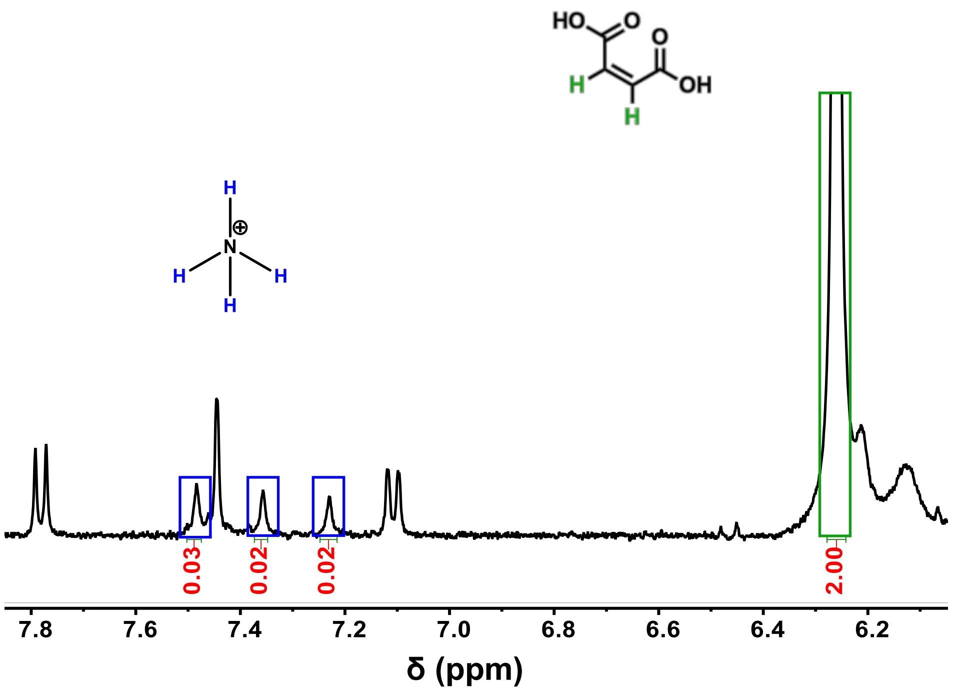

Figure S81. ^{1}H NMR spectra in DMSO-d6 of the photocatalytic reaction trying $AgNO_3$ in a mixture of H_2O:MeCN, showing the ammonia signal (blue) and the internal standard signal (maleic acid, green).

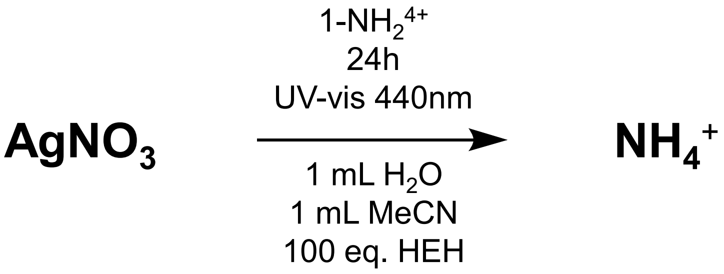

Figure S82. Scheme of the photocatalytic reaction mediated by 1-NH_2^{4+} with 100 equivalents of HEH in H_2O:MeCN using $AgNO_3$ as the substrate.

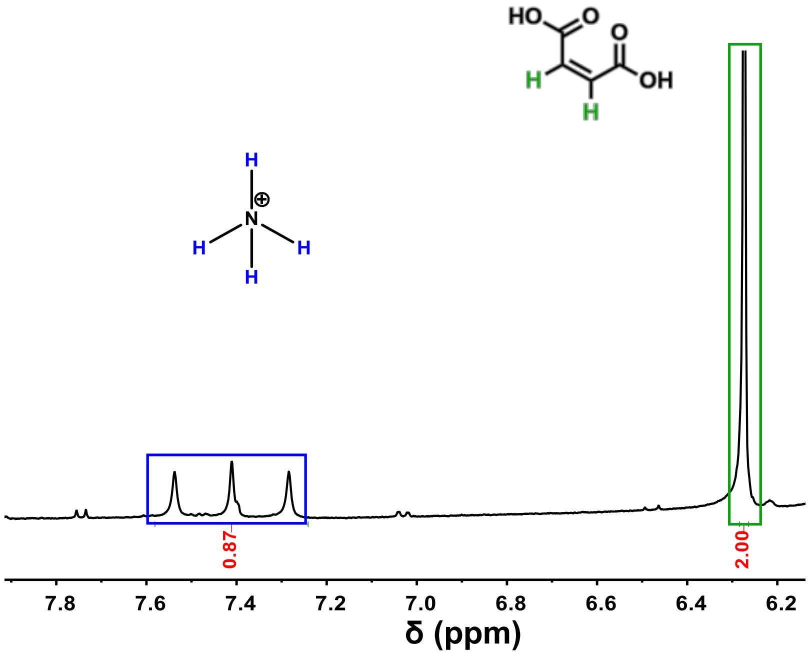

Figure S83. ^1H NMR spectra in DMSO-d6 of the photocatalytic reaction with 100 equivalents of HEH trying $AgNO_3$ in a mixture of H_2O:MeCN, showing the ammonia signal (blue) and the internal standard signal (maleic acid, green).

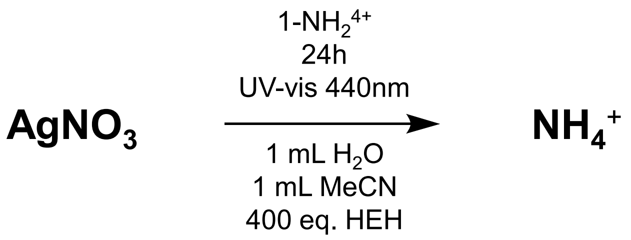

Figure S84. Scheme of the photocatalytic reaction mediated by 1-NH_2^{4+} with 400 equivalents of HEH in H_2O:MeCN using $AgNO_3$ as the substrate.

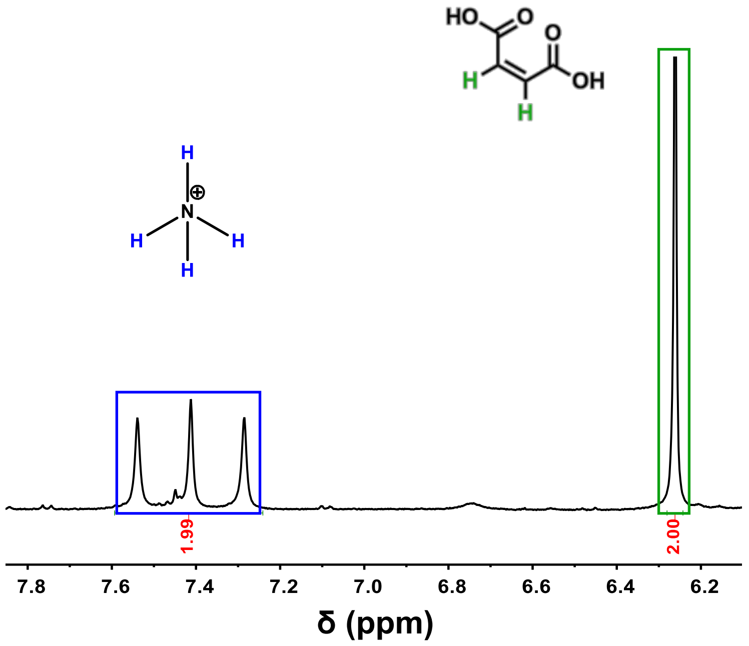

Figure S85. ^{1}H NMR spectra in DMSO-d6 of the photocatalytic reaction with 400 equivalents of HEH trying $AgNO_3$ in a mixture of H_2O:MeCN, showing the ammonia signal (blue) and the internal standard signal (maleic acid, green).

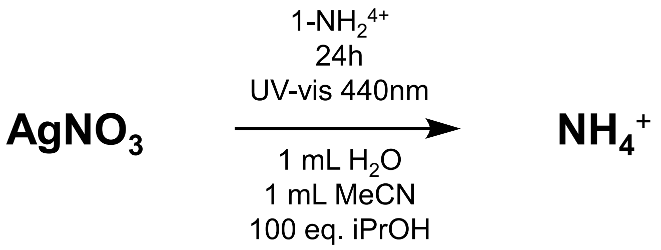

Figure S86. Scheme of the photocatalytic reaction mediated by 1-NH_2^{4+} with 100 equivalents of iPrOH in H_2O:MeCN using $AgNO_3$ as the substrate.

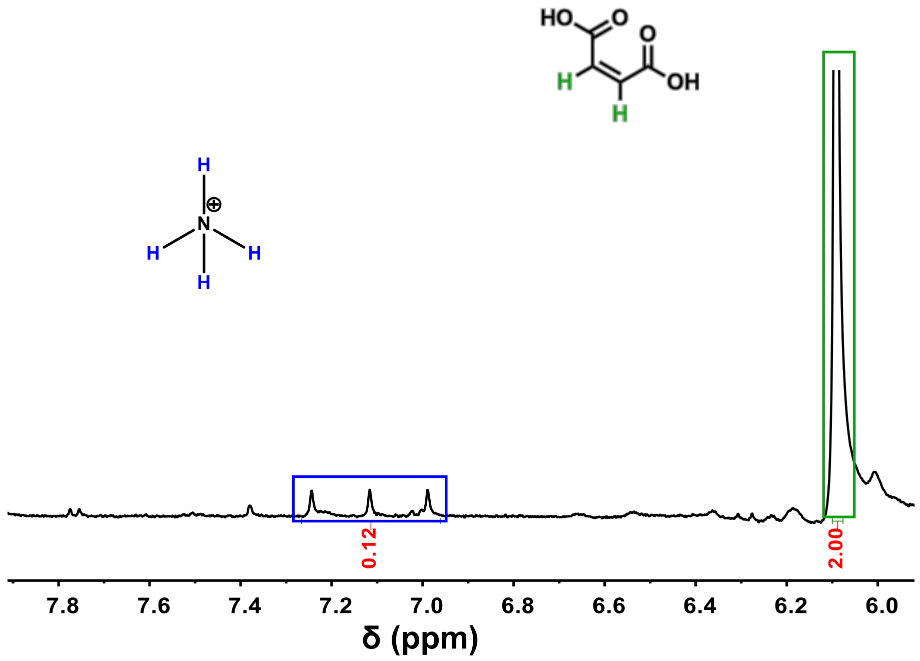

Figure S87. ^{1}H NMR spectra in DMSO-d6 of the photocatalytic reaction with 100 equivalents of iPrOH trying $AgNO_3$ in a mixture of H_2O:MeCN, showing the ammonia signal (blue) and the internal standard signal (maleic acid, green).

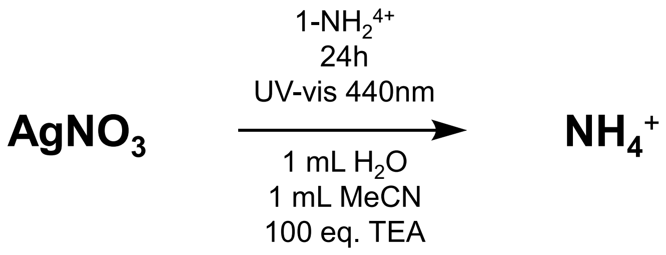

Figure S88. Scheme of the photocatalytic reaction mediated by 1-NH_2^{4+} with 100 equivalents of N-Et_3 in H_2O:MeCN using $AgNO_3$ as the substrate.

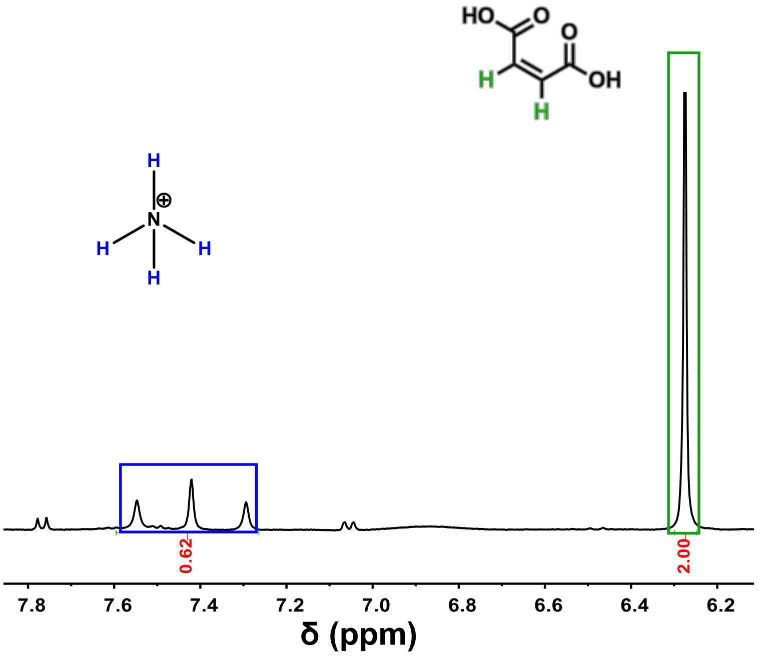

Figure S89. ^{1}H NMR spectra in DMSO-d6 of the photocatalytic reaction with 100 equivalents of N-Et_3 trying $AgNO_3$ in a mixture of H_2O:MeCN, showing the ammonia signal (blue) and the internal standard signal (maleic acid, green).

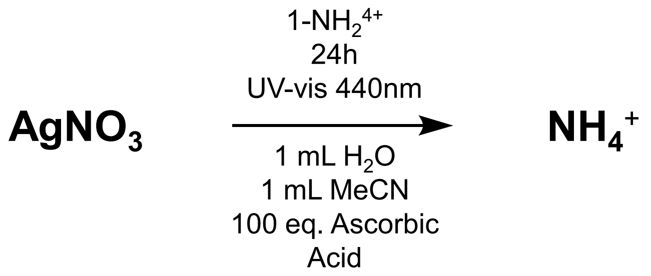

Figure S90. Scheme of the photocatalytic reaction mediated by 1-NH_2^{4+} with 100 equivalents of ascorbic acid in H_2O:MeCN using $AgNO_3$ as the substrate.

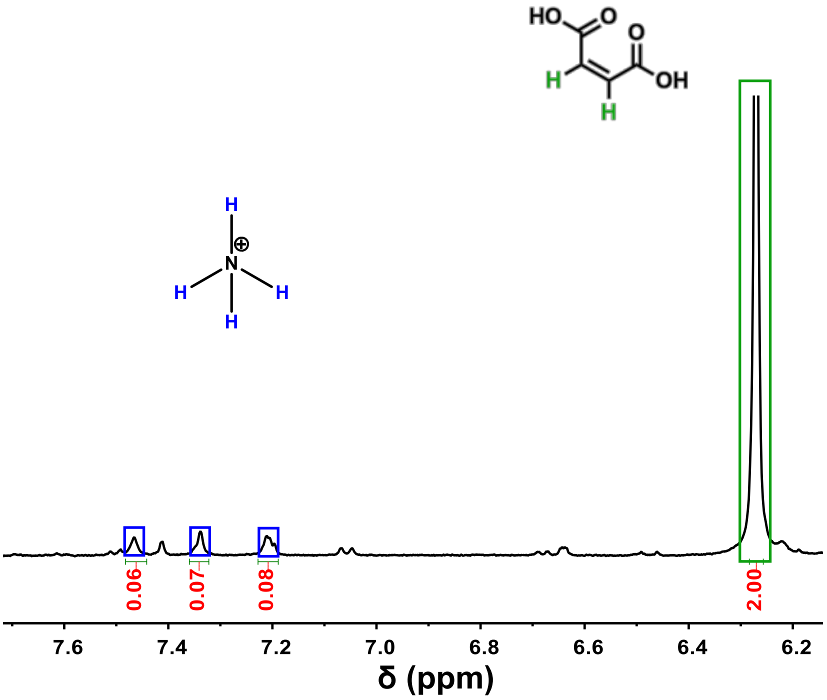

Figure S91. ^{1}H NMR spectra in DMSO-d6 of the photocatalytic reaction with 100 equivalents of ascorbic acid trying $AgNO_3$ in a mixture of H_2O:MeCN, showing the ammonia signal (blue) and the internal standard signal (maleic acid, green).

S.9.8. Combined Li^+ and Ag^+ catalysis

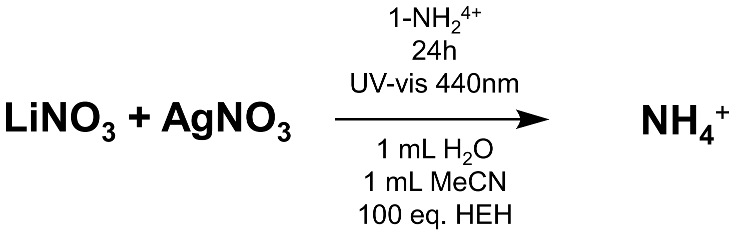

Figure S92. Scheme of the photocatalytic reaction mediated by $1\text{-}NH_2^{4+}$ with 100 equivalents of HEH in H_2O:MeCN using 35 equivalents of $LiNO_3$ and 15 equivalents of $AgNO_3$ as the substrates.

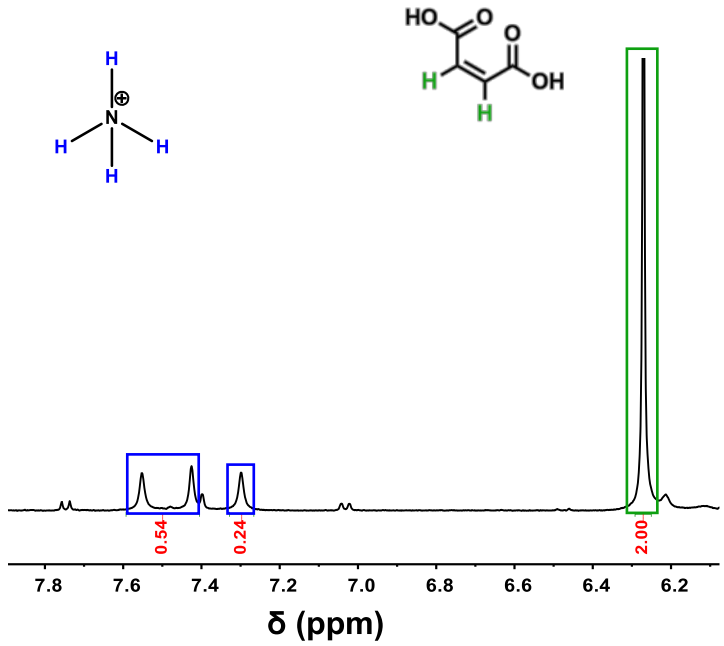

Figure S93. ^{1}H NMR spectra in DMSO-d6 of the photocatalytic reaction with 100 equivalents of HEH trying 35 equivalents of $LiNO_3$ and 15 equivalents of $AgNO_3$ in a mixture of H_2O:MeCN, showing the ammonia signal (blue) and the internal standard signal (maleic acid, green).

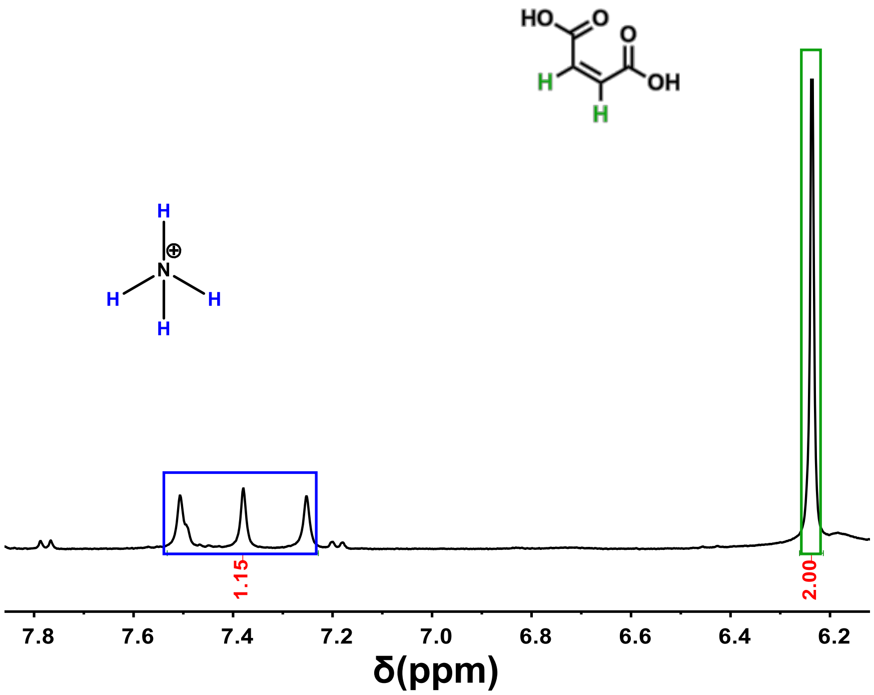

Figure S94. ^{1}H NMR spectra in DMSO-d6 of the photocatalytic reaction with 100 equivalents of HEH trying 35 equivalents of $LiNO_3$ and 15 equivalents of $AgNO_3$ in a mixture of H_2O:MeCN, showing the ammonia signal (blue) and the internal standard signal (maleic acid, green).

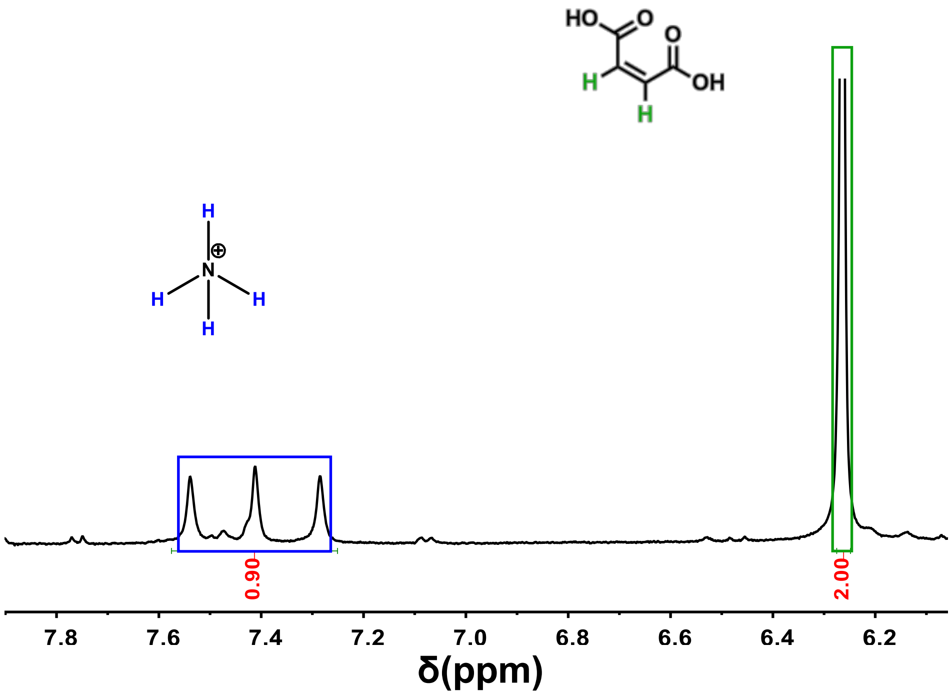

Figure S95. ^{1}H NMR spectra in DMSO-d6 of the photocatalytic reaction with 100 equivalents of HEH trying 35 equivalents of $LiNO_3$ and 15 equivalents of $AgNO_3$ in a mixture of H_2O:MeCN, showing the ammonia signal (blue) and the internal standard signal (maleic acid, green).

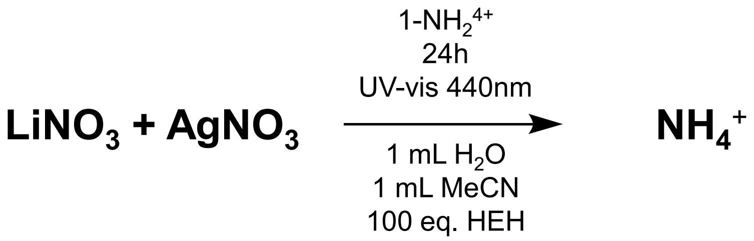

Figure S96. Scheme of the photocatalytic reaction mediated by 1-NH_2^{4+} with 100 equivalents of HEH in H_2O:MeCN using 25 equivalents of $LiNO_3$ and 25 equivalents of $AgNO_3$ as the substrates.

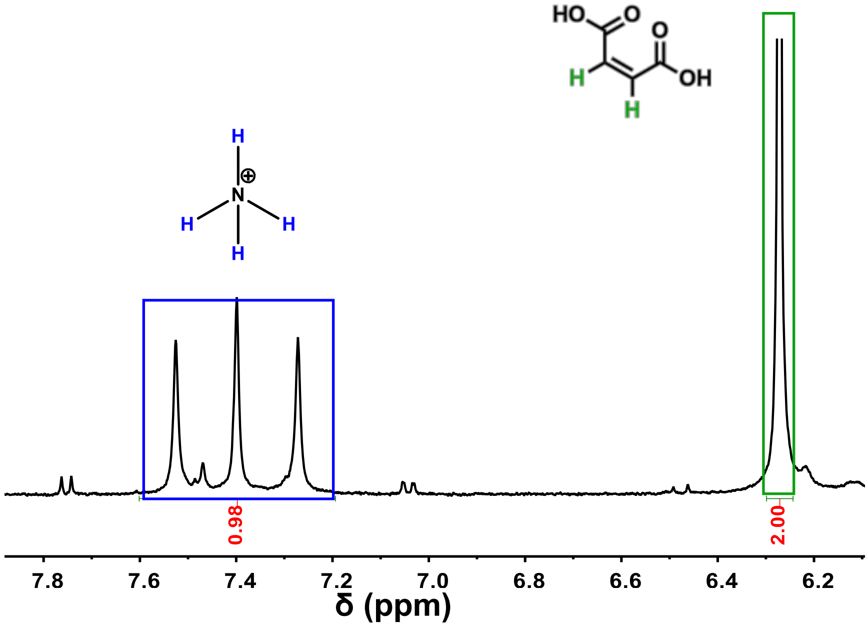

Figure S97. ^{1}H NMR spectra in DMSO-d6 of the photocatalytic reaction with 100 equivalents of HEH trying 25 equivalents of $LiNO_3$ and 25 equivalents of $AgNO_3$ in a mixture of H_2O:MeCN, showing the ammonia signal (blue) and the internal standard signal (maleic acid, green).

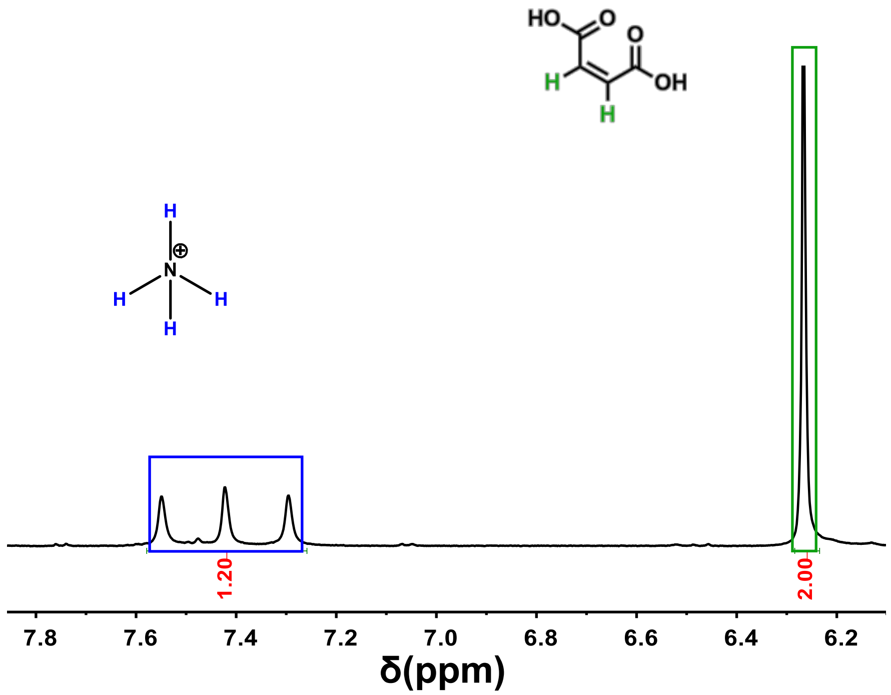

Figure S98. ^{1}H NMR spectra in DMSO-d6 of the photocatalytic reaction with 100 equivalents of HEH trying 25 equivalents of $LiNO_3$ and 25 equivalents of $AgNO_3$ in a mixture of H_2O:MeCN, showing the ammonia signal (blue) and the internal standard signal (maleic acid, green).

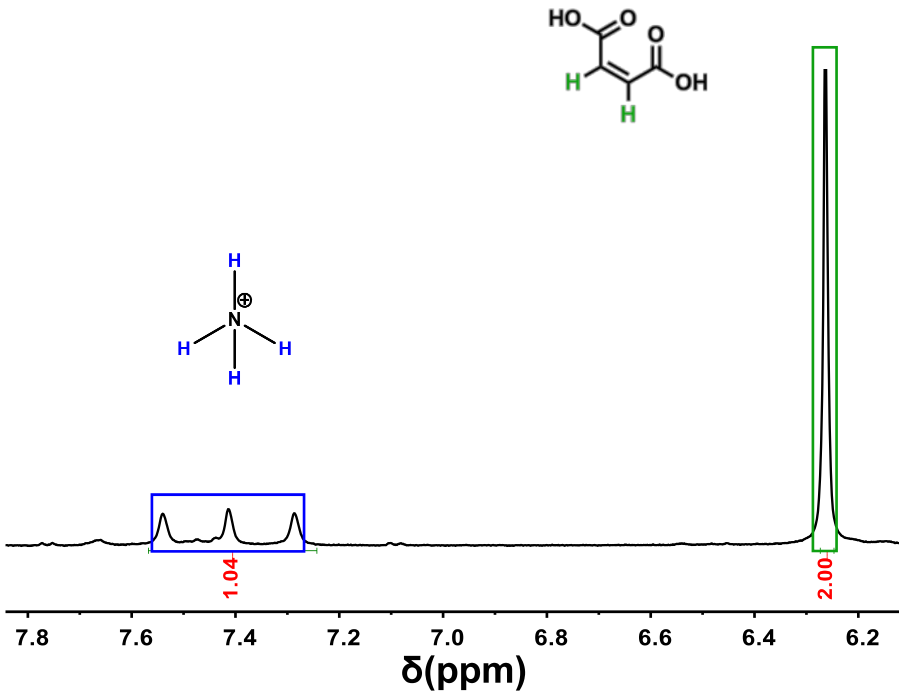

Figure S99. ^{1}H NMR spectra in DMSO-d6 of the photocatalytic reaction with 100 equivalents of HEH trying 25 equivalents of $LiNO_3$ and 25 equivalents of $AgNO_3$ in a mixture of H_2O:MeCN, showing the ammonia signal (blue) and the internal standard signal (maleic acid, green).

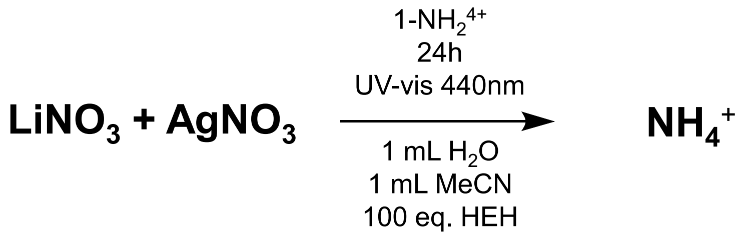

Figure S100. Scheme of the photocatalytic reaction mediated by 1-NH_2^{4+} with 100 equivalents of HEH in H_2O:MeCN using 15 equivalents of $LiNO_3$ and 35 equivalents of $AgNO_3$ as the substrates.

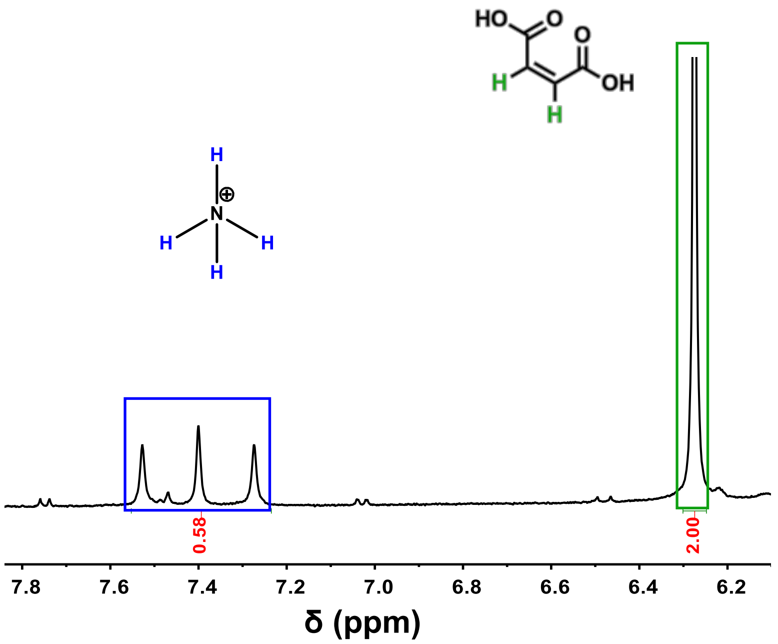

Figure S101. ^{1}H NMR spectra in DMSO-d6 of the photocatalytic reaction with 100 equivalents of HEH trying 15 equivalents of $LiNO_3$ and 35 equivalents of $AgNO_3$ in a mixture of H_2O:MeCN, showing the ammonia signal (blue) and the internal standard signal (maleic acid, green).

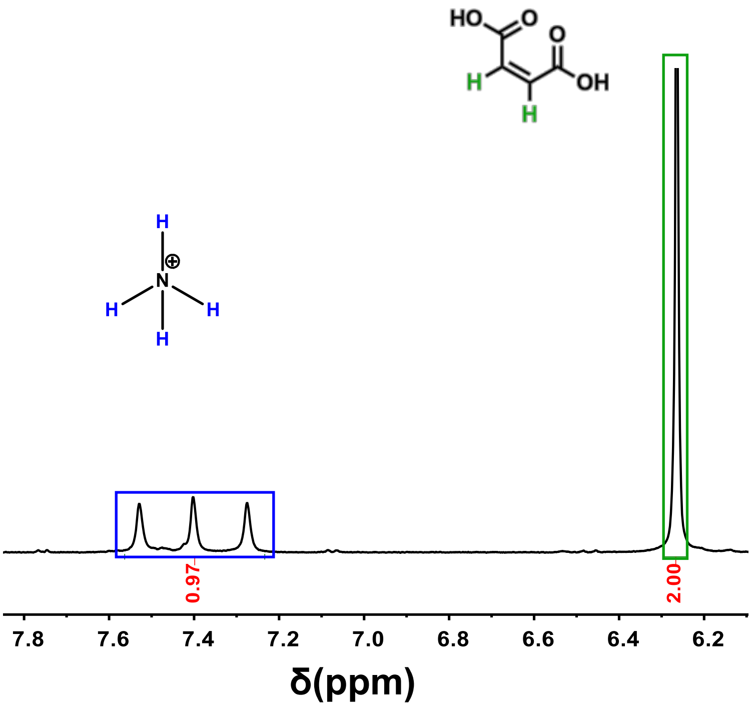

Figure S102. ^{1}H NMR spectra in DMSO-d6 of the photocatalytic reaction with 100 equivalents of HEH trying 15 equivalents of $LiNO_3$ and 35 equivalents of $AgNO_3$ in a mixture of H_2O:MeCN, showing the ammonia signal (blue) and the internal standard signal (maleic acid, green).

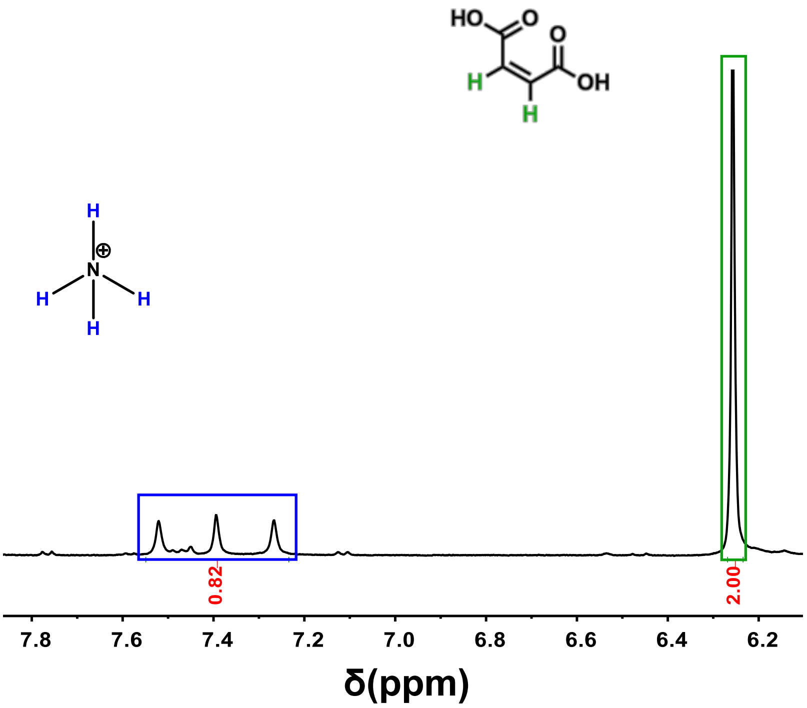

Figure S103. ^{1}H NMR spectra in DMSO-d6 of the photocatalytic reaction with 100 equivalents of HEH trying 15 equivalents of $LiNO_3$ and 35 equivalents of $AgNO_3$ in a mixture of H_2O:MeCN, showing the ammonia signal (blue) and the internal standard signal (maleic acid, green).

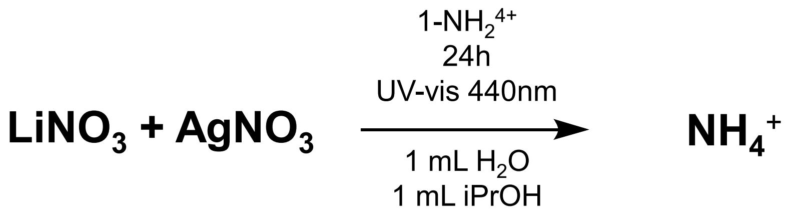

Figure S104. Scheme of the photocatalytic reaction mediated by 1-NH_2^{4+} in H_2O:iPrOH using 35 equivalents of $LiNO_3$ and 15 equivalents of $AgNO_3$ as the substrates.

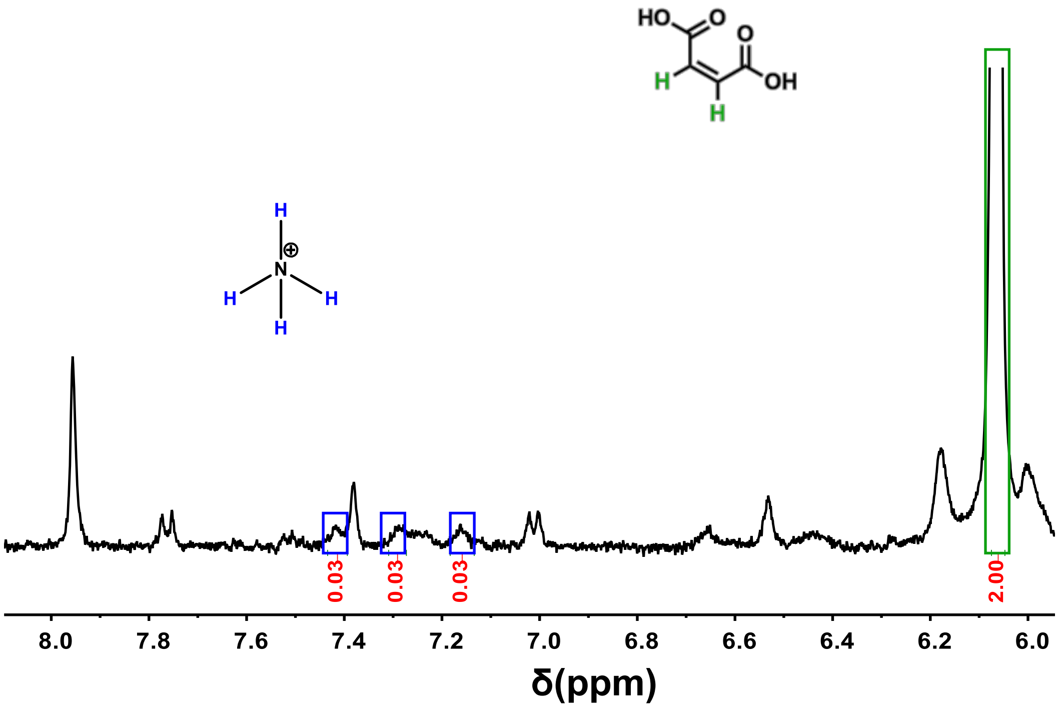

Figure S105. ^{1}H NMR spectra in DMSO-d6 of the photocatalytic reaction trying 35 equivalents of $LiNO_3$ and 15 equivalents of $AgNO_3$ in a mixture of H_2O:iPrOH, showing the ammonia signal (blue) and the internal standard signal (maleic acid, green).

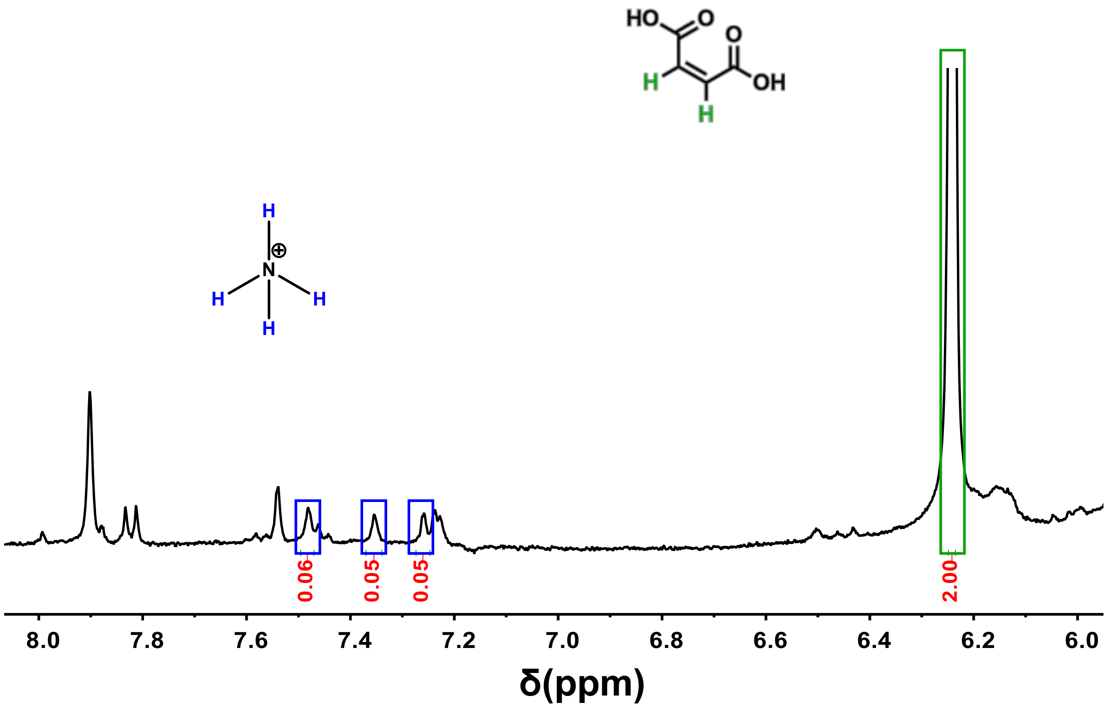

Figure S106. ^{1}H NMR spectra in DMSO-d6 of the photocatalytic reaction with trying 35 equivalents of $LiNO_3$ and 15 equivalents of $AgNO_3$ in a mixture of H_2O:iPrOH, showing the ammonia signal (blue) and the internal standard signal (maleic acid, green).

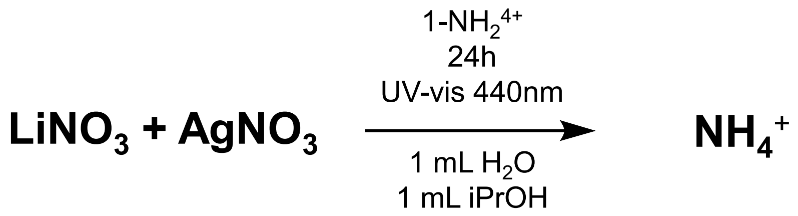

Figure S107. Scheme of the photocatalytic reaction mediated by 1-NH_2^{4+} in H_2O:iPrOH using 25 equivalents of $LiNO_3$ and 25 equivalents of $AgNO_3$ as the substrates.

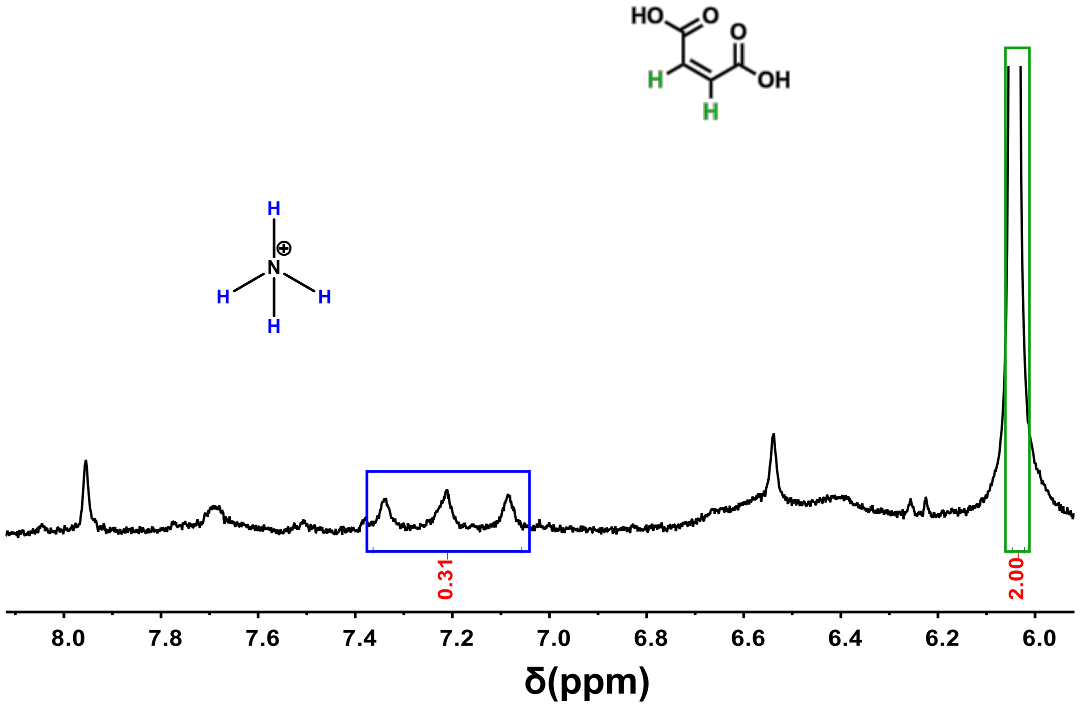

Figure S108. ^{1}H NMR spectra in DMSO-d6 of the photocatalytic reaction trying 25 equivalents of $LiNO_3$ and 25 equivalents of $AgNO_3$ in a mixture of H_2O:iPrOH, showing the ammonia signal (blue) and the internal standard signal (maleic acid, green).

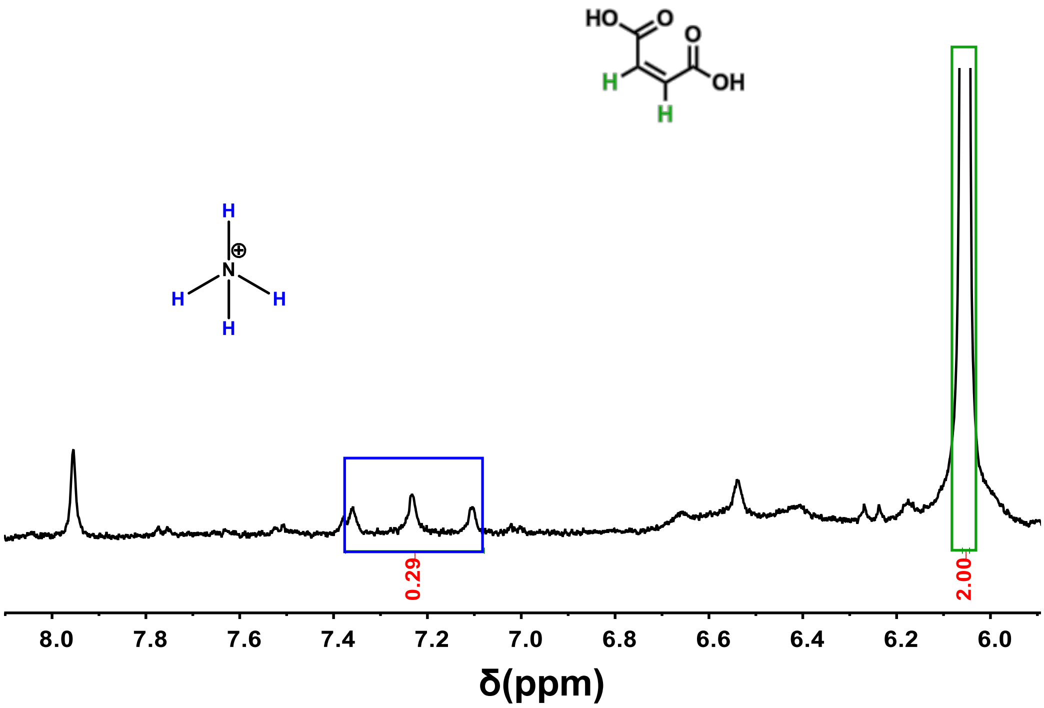

Figure S109. ^{1}H NMR spectra in DMSO-d6 of the photocatalytic reaction trying 25 equivalents of $LiNO_3$ and 25 equivalents of $AgNO_3$ in a mixture of H_2O:iPrOH, showing the ammonia signal (blue) and the internal standard signal (maleic acid, green).

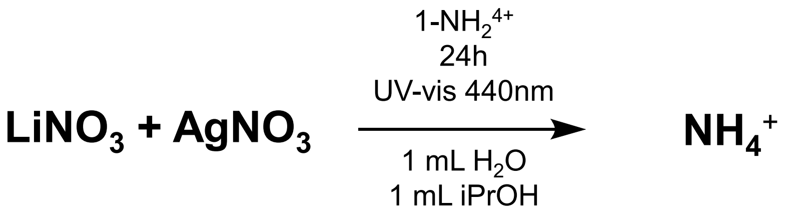

Figure S110. Scheme of the photocatalytic reaction mediated by 1-NH_2^{4+} in H_2O:iPrOH using 15 equivalents of $LiNO_3$ and 35 equivalents of $AgNO_3$ as the substrates.

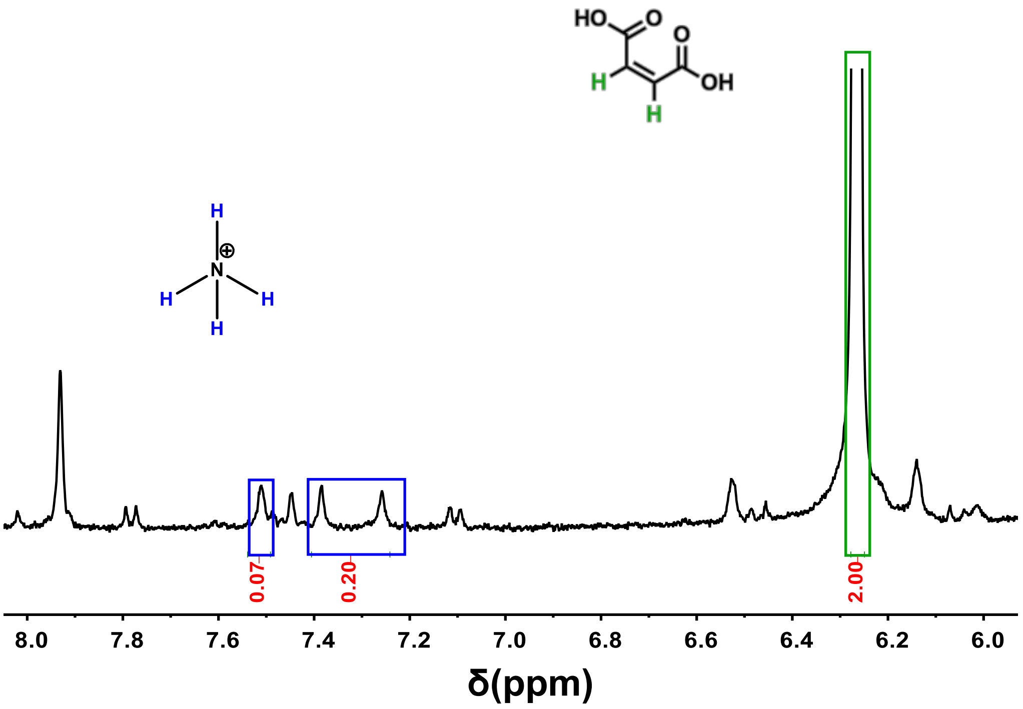

Figure S111. ^{1}H NMR spectra in DMSO-d6 of the photocatalytic reaction trying 15 equivalents of $LiNO_3$ and 35 equivalents of $AgNO_3$ in a mixture of H_2O:iPrOH, showing the ammonia signal (blue) and the internal standard signal (maleic acid, green).

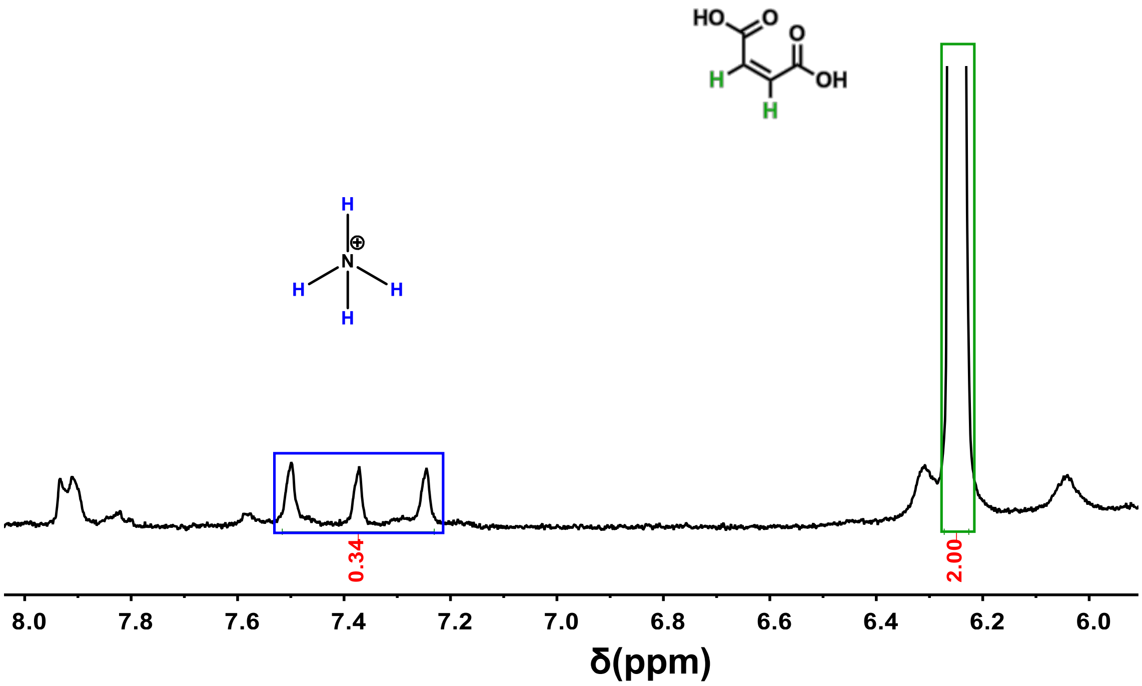

Figure S112. ^{1}H NMR spectra in DMSO-d6 of the photocatalytic reaction trying 15 equivalents of $LiNO_3$ and 35 equivalents of $AgNO_3$ in a mixture of H_2O:iPrOH, showing the ammonia signal (blue) and the internal standard signal (maleic acid, green).

S.10. DFT calculations

Complexation of Li^+ by $1 - NH_2^{4+}$:

Table S1. Theoretical energies of each Li^+ coordination state

Complexation zone	G^{o} (Eh) Water	G^{o} (Eh) Acetonitrile
No coordination	-13409.1125545	-13409.062392
A	-13409.147982	-13409.089389
B	-13409.147958	-13409.09556
C	-13409.157724	-13409.092082

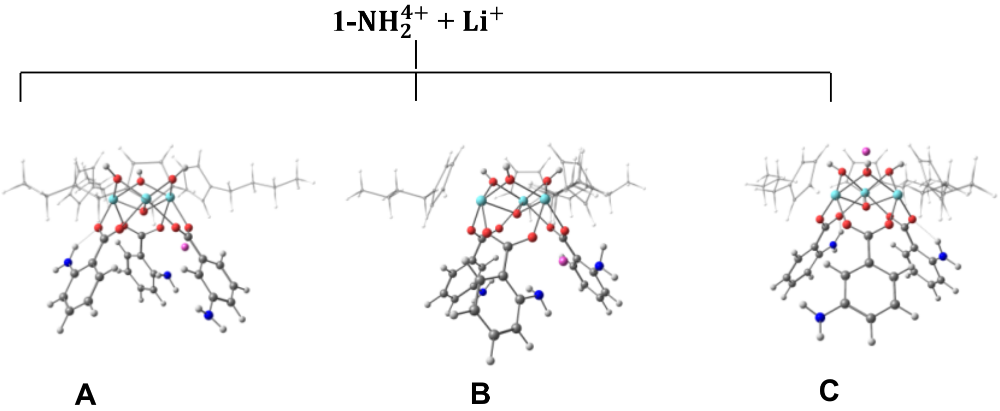

Figure S113. Theoretical structure of each Li^+ coordination state.

Table S2. Free energies of each Li^+ coordination state.

Solvent	A: ΔG^o (kcal/mol)	B: ΔG^o (kcal/mol)	C: ΔG^o (kcal/mol)
**Water**	-22.230	-22.215	-28.344
**Acetonitrile**	-16.941	-20.810	-18.631

Complexation of $LiNO_3$ by 1-NH_2^{4+}:

Table S3. Free energies of each $LiNO_3$ coordination state.

Complexation zone	G^{o} (Eh) Water	G^{o} (Eh) Acetonitrile
No coordination	-13689.438494	-13689.386002
D	-13689.45005	-13689.394864
E	-13689.464241	-13689.406726

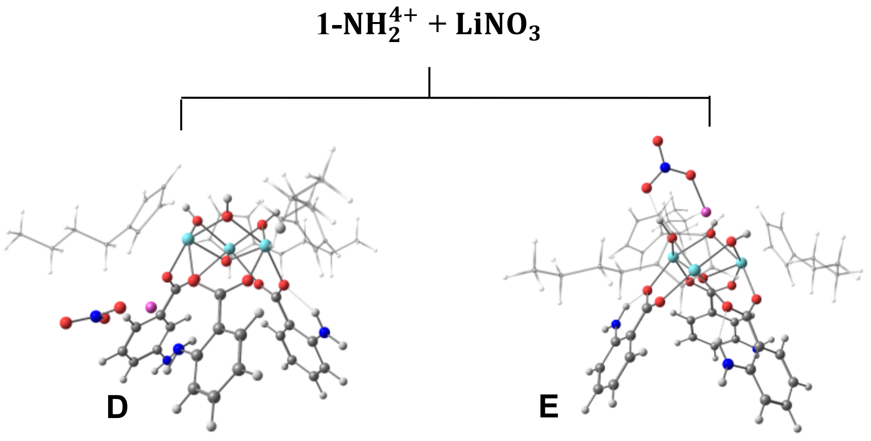

Figure S114. Theoretical structure of each $LiNO_3$ coordination state.

Table S4. Free energies of each $LiNO_3$ coordination state.

Solvent	D: ΔG° (kcal/mol)	E: ΔG° (kcal/mol)
**Water**	-7.251	-5.561
**Acetonitrile**	-16.156	-13.004

S.11. References

[1] Gaussian 09, Revision D.01, M. J. Frisch; G. W. Trucks, H. B. Schlegel, G. E. Scuseria, M. A. Robb, J. R. Cheeseman, G. Scalmani, V. Barone, B. Mennucci, G. A. Petersson, H. Nakatsuji, M. Caricato, X. Li, H. P. Hratchian, A. F. Izmaylov, J. Bloino, G. Zheng, J. L. Sonnenberg, M. Hada, M. Ehara, K. Toyota, R. Fukuda, J. Hasegawa, M. Ishida, T. Nakajima, Y. Honda, O. Kitao, H. Nakai, T. Vreven, J. A. Montgomery, Jr., J. E. Peralta, F. Ogliaro, M. Bearpark, J. J. Heyd, E. Brothers, K. N. Kudin, V. N. Staroverov, R. Kobayashi, J. Normand, K. Raghavachari, A. Rendell, J. C. Burant, S. S. Iyengar, J. Tomasi, M. Cossi, N. Rega, J. M. Millam, M. Klene, J. E. Knox, J. B. Cross, V. Bakken, C. Adamo, J. Jaramillo, R. Gomperts, R. E. Stratmann, O. Yazyev, A. J. Austin, R. Cammi, C. Pomelli, J. W. Ochterski, R. L. Martin, K. Morokuma, V. G. Zakrzewski, G. A. Voth, P. Salvador, J. J. Dannenberg, S. Dapprich, A. D. Daniels, Ö. Farkas, J. B. Foresman, J. V. Ortiz, J. Cioslowski, and D. J. Fox, Gaussian, Inc., Wallingford CT, 2009.

[2] Tao, J.; Perdew, J. P.; Staroverov, V. N.; Scuseria, G. E. *Phys. Rev. Lett.* **2003**, *91* (14), 146401

[3] Weigend, F.. *Phys. Chem. Chem. Phys.* **2006**, *8* (9), 1057–1065.

[4] Weigend, F.; Ahlrichs, R.. *Phys. Chem. Chem. Phys.* **2005**, *7* (18), 3297–3305.

[5] Marenich, A. V.; Cramer, C. J.; Truhlar, D. G. *J. Phys. Chem. B* **2009**, *113* (18), 6378–6396.

[6] Álvarez-Moreno, M.; de Graaf, C.; Lopez, N.; Maseras, F.; Poblet, J.M.; Bo, C. *J. Chem. Inf. Model*. **2015**, *55*, 95-103.

[7] Delgado, P.; Martin-Romera, J. D.; Perona, C.; Vismara, R.; Galli, S.; Maldonado, C. R.; Carmona, F. J.; Padial, N. M.; Navarro, J. A. R. *ACS Appl. Mater*. **2022**, *14*, 26501–26506.

[8] P. J. Jabalera-Ortiz, C. Perona, M. Moreno-Albarracín, F. J. Carmona, J.-R. Jiménez, J. A. R. Navarro, P. Garrido-Barros, *Angew. Chem. Int. Ed.* 2024, e202411867. https://doi.org/10.1002/anie.202411867.

[9] B. H. R. Suryanto, K. Matuszek, J. Choi, R. Y. Hodgetts, H.-L. Du, J. M. Bakker, C. S. M. Kang, P. V. Cherepanov, A. N. Simonov, D. R. MacFarlane, *Science* **2021**, *372*, 1187.
